# The prevalence and burden of heavy menstrual bleeding, and market access barriers of medical interventions with a focus on low- and middle-income countries: a scoping review

**DOI:** 10.1186/s12905-025-04157-5

**Published:** 2025-11-29

**Authors:** Anne Ammerdorffer, Odyssée Ferrillo, Kate Mills, Tahlia Guneratne, Maureen Makama, Lorena Romero, Annie R.A. McDougall, Jen Sothornwit, Pisake Lumbiganon, M. Valeria Bahamondes, Jennifer Scott, Lester Chinery, Luis Bahamondes, Joshua P. Vogel, A. Metin Gülmezoglu

**Affiliations:** 1 Concept Foundation, Bâtiment F2F3 Avenue de Sécheron 15, Geneva, 1202 Switzerland; 2 Women’s, Children’s and Adolescents’ Health Program, Burnet Institute, Melbourne, Australia; 3 Health and Social Care Unit, School of Public Health and Preventive Medicine, Monash University, Melbourne, Australia; 4 The Ian Potter Library, Alfred Health, Melbourne, Australia; 5 Monash Institute of Pharmaceutical Sciences, Monash University, Parkville, Australia; 6 Department of Obstetrics and Gynecology, Faculty of Medicine, Khon Kaen University, Khon Kaen, Thailand; 7 Independent Sexual and Reproductive Health Consultant, Austin, TX United States of America; 8 Department of Obstetrics & Gynaecology, Beth Israel Deaconess Medical Center, Boston, MA USA; 9 Department of Obstetrics and Gynaecology, University of Campinas Faculty of Medical Sciences, Campinas, Brazil

**Keywords:** Heavy menstrual bleeding, Abnormal uterine bleeding, Prevalence, Burden, Treatment, Low- and middle-income countries, Scoping review, Menstrual health, Women’s health

## Abstract

**Background:**

Heavy menstrual bleeding (HMB) is defined as excessive menstrual blood loss that negatively affects physical, emotional, social, and quality of life (QoL) of women at reproductive age. The aim of this systematic scoping review was to map and summarize the available evidence on the prevalence, burden, treatment, and barriers to accessing treatment for HMB in low- and middle-income countries (LMICs).

**Methods:**

We conducted a review of published data in nine databases and included studies published from 1 January 2000 to 28 October 2024. Covidence was used for screening and data extraction.

**Results:**

We identified 376 eligible studies conducted in 46 countries. Most of the studies (300, 80%) reported on the treatment of HMB (including 9 studies on treatment and burden), 55 reported on prevalence, 19 reported on burden (including 12 studies on burden and prevalence) and two studies reported on barriers to providing treatment. A total of 55 systematic reviews were included. Prevalence of HMB ranged from 5% to 58%, using a variety of diagnostic and reporting methods. Burdens of HMB included impaired QoL, mental health issues, economic burdens, and health concerns such as anemia. The use of a hormonal intrauterine device was the most commonly reported treatment for HMB (*n* = 120), followed by progestin (*n* = 41), combined oral contraceptives (*n* = 36), gonadotrophin-releasing hormone (*n* = 31), and tranexamic acid (*n* = 23). Thirty studies focused on herbal treatment. Lack of knowledge and misunderstanding of treatment options for HMB were mentioned as barriers for HMB treatment.

**Conclusion:**

The prevalence of HMB and the variety of treatment options available indicate that HMB is a condition that should not be underestimated and has a large impact on women’s health and well-being. There is a need for reliable, good-quality studies on the prevalence, burden, and treatment access barriers of HMB, especially in LMICs where HMB more often leads to iron deficiency anaemia.

**Supplementary Information:**

The online version contains supplementary material available at 10.1186/s12905-025-04157-5.

## Background

Heavy menstrual bleeding (HMB) is defined as excessive menstrual blood loss that interferes with the physical, emotional, social, and quality of life (QoL) of women at reproductive age [1, 2]. HMB can occur alone or in combination with other symptoms, including dysmenorrhea (pain during menstruation), fatigue or headache [1, 2] and can lead to other serious health issues such as iron deficiency anemia (IDA), which occurs in one in four women with HMB [3]. HMB is objectively defined as menstrual blood loss of 80 mL or more per cycle not attributable to pregnancy, systemic condition (for example, bleeding or thyroid disorders) or gynecological disease [4, 5]. However, women often seek treatment for HMB without meeting this specific amount of blood loss. Hence a broader definition of HMB is currently used [1, 6]. According to this definition, HMB can present with any of the following symptoms: (a) bleeding lasts more than 7 days; (b) soaking through one or more tampons or pads every hour for several consecutive hours; (c) needing to wear more than one pad at a time to manage menstrual flow; (d) needing to change pads or tampons during the night; or (e) menstrual flow containing blood clots as big as a American quarter dollar or larger [7, 8].

Multiple causes of HMB exist, most commonly uterine fibroids, but it can also be due to ovulatory disorders, adenomyosis, endometriosis, hematological disorders, endometrial polyps, menopausal transition, and endometrial hyperplasia or malignancy [9–11]. Diverse treatments for HMB are available with different effectiveness, tolerability, acceptability, and costs. For an individual woman, the preferred treatment options will depend on the specific cause, her age, associated symptoms, intention to get pregnant, and personal preferences. First-line treatment includes medical therapies such as non-steroidal anti-inflammatory drugs (NSAIDs), antifibrinolytic agents, combined hormonal contraceptives (COCs), cyclical oral progestogens, 52-mg levonorgestrel-releasing intrauterine device (LNG-IUD), danazol, and ethamsylat [12, 13]. Second-line therapies are surgical interventions including myomectomy, endometrial resection and ablation, and hysterectomy [12]. Accessible and affordable interventions to reduce menstrual blood loss can not only improve QoL for women experiencing HMB but may also prevent or reduce anemia in reproductive age women [14].

HMB and its consequences for developing IDA, is a worldwide health problem, and a particular challenge for women in low- and middle-income countries (LMICs), who may often lack access to effective, quality-assured and affordable HMB treatments. In many LMICs, the intake of iron-rich food can be limited, and the low bioavailability of those foods and decreased nutrient absorption are more common [15]. The prevalence of IDA amongst reproductive-age women in LMICs is 30% [16]. In women with IDA, iron stores in the body may be depleted even with modest amounts of blood loss [16]. Furthermore, after effective treatment of HMB, many women need up to three years to replenish their iron stores if no other intervention is administered [17].

In recent years, several systematic and scoping reviews have been published on HMB. These include reviews on pictorial methods to assess HMB [18], evaluation of Health-Related Quality of Life (HRQoL) instruments related to abnormal uterine bleeding (AUB) [19], and a review to identify interventions that can improve access to care for AUB [20]. An extensive 2022 review provided an overview of Cochrane reviews and network meta-analysis on interventions for HMB [12]. While some of these reviews focus specifically on HMB, many of them address AUB in general which has a broader definition than HMB alone. In addition, certain reviews do focus on HMB but include only one underlying cause such as uterine fibroids or another specific condition [21–23]. The prevalence and burden of HMB, the medical interventions for its treatment, and the market access to these treatment options are three important topics needed to comprehensively understand HMB in LMICs. The aim of this scoping review is to bring these aspects together and provide a complete overview of HMB, with a focus on women and communities in LMICs.

## Materials and methods

We carried out the scoping review following the methodology initially developed by Arksey and O’Malley, further adapted by Levac et al. [24]. This is a six-stage methodological framework, of which we implemented the first five steps: (1) identifying the research question (2), searching for relevant studies (3), selecting studies (4), charting the data and collating, and (5) summarizing and reporting the results. Findings of this review are reported in accordance with the Preferred Reporting Items for Systematic Reviews and Meta-Analyses Extension for Scoping Reviews (PRISMA-ScR) checklist [25] (Supplement 1). The protocol is registered online on Open Science Framework, available at https://osf.io/65t2f/.

### Eligibility criteria

We included prevalence and burden of disease studies on HMB, in adolescents and adults, that were conducted in countries classified as low-income, lower middle-income, or upper middle-income according to the 2024 World Bank classification of countries by income level [26]. No geographical restrictions were applied for systematic reviews. Additionally, we included all studies (no country limitations) that explored medical interventions for HMB, including NSAIDs, antifibrinolytics (such as tranexamic acid), COCs, combined vaginal ring (CVR), long-cycle and luteal oral progestogens, IUDs, as well as herbal and traditional medicines. We restricted the scope of this review to medical interventions for HMB since these are the first line of intervention for most women. We included primary research using multiple study designs, such as interventional trials (both randomized and non-randomized), observational studies (including prospective cohort, retrospective, cross-sectional, and case-control studies), qualitative studies, mixed methods studies, as well as systematic reviews and meta-analyses of primary research. We included structural causes of HMB, such as fibroids, polyps, and adenomyosis, as long as the studies reported on HMB as one of the outcomes. We included studies that presented data concerning the burden of HMB on various health-related aspects, including QoL, Disability-adjusted Life Years (DALY), and HRQoL. We excluded studies related to HMB that were not associated with gynecologic causes, such as HMB resulting from cancer treatment, genetic disorders, or anticoagulant usage. Studies concerning endometriosis were also excluded. Treatment studies were excluded if they did not report data pertaining to their impact on HMB within the context of the study (for example, studies that only report on fibroid size and not on the reduction of bleeding after treatment). We excluded all studies that focused exclusively on surgical interventions, including open (abdominal or vaginal), minimally invasive (laparoscopic) and unspecified (or surgeon’s choice of) routes of hysterectomy, resectoscopic endometrial ablation (REA), non-resectoscopic endometrial ablation (NREA), and unspecified endometrial ablation. Studies using the following study designs were excluded: commentaries, or editorial articles that do not present primary data, narrative reviews, case studies, case reports and book chapters. Guidelines and management studies were also excluded as were studies discussing non-contraceptive benefits of contraceptive methods (such as reducing HMB). Additionally, studies for which full-text articles were inaccessible or unavailable were excluded, as were papers not written in English or French.

### Electronic searches

Searches were conducted in nine electronic databases: Ovid Embase, Ovid MEDLINE, WHO Global Index Medicus, Ovid Global Health, Ovid Emcare, Ovid PsycINFO, Scopus, Web of Science, and CINAHL to identify published peer-reviewed articles. Papers published between 1 st January 2000 to 28th October 2024 were screened for this review. An information specialist was consulted to develop the search strategies (LR). The reference lists of all included full text studies and any systematic reviews identified in the primary yield were manually screened and assessed for additional eligible studies not identified by the search. The complete search strategy can be found in Supplement 2.

### Study selection

**Fig. 1:**
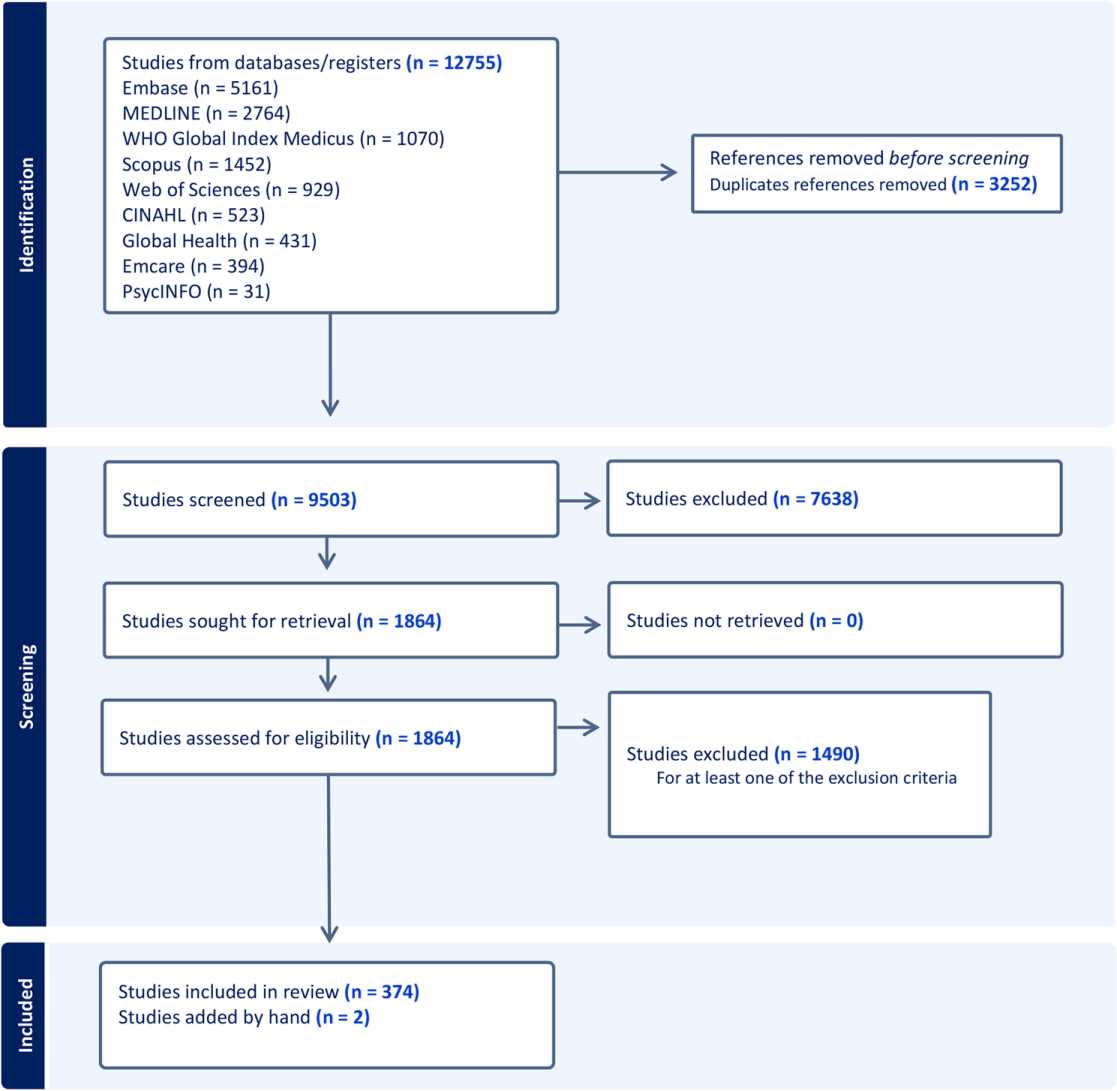
Flowchart of study selection. A total of 12755 studies were retrieved from nine databases, of which 3252 duplicates were removed. Based on title and abstract screening, 7638 studies were excluded, followed by the exclusion of 1490 studies after full text reviewing

The search results were exported into Endnote for data management. After duplicates were removed, data was imported to Covidence for screening and data extraction. Title and abstract screening were performed by a single reviewer (AA or MM) to identify studies relevant to the scope of the review. In the second stage, full-text screening was performed by two independent reviewers (AA, OF, JS, PL, LB, VB, KM, TG, or MM) to determine which studies met the eligibility criteria. Uncertainties about study inclusion or disagreements among reviewers were discussed with the broader team. Citation screening and selection was documented and summarized in a PRISMA-compliant flow chart (Fig. 1).

### Data extraction and analysis

We created a standardized data extraction template within Covidence. The template facilitated the systematic extraction of pertinent information from each study, including the title, authors, year of publication, countries studied, sample size, study characteristics, methodology, intervention, outcome measures, and main findings. Data was extracted by one reviewer (AA, OF, KM, or TG).

## Results

We retrieved 12,755 records from nine different databases. After de-duplication, 9,503 records remained for title and abstract screening. Subsequently, 1,864 records were deemed suitable for full text screening, of which 1,490 records were excluded. Two studies identified from the references of a narrative review were included for full text screening. Overall, a total of 376 unique studies were included in the scoping review (Fig. 1).

### Characteristics of all included studies

Of the 376 included studies, 291 (77.4%) reported information on the treatment of HMB, 55 (14.6%) reported on prevalence, seven (1.9%) reported on burden, and two (0.5%) studies reported on barriers to accessing treatment. Nine studies reported data on burden and treatment (2.4%, analyzed under treatment) and 12 reported on burden and prevalence (3.2%, analyzed under burden). A total of 55 systematic reviews were included. Forty-nine systematic reviews (89.1%) reported on HMB treatment, four on prevalence, one on burden, and one on burden and treatment.

**Fig. 2:**
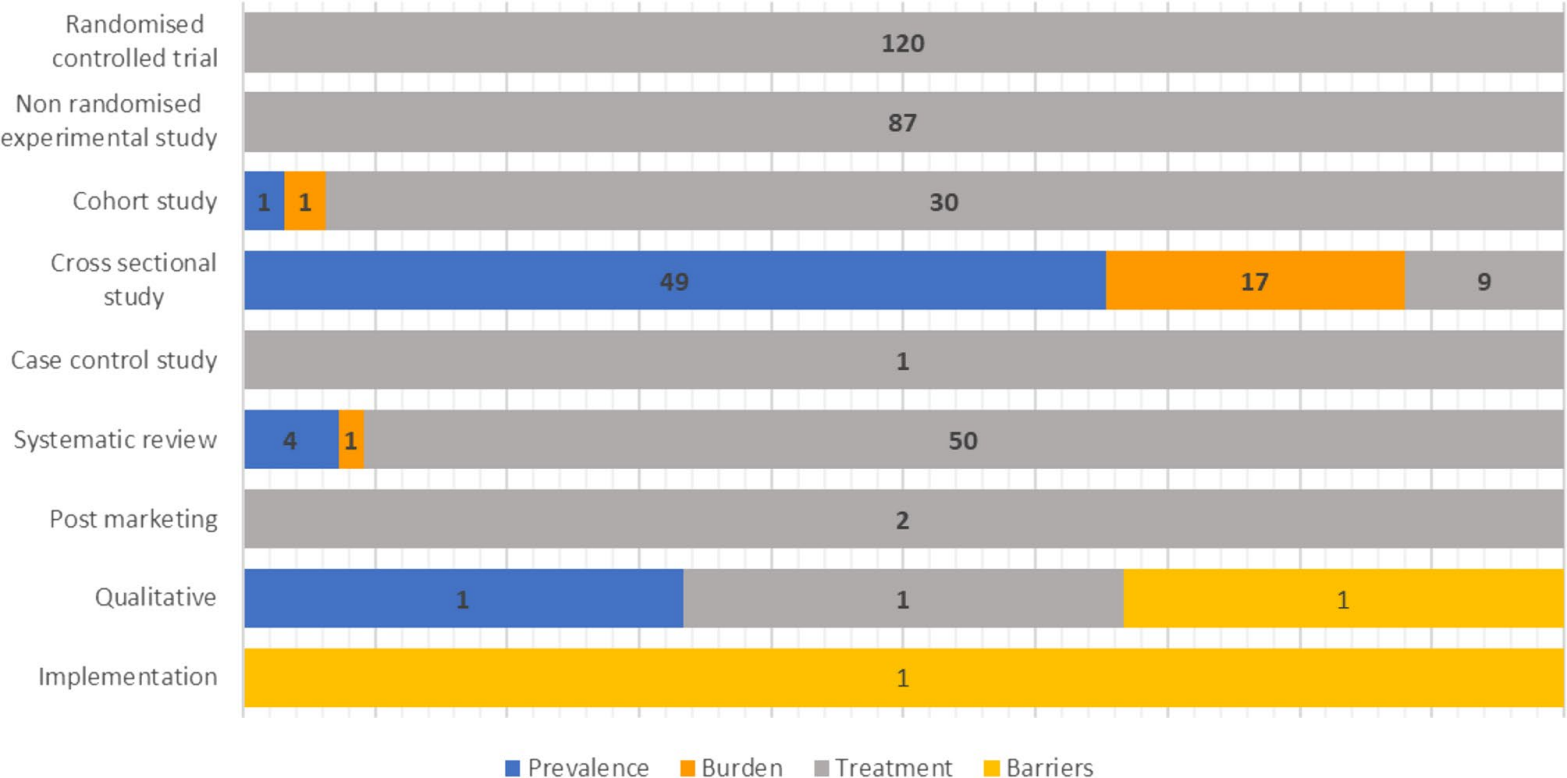
Distribution of studies reporting prevalence, burden, treatment, and treatment barriers of HMB by study design

A total of 207 studies (55.0%) were interventional studies (Fig. 2). These comprised randomized trials (120 studies, 31.9%) and non-randomized experimental studies (87 studies, 23.1%). In total, 108 studies used observational designs (28.7%), including cross-sectional studies (75 studies, 19.9%), cohort studies (32 studies, 8.5%) and one case-control study (0.3%). The remaining studies included 55 systematic reviews (14.6%), two post-marketing studies (0.5%), three qualitative studies (0.8%) and one implementation study (0.3%). Treatment data were reported across all types of study designs, while burden and prevalence were described in cross-sectional studies, cohort studies and systematic reviews (Fig. 2). One implementation study and one qualitative research study reported on the barriers to accessing treatment.

**Fig. 3:**
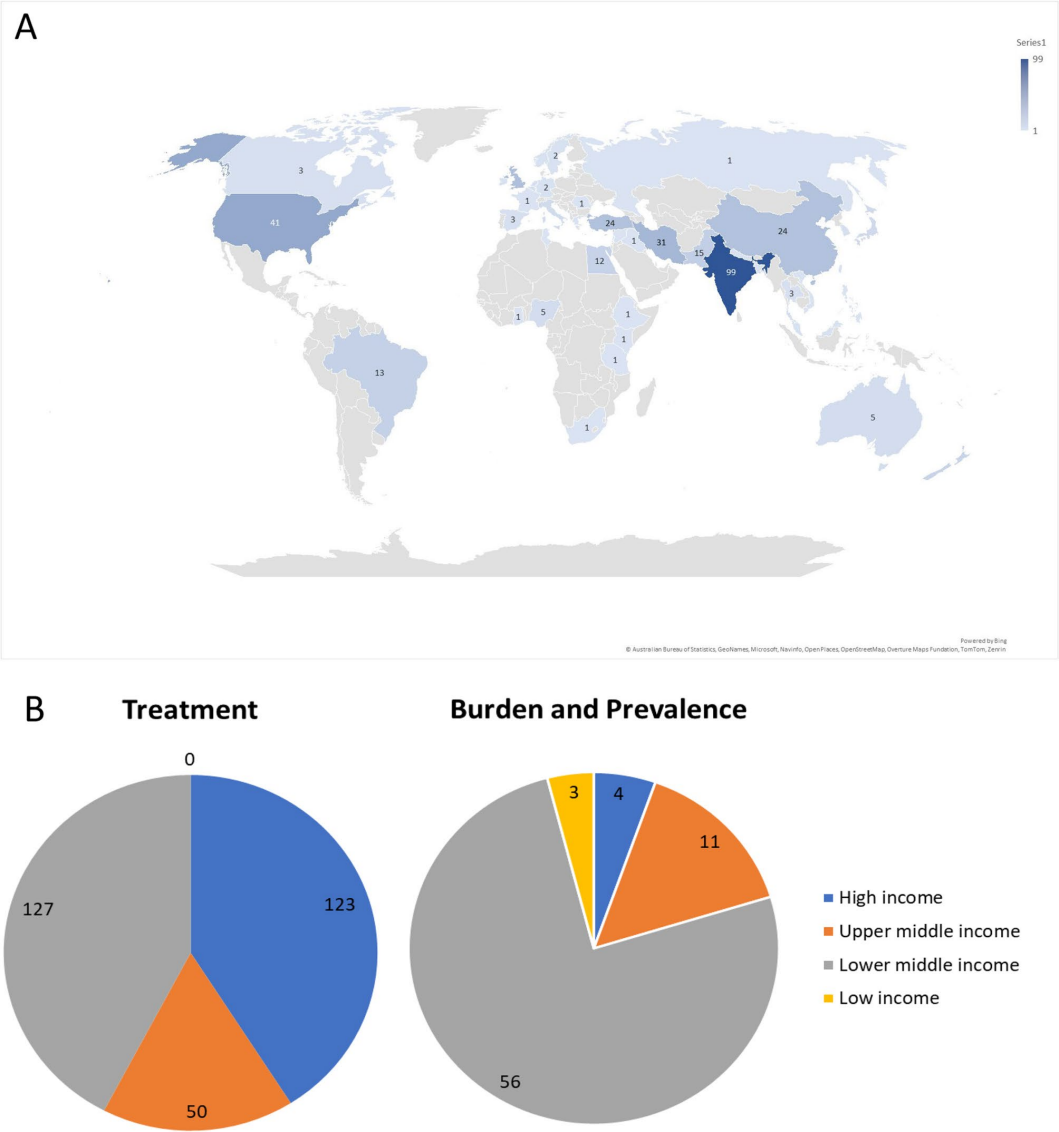
**(A)** Geographic distribution of studies reporting the treatment and prevalence & burden of HMB **(B) **Number of studies reporting the treatment, prevalence & burden of HMB by World Bank country classifications by income

The studies were conducted in 46 different countries. The largest number of studies took place in India (99 studies), followed by the United States of America (USA) (41 studies) and Iran (31 studies) (Fig. 3A). Overall, 96% of the prevalence and burden studies were carried out in LMICs (including upper middle), the majority coming from India (*n* = 32, 54%), followed by China (*n* = 5), Nigeria (*n* = 5), Brazil (*n* = 4) and Pakistan (*n* = 3) (Fig. 3B). The four burden and/or prevalence studies from high-income countries (HICs) (USA, Sweden and Ireland) were systematic reviews. In regard to data on HMB treatment, 59% of the studies were conducted in LMIC (including upper middle)s, and the geographic scope of the most commonly reported countries was as follows: India (*n* = 67), USA (*n* = 38), Iran (*n* = 29), UK (*n* = 25), Turkey (*n* = 22), China (*n* = 19), Pakistan (*n* = 12), New Zealand (*n* = 11), Egypt (*n* = 10) and Brazil (*n* = 8) (Fig. 3B).

Out of the 321 primary studies (excluding systematic reviews), 157 (48.9%) referred to HMB, 54 (16.8%) to AUB and 28 (8.7%) to dysfunctional uterine bleeding (DUB). Among the remaining studies, 38 (11.8%) reported on menstrual problems or irregularities, 24 (7.5%) on fibroids or leiomyomas and 15 (4.7%) reported on adenomyosis, all in the context of HMB. Other indications included endometrial hyperplasia (3), ovulatory disorders (1) and uterine lesions (1).

The number of study participants ranged from 10 to 130,000, the latter being a cross-sectional study based on mobile application data to investigate menstrual patterns and disorders among Chinese women of reproductive age [27]. For studies reporting on prevalence and burden, the majority (40 studies, 57.9%) included between 100 and 500 participants, 13 studies (18.8%) between 500 and 1000 participants, and 14 studies (20.3%) had more than a thousand participants. For studies reporting on treatment, the majority (181 studies, 72.4%) included between 50 and 500 participants, 49 studies (19.6%) had < 50 participants, while 20 studies (8%) included more than 500 participants (data not shown).

### Prevalence of heavy menstrual bleeding

Of the 376 included studies, 55 studies provided data regarding HMB prevalence in LMICs, four of which were systematic reviews. Many studies came from India (27 studies), followed by Nigeria (5 studies) and Brazil and China (both 3 studies). The systematic reviews were published by authors from Iran, an India and Sweden collaboration, and the USA. The number of participants in these studies ranged from 100 to 130,000, and half of the studies (27 studies) involved adolescents as the study population. Of the 51 primary research studies, the majority were cross-sectional (Fig. 2). More than half (33 studies, 64.7%) did not report how HMB was diagnosed. The remaining 18 studies used (semi) quantitative measurements, such as the number of pads and tampons used each day during the menstrual cycle (9 studies, 17.6%), subjective descriptions such as heavy, normal, light; number of days (7 studies, 13.7%), or Pictorial Blood Assessment Chart (PBAC) (2 studies, 3.9%). The reported prevalence ranged between 5% and 58%, with 24 studies (47%) reporting a prevalence between 10% and 25%. The reported prevalence did not appear to be related to the assessment method used, study size or geographic location. The HMB prevalence in the two studies using PBAC was 12.1% [28] and 18.8% [29].

### Burden of heavy menstrual bleeding

We identified 19 studies reporting on HMB burden in LMICs. This included 17 cross-sectional studies, one cohort study, and one systematic review. Study sample sizes ranged from 86 to 5609 participants. Five studies were conducted in India, followed by China and Pakistan (both 2 studies). Of note, the one systematic review on burden included in this scoping review was conducted by researchers from the USA, and while their search strategy had no country limitations, the reviews mostly report on findings from HIC. Twelve studies involved adult women with HMB, and seven involved adolescents. The consequences of HMB included QoL impairment; mental health issues such as depression, reduced physical well-being, school absenteeism, diminished sleep quality, increased economic burden on families due to healthcare costs, and health concerns e.g., anemia.

### Treatment of heavy menstrual bleeding

Over 80% of the studies (250 out of 300, excluding systematic reviews) reported treatment options for HMB. Study populations ranged from 10 to 1700 participants, five studies included both adults and adolescents, and five studies specifically included only adolescents. Thirty studies focused on herbal/traditional treatment methods, including food-related interventions (such as the intake of quince paste, ginger powder, and purslane seeds), yoga practice and dry cupping. In 27 studies, a surgical intervention (ablation *n* = 16, hysterectomy *n* = 5, resection *n* = 5, or IUD fixation surgery *n* = 1) was compared to a medical intervention, including IUD placement (24 studies), while the other three studies were medroxyprogesterone acetate, and the estradiol/levonorgestrel pill, and traditional medical treatment. One third of the treatment studies (*n* = 109, 36%) reported on one treatment for HMB, while the remaining studies compared several doses of the same treatment or compared different treatment options.

A total of 324 treatments were identified (Table 1). The use of LNG-IUD was described in 120 studies, followed by progestin (*n* = 41), COCs (*n* = 36), gonadotrophin-releasing hormone (*n* = 31), and tranexamic acid (*n* = 23). These five treatment options accounted for 78% of identified treatments. The remaining options include the selective estrogen receptor modulator ormeloxifene (*n* = 18, 5.5%), progestogen (*n* = 18, 5.5%), NSAID (*n* = 9, 2.8%), usual medical treatment (*n* = 7, 2.2%), combined vaginal ring (*n* = 4, 1.2%). Six treatments, ethamsylate, letrozole, desmopressin, ‘hormonal’ (Progesterone/Danazole/oral contraception), ‘non hormonal’ (Mefenamic acid/Tranexamic acid/Ethamsylate), calcium dobesilate, iron supplementation, and vitamin B1, were identified once or twice (*n* = 9, 2.8%). Usual medical treatment depends on the study, but typically refers to tranexamic acid, mefenamic acid, COC, or progesterone alone.

**Table 1:** Identified treatment options for heavy menstrual bleeding

Treatment options	Products	Total
Antifibrinolytic agent	Tranexamic Acid	23
Antihemorrhagic agent	Ethamsylate	2
Aromatase inhibitor	Letrozole	1
Combined oral Contraception (COC)	Estradiol/levonorgestrel (2), Estradiol Valerate/Dienogest (6), Estradiol/norethisterone acetate (2), Estradiol with nomegestrol acetate (1), Estradiol valerate/norgestrel (1), Ethinyl estradiol (1), Ethinyl estradiol with desogestrel (6), Ethinyl estradiol with levonorgestrel (10), Ethinyl estradiol with progestin + MPA (1), Ethinyl estradiol with progestin + norethindrone (1), Norgestimate ethinyl E2 Triphasic (2), Unspecified (3)	36
Estrogen receptor modulators	Ormeloxifene	18
Gonadotrophin-releasing hormone	Elagolix + add-back therapy (8), Elagolix (6), Leuprorelin (injection) (3), Linzagolix (2), Linzagolix with add-back therapy (2), Regugolix combination therapy (6) Triptorelin acetate injection (2), Unspecified (2)	31
Hormonal	Progesterone/Danazol/oral contraception	1
Iron	Iron supplement	2
Intra-uterine device (IUD)	Danazol-IUD (1), LNG-IUD (120), Multiload Cu250 intrauterine contraceptive devices (Copper IUD), releasing 0 (control), 1.5, 3, or 6 microg of 3-keto-desogestrel daily (1)	122
Non hormonal	Mefenamic acid/Tranexamic acid/Ethamsylate	1
Nonsteroidal anti-inflammatory drug	Diclofenac (1), Mefenamic acid (7), Naproxen (1)	9
Progesterone	Asoprisnil (1), Megestrol (1), Mifepristone (oral) (5), Ulipristal acetate (9), Vaginal micronized progesterone (1), Unspecified (1)	18
Progestogen	Depo-medroxyprogesterone acetate IM/medroxyprogesterone/droxyprogesterone (20), Desogestrel (1), Dienogest (oral) (1), Dydrogesterone/Didrogesterone (2), Etonogestrel (subcutaneous) (2), Norethisterone (14)	41
Prostaglandin	Misoprostol (rectal or oral)	3
Vasopressin	Desmopressin	3
Vasoprotective	Calcium dobesilate	1
Vitamine	Vitamin B1	1
Usual medical treatment	Study dependent: One study refers to usual medical treatment as: mefenamic acid, tranexamic acid, norethindrone, a combined progestogen or progesterone-only oral contraceptive pill, or medroxy­progesterone acetate injection and were chosen by the physician and patient based on contraceptive needs or the desire to avoid hormonal treatment	7
Combined Vaginal ring (CVR)		4
		**324**

Most of the studies used PBAC scores (125 studies, of which 57 studies included at least PBAC) to report on the treatment effects, followed by percentage comparisons of symptoms before and after treatment (34 studies), subjective descriptions such as ‘less heavy’ and ‘lighter’ (17 studies), number of pads used (11 studies), or other indicators [29] such as hemoglobin (Hb) level, menorrhagia multi-attribute scale (MMAS) or other questionnaires, and QoL. In 22 studies, multiple ways of reporting HMB outcomes were used, for example Hb, hematocrit levels, duration of bleeding, and MMAS. All identified treatments were reported as having positive effects for women with HMB - either a decrease in menstrual flow was observed, a higher Hb level or other related measurements, or improved QoL. When several treatment options or doses were compared, they did not differ in the overall result (reduction of HMB symptoms), but women’s satisfaction towards one of the treatments was higher and/or a difference in blood reduction was observed. Statistical analyses were not performed in numerous studies.

### Market access barriers of heavy menstrual bleeding

We did not identify studies directly reporting on market access barriers for HMB treatment options in LMICs. However, we included two studies evaluating HMB treatment barriers from the providers’ perspective. The first study, an implementation project in Kenya, identified challenges such as a lack of knowledge among caregivers, poor documentation, the absence of clear guiding protocols, limited patient awareness, and the unavailability of outpatient hysteroscopy services [30]. The second study consisted of qualitative research conducted in Brazil and found that while healthcare practitioners were aware of the significance of HMB in clinical practice, some misunderstandings persisted among Latin American obstetricians and gynecologists regarding HMB treatments [31].

## Discussion

This scoping review systematically mapped the available evidence related to the prevalence, burden, treatment, and obstacles to accessing treatment for HMB in LMICs. We identified 376 studies that reported one or more of these aspects. Most studies (300 studies, 79.8%) reported on treatment options for HMB, followed by studies reporting on prevalence (55 studies, 14.6%), studies reporting on burden (19 studies, 5.1%) and studies reporting on the barriers to HMB treatment (2 studies, 0.5%).

### Comparison to literature

The latest systematic review on menstrual disorders in developing countries dates back to 2004, including 25 studies from LMICs, and reported an estimated prevalence of HMB between 4% and 27% [32]. In a more recent study which included 4828 women from 10 LMIC cities, 48.6% of the women were classified as experiencing HMB – using the SAMANTA scale, a six-item measure of HMB for diagnosis [33]. These numbers correlate with our findings of an HMB prevalence between 5% and 57.4%. However, it must be noted that due to our broad scoping approach, the study population and eligibility criteria differ between the studies and the range of reported prevalence should be seen in that perspective. Both published studies report on the limited resources available on the prevalence of HMB in LMICs, and - when available- using various non-validated measurement tools and reporting low-quality data. We share the conclusion that most of the studies included in our scoping review did not use a validated tool to measure and report on HMB. A second relevant systematic review is the 2022 published ‘Interventions for heavy menstrual bleeding; overview of Cochrane reviews and network meta-analysis’ [12]. Though the review does not cover all treatment options as identified in this scoping review, it provides an overview of published evidence if the treatments were effective for reducing HMB and if women’s satisfaction improved [12]. With evidence coming from nine systematic reviews, the LNG-IUD is suggested to be the first choice for first-line treatment for reducing menstrual bleeding, followed by TXA, and subsequently long-cycle progestogens (medroxyprogesterone acetate, norethisterone). A Cochrane review from 2019, including 19 randomized clinical trials, concluded that NSAIDs reduce HMB when compared with placebo, but are less effective than tranexamic acid, or LNG-IUD [34].

### Clinical implications

The treatment of HMB is not a one-suits-all solution and when providing treatment options healthcare professionals should consider the woman’s preferences, any comorbidities, the presence or absence of fibroids, polyps, endometrial pathology, adenomyosis, and other symptoms such as pressure and pain [1]. The NICE guidelines (2021) for the assessment and management of HMB, recommend the placement of an LNG-IUD as the first line of treatment in women with no identified pathology, fibroids < 3 cm, or adenomyosis [1]. If an LNG-IUD is not preferred (for example, patients and providers are often uncomfortable with IUDs in adolescents), or not suitable, non-hormonal (TXA and NSAIDs) or hormonal (combined hormonal contraception and cyclical oral progestogens) are recommended. While the above recommendations cover most of the treatment options identified in this scoping review, there are other pharmaceutical treatment options such as estrogen receptor modulators (Ormeloxifene), hormone treatment (including Elagolix), or progestogen (including ulipristal acetate) available. The availability of diverse medical treatment options is promising, however it raises questions on whether healthcare workers are aware of these options and if the interventions are accessible. While we only identified two studies on HMB treatment challenges, both reported on lack of knowledge or misunderstanding regarding treatment options from a provider perspective.

### Strengths and limitations

One of the main strengths of our scoping review is the broad coverage of several aspects of HMB. By not focusing on a specific topic related to HMB, and rather investigating the available evidence on prevalence, burden, treatment options, and access barriers, we could identify the overall existing gaps in the current literature on HMB. Identification of these knowledge gaps is especially important in LMICs where resources to identify and treat HMB may be limited. Menstrual taboos persist and there may be stigmatization toward the use of hormonal methods of contraception for non-contraceptive reasons; however, our scoping review did not specifically focus on how cultural and social factors influence identification and treatment of HMB In our effort to find all relevant studies related to HMB, we broadened our search terms with AUB, DUB and general menstrual problems or irregularities, as these definitions are sometimes used interchangeably. The initial search led to the identification of 9503 unique studies, of which the majority was screened by one reviewer only. This, together with our language restriction, can be seen as a limitation, as potential studies of interest could have been missed. However, given that 1864 studies were included for retrieval and full-text review (performed by two independent reviewers) and 376 were used for data extraction, we believe our method was adequately robust to provide a comprehensive scoping overview of the available literature. As this was a broad scoping exercise, we did not evaluate the quality of the studies and recognize this as a limitation.

### Research gaps and future directions

The proportion of the studies identified in this scoping review for the different HMB aspects highlights a first research gap: there is minimal evidence on the prevalence and burden of HMB in LMICs. Only a few studies come from sub-Saharan Africa flagging that the experiences of African women are not well represented in the literature. Since the last systematic review in 2004, a few dozen prevalence studies have been published and an update of that systematic review and/or well-designed prevalence studies using objective validated measurement tool could be meaningful. Secondly, we identified no studies reporting on barriers to access of HMB treatment options. An explanation could be that these drugs are commonly used for contraception or the management of other conditions and are already widely available and accessible. Another possibility is that there are access barriers for the treatment options, though not reported in relation to the treatment of HMB and therefore not captured in our scoping review. As many diverse treatment options are available, it is worthwhile to know if women have access to them, if there are cultural barriers, lack of knowledge from the healthcare provider, or any other obstacles that can prevent treatment. Future research could further explore access barriers, including assessing how cultural context shapes reporting, diagnosis, and treatment of HMB. Lastly, we identified the lack of using validated measurements tools to identify and report on HMB. The measurements methods range from quantitative (alkaline hematin technique), to semi-quantitative visual (including PBAC) and validated QoL instruments (including SAMANTA Scale) to several types of self-reporting (quantitative such as pad count measures, non-quantitative descriptions such as “heavy” periods, or using unclear or no definitions). The lack of uniformity in HMB assessment methods introduces bias and uncertainty, undermining accurate burden estimation and the formulation of robust, evidence-based health policies. The prioritization of women – and menstrual health by donors such as the Gates Foundation [35], Pivotal Ventures [36], and the Wellcome Leap [37] underscores an important shift in the global health agenda. With dedicated funding initiatives now addressing HMB, there is an opportunity to generate robust evidence and catalyze policy and practice change.

## Conclusion

Heavy menstrual bleeding cannot be neglected as a global public health issue. It is common, causes substantial impairments in QoL, and is associated with increased need for medical and surgical interventions. The number of identified treatment options and studies in this scoping review reflects the seriousness of the condition. However, there is a need for good quality studies on the prevalence, burden, and treatment access barriers of HMB in LMICs, especially since HMB can lead to iron deficiency anemia in an already more vulnerable population.

## Supplementary Information

Supplementary Material 1.

Supplementary Material 2.

Supplementary Material 3.

## Data Availability

All data generated or analyzed during this study are included in this published article. The search strategy can be found in Supplement 2, all included studies can be found in Supplement 3.
