# Supplementary material for: The prevalence and burden of heavy menstrual bleeding, and market access barriers of medical interventions with a focus on low- and middle-income countries: a scoping review
Source: BMC Womens Health. 2025 Nov 29;26:9. doi: 10.1186/s12905-025-04157-5 (PMC12771781; doi:10.1186/s12905-025-04157-5)
Supplement: Supplementary file 2 — Supplementary Material 2. [file 12905_2025_4157_MOESM2_ESM.docx]

# Supplement File 2: Search strategies

**Database:** Ovid MEDLINE(R) and Epub Ahead of Print, In-Process, In-Data-Review & Other Non-Indexed Citations, Daily and Versions

| **#** | **Query** |
| --- | --- |
| 1 | Menorrhagia/ |
| 2 | (meno?rhagi* or menorr?agi* or menor*agi* or hypermeno?rhea* or hypermenorr?oea* or hypermeno?rh?ea* or meno?rhagy or menorr?agy or polymenor?hea* or polymenorr?ea or polymeno?rh?ea or menometro?rhagi* or menometrorr?agi* or menometror*agi? or Menstrua* morbidity).mp. |
| 3 | (((severe or severity or massive* or excessive* or extensive* or large scale or acute or unrelenting or overwhelming or uncontrolled or extreme or significant or heavy or heavier or debilitat* or prolonged or excess or dysfunctional or abnormal or abundant or profuse) adj3 (bleed* or blood loss* or blood flow* or h?em?or*ag*)) and (menstrua* or menses or catamenia or menstruum)).mp. |
| 4 | ((heavy or massive* or excessive* or extensive* or debilitat* or prolonged or dysfunctional or abnormal or severe) adj (menstrua* or menses or catamenia or menstruum)).mp. |
| 5 | ((heavy adj (period? or MBL)) or (HMB adj2 (AUB or MBL or DUB))).mp. |
| 6 | ((long* duration or excessive duration or prolonged duration or excessively long or prolonged period* or heavy flow or excessive flow or prolonged flow or abnormal flow or severe flow) adj5 (menstrua* or menses or catamenia or menstruum)).mp. |
| 7 | ((suffer* or disabl* or debilitat* or burden* or impact*) adj3 HMB).mp. |
| 8 | ((bleeding or blood loss) adj3 (score? or pattern? or measur* or assess* or amount? or evaluat*) adj3 (menstrua* or menses or catamenia or menstruum or MBL)).mp. |
| 9 | ((abnormal or excessive) adj (uterine bleeding or uterine blood loss)).mp. |
| 10 | ((ovulatory disorder* or adenomyosis or endometrios* or endometrial polyp? or endometrial hyperplasia or uterine fibroid? or leiomyomata) and ((severe or severity or massive* or excessive* or extensive* or large scale or acute or unrelenting or overwhelming or uncontrolled or extreme or significant or heavy or heavier or debilitat* or prolonged or excess or dysfunctional or abnormal or abundant or profuse) adj3 (bleed* or blood loss* or blood flow* or h?em?or*ag*))).mp. |
| 11 | or/1-10 |
| 12 | "quality of life"/ or psychological well-being/ or disability-adjusted life years/ or quality-adjusted life years/ or (QOL or HRQOL or DALY or QALY or quality of life or life quality or experience* or burden*).mp. |
| 13 | personal autonomy/ or Personal Satisfaction/ or Consumer Behavior/ or Patient Satisfaction/ or self concept/ or self-assessment/ or self disclosure/ or self efficacy/ or self-compassion/ or attitude/ or attitude to health/ or health knowledge, attitudes, practice/ or patient preference/ |
| 14 | ("living day to day" or "lives of" or "living with" or normal life or normal lives or ((every day or everyday or daily) adj (life or living or routine* or functioning or activit* or interest?)) or ((realities or difficulties) adj2 (life or living)) or (life quality or life satisfaction or "satisf* with life") or (school life or work life or social life or family life or future life or future lives or life activit* or psychosocial health or mental health or wellbeing or well-being or emotional function* or school functioning)).mp. |
| 15 | (self concept or self belief or self image or self advoca* or self aware* or self help or self esteem or "sense of self" or self worth or self achiev* or self confidence or self regard).mp. |
| 16 | ((impact* or affect* or difficulties or problems or dysfunction* or disrupt* or disturb* or implication* or implications or concerns or worries or worrying or adjustment* or interfer* or imping* or limited or limiting) adj3 (physical or social or emotional or psychosocial or disabilit* or disabl* or abilit* or life* or living or lives or functioning or activit*)).mp. |
| 17 | ((impact* or interfere* or affect* or difficulties or problems or dysfunction* or disordered or disrupt* or disturb* or implications or concerns or worries or worrying or adjustment or interfer* or imping*) adj3 (studying or school or training or learning or employment or work or job or jobs or performance or studies or occupation* or responsibilit* or roles or future*)).mp. |
| 18 | or/12-17 |
| 19 | (cultural* or transcultural or ethnic* or racial* or stigma*).mp. |
| 20 | (access* or need* or seek* or accept* or prefer* or trust* or encounter* or understand or satisfaction or dissatisfaction or acceptable or perceive* or perception* or expectation* or wait* time* or wait* list* or timeliness or delay* or price* or pricing or fund* or availability or available or cost* or monetary or budget* or financ* or reimburs* or payer* or payee or investment* or access or accessibility or accessible* or supply or market* or affordability or affordable* or quality or pre-qualified or off-label or inequit* or inequalit* or disbursement? or expenditure or expense? or expensive* or remittance or recompens* or repayment? or refund* or recoup* or rebate? or compensate? or compensation or prepay* or overpay* or underpay*).mp. |
| 21 | (Barrier* or facilitat* or enable* or enabling or challenge* or attitude* or belief* or views* or behavio?r* or influenc* or adopt* or uptake or eligibility or eligible or value* or economic* or discount* or payment* or provision* or choice? or "willingness to pay" or repurposed or universal health coverage or stringent regulatory authorit* or national regulatory authorit* or procurement or registration or infrastructure or remuneration or safety or efficacy).mp. |
| 22 | ((restrict* or limit*) and (distribut* or prescri* or dispens*)).mp. |
| 23 | ((counterfeit or fake or false or falsified) adj (drug* or medicine* or medication*)).mp. |
| 24 | or/19-23 |
| 25 | Developing Countries/ or africa/ or africa, northern/ or algeria/ or egypt/ or libya/ or morocco/ or tunisia/ or "africa south of the sahara"/ or africa, central/ or cameroon/ or central african republic/ or chad/ or congo/ or "democratic republic of the congo"/ or equatorial guinea/ or gabon/ or "sao tome and principe"/ or africa, eastern/ or burundi/ or djibouti/ or eritrea/ or ethiopia/ or kenya/ or rwanda/ or somalia/ or south sudan/ or sudan/ or tanzania/ or uganda/ or africa, southern/ or angola/ or botswana/ or eswatini/ or lesotho/ or malawi/ or mozambique/ or namibia/ or south africa/ or zambia/ or zimbabwe/ or africa, western/ or benin/ or burkina faso/ or cabo verde/ or cote d'ivoire/ or gambia/ or ghana/ or guinea/ or guinea-bissau/ or liberia/ or mali/ or mauritania/ or niger/ or nigeria/ or senegal/ or sierra leone/ or togo/ or caribbean region/ or west indies/ or cuba/ or dominica/ or dominican republic/ or grenada/ or guadeloupe/ or haiti/ or jamaica/ or saint lucia/ or "saint vincent and the grenadines"/ or central america/ or belize/ or costa rica/ or el salvador/ or guatemala/ or honduras/ or nicaragua/ or panama/ or latin america/ or mexico/ or south america/ or argentina/ or bolivia/ or brazil/ or colombia/ or ecuador/ or french guiana/ or guyana/ or paraguay/ or peru/ or suriname/ or venezuela/ or asia/ or asia, central/ or kazakhstan/ or kyrgyzstan/ or tajikistan/ or turkmenistan/ or uzbekistan/ or asia, southeastern/ or borneo/ or cambodia/ or indonesia/ or laos/ or malaysia/ or myanmar/ or philippines/ or thailand/ or timor-leste/ or vietnam/ or asia, western/ or bangladesh/ or bhutan/ or india/ or sikkim/ or afghanistan/ or iran/ or iraq/ or jordan/ or lebanon/ or syria/ or turkey/ or yemen/ or nepal/ or pakistan/ or sri lanka/ or china/ or korea/ or "democratic people's republic of korea"/ or mongolia/ or "sao tome and principe"/ or indian ocean islands/ or comoros/ or madagascar/ or mauritius/ or pacific islands/ or melanesia/ or fiji/ or papua new guinea/ or vanuatu/ or micronesia/ or palau/ or samoa/ or american samoa/ or "independent state of samoa"/ or tonga/ or romania/ or russia/ or croatia/ or albania/ or "bosnia and herzegovina"/ or bulgaria/ or kosovo/ or "republic of north macedonia"/ or moldova/ or montenegro/ or "republic of belarus"/ or serbia/ or ukraine/ or armenia/ or azerbaijan/ or "georgia (republic)"/ |
| 26 | (Afghanistan* or Albania* or Algeria* or Angola* or Argentina* or Armenia* or Azerbaijan* or Bangladesh* or Beliz* or Benin* or Bhutan* or Bolivia* or Bosnia* or Herzegovin* or Botswan* or Brazil* or Bulgaria* or Burkina* or Burundi* or Cabo Verde* or Cape Verde* or Cambodia* or Cameroon* or Chad* or China or Chinese or Colombia* or Comor* or Congo* or Costa Rica* or Cote d'Ivoir* or Ivory Coast or Cuba* or Djibouti* or Dominica* or Ecuador* or Egypt* or El Salvador* or Eritrea* or Ethiopia* or Fiji* or Gabon* or Gambia* or Georgia* or Ghana* or Grenad* or Guatemala* or Guinea* or Guyan* or Haiti* or Hondura* or Hungar* or India* or Indonesia* or Iran* or Iraq* or Jamaica* or Jordan* or Kazakhstan* or Kenya* or Kiribati* or Korea* or Kosov* or Kyrgyz* or Lao* or Leban* or Lesotho* or Liberia* or Libya* or Macedonia* or Madagascar* or Malawi* or Malaysia* or Maldiv* or Mali* or Marshall Island* or Mauritania* or Mauriti* or Mexic* or Moldova* or Mongolia* or Montenegr* or Morocc* or Mozambi* or Myanma* or Burmese or Namibia* or Nepal* or Nicaragua* or Niger* or Pakistan* or Palau* or Panama* or Papua New Guinea* or Paraguay* or Peru* or Philippines or Filipino or Romania* or Rwanda* or Samoa* or Sao Tome* or Senegal* or Serbia* or Seychell* or Sierra Leon* or Solomon Island* or Somalia* or Sudan* or Sri Lanka* or St Lucia* or Saint Lucia or St Vincent or Saint Vincent or Grenadines or Surinam* or Swazi* or Syria* or Tajikistan* or Tanzania* or Thai* or Timor* or Togo* or Tonga* or Tunisia* or Turk* or Tuvalu* or Uganda* or Ukrain* or Uzbekistan* or Vanuatu* or Venezuela* or Vietnam* or Viet-Nam* or West Bank or Gaza or Yemen* or Zambia* or Zimbabwe* or Russia* or Croatia* or Nauru* or Yugoslavia* or USSR or Soviet* or Byelarus* or Belarus* or (africa* or asia* or caribbean or central america* or latin america* or south america* or melanesia* or micronesia* or polynesia*)).mp. |
| 27 | (resource-limit* or resource-poor or low-resource* or limited-resource* or resource-constrain* or constrain*-resource* or under-resource* or poor*-resource* or resource-scarce* or scarce*-resource* or low-income or middle-income or lowincome or middleincome or (low* adj3 middle-income)).mp. |
| 28 | ((developing or underdeveloped or under-developed or emerging or less-developed or least-developed or less-economically developed or least-economically developed or less-affluent or least-affluent or least-industriali#ed or non-industriali#ed or deprived or poor) adj (country or countries or nation? or region? or economy or economies)).mp. |
| 29 | ((developing or underdeveloped or under-developed or less-developed or least-developed) adj (population* or world)).mp. |
| 30 | (third-world* or thirdworld* or 3rd-world* or lmic or lmics or lami countr* or lalmi countr* or transitional countr*).mp. |
| 31 | (low* adj (gdp or gnp or gross domestic or gross national)).mp. |
| 32 | ((underserved or under-served) adj (countr* or nation? or population*)).mp. |
| 33 | or/25-32 |
| 34 | 11 and 18 and 33 |
| 35 | 11 and 24 and 33 |
| 36 | exp anti-inflammatory agents, non-steroidal/ or exp cyclooxygenase inhibitors/ or exp cyclooxygenase 2 inhibitors/ or Tranexamic Acid/ or exp Contraceptives, Oral, Hormonal/ or exp Contraceptives, Oral, Combined/ or Intrauterine Devices, Medicated/ or exp Progestins/ |
| 37 | (non-steroid* antiinflammatory or non-steroid* anti-inflammatory or nonsteroid antiinflammatory or nonsteroid* anti-inflammatory or NSAID or NSAIDs or (cyclooxygenase inhibitor* or cyclo-oxygenase inhibitor* or prostaglandin synthetase inhibitor* or cyclooxygenase-2 inhibitor* or COX-2 inhibitor* or COX-2 specific inhibitor* or COX2 inhibitor* or COX2 specific inhibitor* or coxib or coxibs)).mp. |
| 38 | (antifibrinolytic* or fibrinolysis inhibitor* or aminomethyl cyclohexane carboxylic acid* or aminomethyl cyclohexanecarboxylic acid* or aminomethylcyclohexane carbonic acid* or aminomethylcyclohexane carboxylic acid* or aminomethylcyclohexanecarbonic acid* or aminomethylcyclohexanecarboxylic acid* or aminomethylcyclohexanocarboxylic acid* or aminomethylcyclohexanoic acid* or tranexamic acid* or tranexam or tranexanic acid* or transexamic acid* or traxamic or TXA).mp. |
| 39 | (combined oral contraceptive* or combined hormonal contraceptive* or combined oral hormonal contraceptive* or combined vaginal ring* or combined hormonal vaginal ring* or contraceptive vaginal ring* or (luteal oral progestogen* or LNG-IUS or LNG-IUD or levonorgestrel IUD* or levonorgestrel releasing intrauterine or levonorgestrel releasing IUD or levonorgestrel releasing intra-uterine or levonorgestrel intrauterine system or levonorgestrel intrauterine device) or (gestogen* or progestagen* or progestin* or progestogen*)).mp. |
| 40 | or/36-39 |
| 41 | 11 and 40 |
| 42 | 34 or 35 or 41 |
| 43 | limit 42 to yr="2000 -Current" |
| 44 | exp animals/ not humans.sh. |
| 45 | 43 not 44 |
| 46 | limit 45 to (comment or editorial or letter) |
| **47** | **45 not 46** |

**New records since June 2023**

| 48 | (202306* or 202307* or 202308* or 202309* or 202310* or 202311* or 202312* or 202401* or 202402* or 202403* or 202404* or 202405* or 202406* or 202407* or 202408* or 202409* or 202410*).dt,ez,dp,ed. |
| --- | --- |
| 49 | 47 and 48 |
| 50 | limit 47 to yr="2024 -Current" |
| **51** | **49 or 50** |

**Database:** Embase

| **#** | **Query** |
| --- | --- |
| 1 | menorrhagia/ or menometrorrhagia/ |
| 2 | (meno?rhagi* or menorr?agi* or menor*agi* or hypermeno?rhea* or hypermenorr?oea* or hypermeno?rh?ea* or meno?rhagy or menorr?agy or polymenor?hea* or polymenorr?ea or polymeno?rh?ea or menometro?rhagi* or menometrorr?agi* or menometror*agi? or Menstrua* morbidity).mp. |
| 3 | (((severe or severity or massive* or excessive* or extensive* or large scale or acute or unrelenting or overwhelming or uncontrolled or extreme or significant or heavy or heavier or debilitat* or prolonged or excess or dysfunctional or abnormal or abundant or profuse) adj3 (bleed* or blood loss* or blood flow* or h?em?or*ag*)) and (menstrua* or menses or catamenia or menstruum)).mp. |
| 4 | ((heavy or massive* or excessive* or extensive* or debilitat* or prolonged or dysfunctional or abnormal or severe) adj (menstrua* or menses or catamenia or menstruum)).mp. |
| 5 | ((heavy adj (period? or MBL)) or (HMB adj2 (AUB or MBL or DUB))).mp. |
| 6 | ((long* duration or excessive duration or prolonged duration or excessively long or prolonged period* or heavy flow or excessive flow or prolonged flow or abnormal flow or severe flow) adj5 (menstrua* or menses or catamenia or menstruum)).mp. |
| 7 | ((suffer* or disabl* or debilitat* or burden* or impact*) adj3 HMB).mp. |
| 8 | ((bleeding or blood loss) adj3 (score? or pattern? or measur* or assess* or amount? or evaluat*) adj3 (menstrua* or menses or catamenia or menstruum or MBL)).mp. |
| 9 | ((abnormal or excessive) adj (uterine bleeding or uterine blood loss)).mp. |
| 10 | ((ovulatory disorder* or adenomyosis or endometrios* or endometrial polyp? or endometrial hyperplasia or uterine fibroid? or leiomyomata) and ((severe or severity or massive* or excessive* or extensive* or large scale or acute or unrelenting or overwhelming or uncontrolled or extreme or significant or heavy or heavier or debilitat* or prolonged or excess or dysfunctional or abnormal or abundant or profuse) adj3 (bleed* or blood loss* or blood flow* or h?em?or*ag*))).mp. |
| 11 | or/1-10 |
| 12 | "quality of life"/ or psychological well-being/ or disability-adjusted life year/ or quality adjusted life year/ or (QOL or HRQOL or DALY or QALY or quality of life or life quality or experience* or burden*).mp. |
| 13 | personal autonomy/ or satisfaction/ or life satisfaction/ or patient satisfaction/ or consumer attitude/ or self concept/ or self compassion/ or self disclosure/ or self evaluation/ or attitude to health/ or attitude/ or health behavior/ or knowledge/ or patient preference/ or patient attitude/ |
| 14 | ("living day to day" or "lives of" or "living with" or normal life or normal lives or ((every day or everyday or daily) adj (life or living or routine* or functioning or activit* or interest?)) or ((realities or difficulties) adj2 (life or living)) or (life quality or life satisfaction or "satisf* with life") or (school life or work life or social life or family life or future life or future lives or life activit* or psychosocial health or mental health or wellbeing or well-being or emotional function* or school functioning)).mp. |
| 15 | (self concept or self belief or self image or self advoca* or self aware* or self help or self esteem or "sense of self" or self worth or self achiev* or self confidence or self regard).mp. |
| 16 | ((impact* or affect* or difficulties or problems or dysfunction* or disrupt* or disturb* or implication* or implications or concerns or worries or worrying or adjustment* or interfer* or imping* or limited or limiting) adj3 (physical or social or emotional or psychosocial or disabilit* or disabl* or abilit* or life* or living or lives or functioning or activit*)).mp. |
| 17 | ((impact* or interfere* or affect* or difficulties or problems or dysfunction* or disordered or disrupt* or disturb* or implications or concerns or worries or worrying or adjustment or interfer* or imping*) adj3 (studying or school or training or learning or employment or work or job or jobs or performance or studies or occupation* or responsibilit* or roles or future*)).mp. |
| 18 | or/12-17 |
| 19 | (cultural* or transcultural or ethnic* or racial* or stigma*).mp. |
| 20 | (access* or need* or seek* or accept* or prefer* or trust* or encounter* or understand or satisfaction or dissatisfaction or acceptable or perceive* or perception* or expectation* or wait* time* or wait* list* or timeliness or delay* or price* or pricing or fund* or availability or available or cost* or monetary or budget* or financ* or reimburs* or payer* or payee or investment* or access or accessibility or accessible* or supply or market* or affordability or affordable* or quality or pre-qualified or off-label or inequit* or inequalit* or disbursement? or expenditure or expense? or expensive* or remittance or recompens* or repayment? or refund* or recoup* or rebate? or compensate? or compensation or prepay* or overpay* or underpay*).mp. |
| 21 | (Barrier* or facilitat* or enable* or enabling or challenge* or attitude* or belief* or views* or behavio?r* or influenc* or adopt* or uptake or eligibility or eligible or value* or economic* or discount* or payment* or provision* or choice? or "willingness to pay" or repurposed or universal health coverage or stringent regulatory authorit* or national regulatory authorit* or procurement or registration or infrastructure or remuneration or safety or efficacy).mp. |
| 22 | ((restrict* or limit*) and (distribut* or prescri* or dispens*)).mp. |
| 23 | ((counterfeit or fake or false or falsified) adj (drug* or medicine* or medication*)).mp. |
| 24 | or/19-23 |
| 25 | developing country/ or low income country/ or middle income country/ or Africa/ or Africa south of the Sahara/ or North Africa/ or angola/ or benin/ or Botswana/ or Burkina Faso/ or Burundi/ or Cameroon/ or Cape Verde/ or Central Africa/ or Central African Republic/ or Chad/ or Comoros/ or Congo/ or Cote d'Ivoire/ or Democratic Republic Congo/ or Djibouti/ or Equatorial Guinea/ or Eritrea/ or Eswatini/ or Ethiopia/ or Gabon/ or Gambia/ or Ghana/ or Guinea/ or Guinea-Bissau/ or Kenya/ or Lesotho/ or Liberia/ or Madagascar/ or Malawi/ or Mali/ or Mozambique/ or Namibia/ or Niger/ or Nigeria/ or Rwanda/ or Sahel/ or Senegal/ or Sierra Leone/ or Somalia/ or South Africa/ or South Sudan/ or Sudan/ or Tanzania/ or Togo/ or Uganda/ or Zambia/ or Zimbabwe/ or Algeria/ or Egypt/ or Libyan Arab Jamahiriya/ or Mauritania/ or Morocco/ or Tunisia/ or Western Sahara/ or Central Africa/ or North Africa/ or African Caribbean/ or Caribbean/ or Central America/ or "South and Central America"/ or Belize/ or Costa Rica/ or El Salvador/ or Guatemala/ or Honduras/ or Nicaragua/ or Panama/ or Antillean/ or Caribbean Islands/ or Cuba/ or Dominica/ or Dominican Republic/ or Grenada/ or Guadeloupe/ or Jamaica/ or Haiti/ or Martinique/ or Saint Lucia/ or "Saint Vincent and the Grenadines"/ or "caribbean (person)"/ or Cuban/ or "dominican (dominica)"/ or "dominican (dominican republic)"/ or Haitian/ or Jamaican/ or South America/ or Argentina/ or Bolivia/ or Brazil/ or Colombia/ or Ecuador/ or French Guiana/ or Guyana/ or Paraguay/ or Peru/ or Suriname/ or Venezuela/ or Mexico/ or Asia/ or central Asia/ or Far East/ or Middle East/ or northern Asia/ or South Asia/ or western Asia/ or Kazakhstan/ or Kyrgyzstan/ or Tajikistan/ or Turkmenistan/ or Uzbekistan/ or China/ or Korea/ or Mongolia/ or Philippines/ or Southeast Asia/ or North Korea/ or Borneo/ or Cambodia/ or Indonesia/ or Laos/ or Malaysia/ or Myanmar/ or Papua New Guinea/ or Singapore/ or Thailand/ or Timor-Leste/ or Viet Nam/ or Iran/ or Iraq/ or Jordan/ or Lebanon/ or Palestine/ or Syrian Arab Republic/ or "turkey (republic)"/ or Yemen/ or Afghanistan/ or Bangladesh/ or Bhutan/ or India/ or Nepal/ or Pakistan/ or Sri Lanka/ or Armenia/ or Azerbaijan/ or "georgia (republic)"/ or "Sao Tome and Principe"/ or Mauritius/ or Pacific Islands/ or Federated States of Micronesia/ or Fiji/ or Kiribati/ or Marshall Islands/ or Melanesia/ or Nauru/ or Palau/ or Polynesia/ or Samoan Islands/ or Solomon Islands/ or Timor-Leste/ or Tonga/ or Tuvalu/ or Vanuatu/ or American Samoa/ or Samoa/ or Romania/ or Russian Federation/ or USSR/ or Croatia/ or Albania/ or Belarus/ or "Bosnia and Herzegovina"/ or Bulgaria/ or Kosovo/ or Moldova/ or "Montenegro (republic)"/ or Republic of North Macedonia/ or Serbia/ or Ukraine/ or "Federation of Bosnia and Herzegovina"/ |
| 26 | (Afghanistan* or Albania* or Algeria* or Angola* or Argentina* or Armenia* or Azerbaijan* or Bangladesh* or Beliz* or Benin* or Bhutan* or Bolivia* or Bosnia* or Herzegovin* or Botswan* or Brazil* or Bulgaria* or Burkina* or Burundi* or Cabo Verde* or Cape Verde* or Cambodia* or Cameroon* or Chad* or China or Chinese or Colombia* or Comor* or Congo* or Costa Rica* or Cote d'Ivoir* or Ivory Coast or Cuba* or Djibouti* or Dominica* or Ecuador* or Egypt* or El Salvador* or Eritrea* or Ethiopia* or Fiji* or Gabon* or Gambia* or Georgia* or Ghana* or Grenad* or Guatemala* or Guinea* or Guyan* or Haiti* or Hondura* or Hungar* or India* or Indonesia* or Iran* or Iraq* or Jamaica* or Jordan* or Kazakhstan* or Kenya* or Kiribati* or Korea* or Kosov* or Kyrgyz* or Lao* or Leban* or Lesotho* or Liberia* or Libya* or Macedonia* or Madagascar* or Malawi* or Malaysia* or Maldiv* or Mali* or Marshall Island* or Mauritania* or Mauriti* or Mexic* or Moldova* or Mongolia* or Montenegr* or Morocc* or Mozambi* or Myanma* or Burmese or Namibia* or Nepal* or Nicaragua* or Niger* or Pakistan* or Palau* or Panama* or Papua New Guinea* or Paraguay* or Peru* or Philippines or Filipino or Romania* or Rwanda* or Samoa* or Sao Tome* or Senegal* or Serbia* or Seychell* or Sierra Leon* or Solomon Island* or Somalia* or Sudan* or Sri Lanka* or St Lucia* or Saint Lucia or St Vincent or Saint Vincent or Grenadines or Surinam* or Swazi* or Syria* or Tajikistan* or Tanzania* or Thai* or Timor* or Togo* or Tonga* or Tunisia* or Turk* or Tuvalu* or Uganda* or Ukrain* or Uzbekistan* or Vanuatu* or Venezuela* or Vietnam* or Viet-Nam* or West Bank or Gaza or Yemen* or Zambia* or Zimbabwe* or Russia* or Croatia* or Nauru* or Yugoslavia* or USSR or Soviet* or Byelarus* or Belarus* or (africa* or asia* or caribbean or central america* or latin america* or south america* or melanesia* or micronesia* or polynesia*)).mp. |
| 27 | (resource-limit* or resource-poor or low-resource* or limited-resource* or resource-constrain* or constrain*-resource* or under-resource* or poor*-resource* or resource-scarce* or scarce*-resource* or low-income or middle-income or lowincome or middleincome or (low* adj3 middle-income)).mp. |
| 28 | ((developing or underdeveloped or under-developed or emerging or less-developed or least-developed or less-economically developed or least-economically developed or less-affluent or least-affluent or least-industriali#ed or non-industriali#ed or deprived or poor) adj (country or countries or nation? or region? or economy or economies)).mp. |
| 29 | ((developing or underdeveloped or under-developed or less-developed or least-developed) adj (population* or world)).mp. |
| 30 | (third-world* or thirdworld* or 3rd-world* or lmic or lmics or lami countr* or lalmi countr* or transitional countr*).mp. |
| 31 | (low* adj (gdp or gnp or gross domestic or gross national)).mp. |
| 32 | ((underserved or under-served) adj (countr* or nation? or population*)).mp. |
| 33 | or/25-32 |
| 34 | 11 and 18 and 33 |
| 35 | 11 and 24 and 33 |
| 36 | exp nonsteroid antiinflammatory agent/ or exp prostaglandin synthase inhibitor/ or exp cyclooxygenase 2 inhibitor/ or tranexamic acid/ or exp oral contraceptive agent/ or intrauterine contraceptive device/ or levonorgestrel releasing intrauterine system/ or exp gestagen/ |
| 37 | (non-steroid* antiinflammatory or non-steroid* anti-inflammatory or nonsteroid antiinflammatory or nonsteroid* anti-inflammatory or NSAID or NSAIDs or (cyclooxygenase inhibitor* or cyclo-oxygenase inhibitor* or prostaglandin synthetase inhibitor* or cyclooxygenase-2 inhibitor* or COX-2 inhibitor* or COX-2 specific inhibitor* or COX2 inhibitor* or COX2 specific inhibitor* or coxib or coxibs)).mp. |
| 38 | (antifibrinolytic* or fibrinolysis inhibitor* or aminomethyl cyclohexane carboxylic acid* or aminomethyl cyclohexanecarboxylic acid* or aminomethylcyclohexane carbonic acid* or aminomethylcyclohexane carboxylic acid* or aminomethylcyclohexanecarbonic acid* or aminomethylcyclohexanecarboxylic acid* or aminomethylcyclohexanocarboxylic acid* or aminomethylcyclohexanoic acid* or tranexamic acid* or tranexam or tranexanic acid* or transexamic acid* or traxamic or TXA).mp. |
| 39 | (combined oral contraceptive* or combined hormonal contraceptive* or combined oral hormonal contraceptive* or combined vaginal ring* or combined hormonal vaginal ring* or contraceptive vaginal ring* or (luteal oral progestogen* or LNG-IUS or LNG-IUD or levonorgestrel IUD* or levonorgestrel releasing intrauterine or levonorgestrel releasing IUD or levonorgestrel releasing intra-uterine or levonorgestrel intrauterine system or levonorgestrel intrauterine device) or (gestogen* or progestagen* or progestin* or progestogen*)).mp. |
| 40 | or/36-39 |
| 41 | 11 and 40 |
| 42 | 34 or 35 or 41 |
| 43 | limit 42 to yr="2000 -Current" |
| 44 | (exp animal/ or nonhuman/ or exp invertebrate/ or animal.hw.) not exp human/ |
| 45 | 43 not 44 |
| 46 | limit 45 to (editorial or letter or conference abstract) |
| **47** | **45 not 46** |

**New records since June 2023**

| 48 | (202306* or 202307* or 202308* or 202309* or 202310* or 202311* or 202312* or 202401* or 202402* or 202403* or 202404* or 202405* or 202406* or 202407* or 202408* or 202409* or 202410*).dc,dp,yr. |
| --- | --- |
| 49 | 47 and 48 |
| 50 | limit 47 to yr="2024 -Current" |
| **51** | **49 or 50** |

**Database:** Ovid Emcare

| **#** | **Query** |
| --- | --- |
| 1 | menorrhagia/ or menometrorrhagia/ |
| 2 | (meno?rhagi* or menorr?agi* or menor*agi* or hypermeno?rhea* or hypermenorr?oea* or hypermeno?rh?ea* or meno?rhagy or menorr?agy or polymenor?hea* or polymenorr?ea or polymeno?rh?ea or menometro?rhagi* or menometrorr?agi* or menometror*agi? or Menstrua* morbidity).mp. |
| 3 | (((severe or severity or massive* or excessive* or extensive* or large scale or acute or unrelenting or overwhelming or uncontrolled or extreme or significant or heavy or heavier or debilitat* or prolonged or excess or dysfunctional or abnormal or abundant or profuse) adj3 (bleed* or blood loss* or blood flow* or h?em?or*ag*)) and (menstrua* or menses or catamenia or menstruum)).mp. |
| 4 | ((heavy or massive* or excessive* or extensive* or debilitat* or prolonged or dysfunctional or abnormal or severe) adj (menstrua* or menses or catamenia or menstruum)).mp. |
| 5 | ((heavy adj (period? or MBL)) or (HMB adj2 (AUB or MBL or DUB))).mp. |
| 6 | ((long* duration or excessive duration or prolonged duration or excessively long or prolonged period* or heavy flow or excessive flow or prolonged flow or abnormal flow or severe flow) adj5 (menstrua* or menses or catamenia or menstruum)).mp. |
| 7 | ((suffer* or disabl* or debilitat* or burden* or impact*) adj3 HMB).mp. |
| 8 | ((bleeding or blood loss) adj3 (score? or pattern? or measur* or assess* or amount? or evaluat*) adj3 (menstrua* or menses or catamenia or menstruum or MBL)).mp. |
| 9 | ((abnormal or excessive) adj (uterine bleeding or uterine blood loss)).mp. |
| 10 | ((ovulatory disorder* or adenomyosis or endometrios* or endometrial polyp? or endometrial hyperplasia or uterine fibroid? or leiomyomata) and ((severe or severity or massive* or excessive* or extensive* or large scale or acute or unrelenting or overwhelming or uncontrolled or extreme or significant or heavy or heavier or debilitat* or prolonged or excess or dysfunctional or abnormal or abundant or profuse) adj3 (bleed* or blood loss* or blood flow* or h?em?or*ag*))).mp. |
| 11 | or/1-10 |
| 12 | "quality of life"/ or psychological well-being/ or disability-adjusted life year/ or quality adjusted life year/ or (QOL or HRQOL or DALY or QALY or quality of life or life quality or experience* or burden*).mp. |
| 13 | personal autonomy/ or satisfaction/ or life satisfaction/ or patient satisfaction/ or consumer attitude/ or self concept/ or self compassion/ or self disclosure/ or self evaluation/ or attitude to health/ or attitude/ or health behavior/ or knowledge/ or patient preference/ or patient attitude/ |
| 14 | ("living day to day" or "lives of" or "living with" or normal life or normal lives or ((every day or everyday or daily) adj (life or living or routine* or functioning or activit* or interest?)) or ((realities or difficulties) adj2 (life or living)) or (life quality or life satisfaction or "satisf* with life") or (school life or work life or social life or family life or future life or future lives or life activit* or psychosocial health or mental health or wellbeing or well-being or emotional function* or school functioning)).mp. |
| 15 | (self concept or self belief or self image or self advoca* or self aware* or self help or self esteem or "sense of self" or self worth or self achiev* or self confidence or self regard).mp. |
| 16 | ((impact* or affect* or difficulties or problems or dysfunction* or disrupt* or disturb* or implication* or implications or concerns or worries or worrying or adjustment* or interfer* or imping* or limited or limiting) adj3 (physical or social or emotional or psychosocial or disabilit* or disabl* or abilit* or life* or living or lives or functioning or activit*)).mp. |
| 17 | ((impact* or interfere* or affect* or difficulties or problems or dysfunction* or disordered or disrupt* or disturb* or implications or concerns or worries or worrying or adjustment or interfer* or imping*) adj3 (studying or school or training or learning or employment or work or job or jobs or performance or studies or occupation* or responsibilit* or roles or future*)).mp. |
| 18 | or/12-17 |
| 19 | (cultural* or transcultural or ethnic* or racial* or stigma*).mp. |
| 20 | (access* or need* or seek* or accept* or prefer* or trust* or encounter* or understand or satisfaction or dissatisfaction or acceptable or perceive* or perception* or expectation* or wait* time* or wait* list* or timeliness or delay* or price* or pricing or fund* or availability or available or cost* or monetary or budget* or financ* or reimburs* or payer* or payee or investment* or access or accessibility or accessible* or supply or market* or affordability or affordable* or quality or pre-qualified or off-label or inequit* or inequalit* or disbursement? or expenditure or expense? or expensive* or remittance or recompens* or repayment? or refund* or recoup* or rebate? or compensate? or compensation or prepay* or overpay* or underpay*).mp. |
| 21 | (Barrier* or facilitat* or enable* or enabling or challenge* or attitude* or belief* or views* or behavio?r* or influenc* or adopt* or uptake or eligibility or eligible or value* or economic* or discount* or payment* or provision* or choice? or "willingness to pay" or repurposed or universal health coverage or stringent regulatory authorit* or national regulatory authorit* or procurement or registration or infrastructure or remuneration or safety or efficacy).mp. |
| 22 | ((restrict* or limit*) and (distribut* or prescri* or dispens*)).mp. |
| 23 | ((counterfeit or fake or false or falsified) adj (drug* or medicine* or medication*)).mp. |
| 24 | or/19-23 |
| 25 | developing country/ or low income country/ or middle income country/ or Africa/ or Africa south of the Sahara/ or North Africa/ or angola/ or benin/ or Botswana/ or Burkina Faso/ or Burundi/ or Cameroon/ or Cape Verde/ or Central Africa/ or Central African Republic/ or Chad/ or Comoros/ or Congo/ or Cote d'Ivoire/ or Democratic Republic Congo/ or Djibouti/ or Equatorial Guinea/ or Eritrea/ or Eswatini/ or Ethiopia/ or Gabon/ or Gambia/ or Ghana/ or Guinea/ or Guinea-Bissau/ or Kenya/ or Lesotho/ or Liberia/ or Madagascar/ or Malawi/ or Mali/ or Mozambique/ or Namibia/ or Niger/ or Nigeria/ or Rwanda/ or Sahel/ or Senegal/ or Sierra Leone/ or Somalia/ or South Africa/ or South Sudan/ or Sudan/ or Tanzania/ or Togo/ or Uganda/ or Zambia/ or Zimbabwe/ or Algeria/ or Egypt/ or Libyan Arab Jamahiriya/ or Mauritania/ or Morocco/ or Tunisia/ or Western Sahara/ or Central Africa/ or North Africa/ or African Caribbean/ or Caribbean/ or Central America/ or "South and Central America"/ or Belize/ or Costa Rica/ or El Salvador/ or Guatemala/ or Honduras/ or Nicaragua/ or Panama/ or Antillean/ or Caribbean Islands/ or Cuba/ or Dominica/ or Dominican Republic/ or Grenada/ or Guadeloupe/ or Jamaica/ or Haiti/ or Martinique/ or Saint Lucia/ or "Saint Vincent and the Grenadines"/ or "caribbean (person)"/ or Cuban/ or "dominican (dominica)"/ or "dominican (dominican republic)"/ or Haitian/ or Jamaican/ or South America/ or Argentina/ or Bolivia/ or Brazil/ or Colombia/ or Ecuador/ or French Guiana/ or Guyana/ or Paraguay/ or Peru/ or Suriname/ or Venezuela/ or Mexico/ or Asia/ or central Asia/ or Far East/ or Middle East/ or northern Asia/ or South Asia/ or western Asia/ or Kazakhstan/ or Kyrgyzstan/ or Tajikistan/ or Turkmenistan/ or Uzbekistan/ or China/ or Korea/ or Mongolia/ or Philippines/ or Southeast Asia/ or North Korea/ or Borneo/ or Cambodia/ or Indonesia/ or Laos/ or Malaysia/ or Myanmar/ or Papua New Guinea/ or Singapore/ or Thailand/ or Timor-Leste/ or Viet Nam/ or Iran/ or Iraq/ or Jordan/ or Lebanon/ or Palestine/ or Syrian Arab Republic/ or "turkey (republic)"/ or Yemen/ or Afghanistan/ or Bangladesh/ or Bhutan/ or India/ or Nepal/ or Pakistan/ or Sri Lanka/ or Armenia/ or Azerbaijan/ or "georgia (republic)"/ or "Sao Tome and Principe"/ or Mauritius/ or Pacific Islands/ or Federated States of Micronesia/ or Fiji/ or Kiribati/ or Marshall Islands/ or Melanesia/ or Nauru/ or Palau/ or Polynesia/ or Samoan Islands/ or Solomon Islands/ or Timor-Leste/ or Tonga/ or Tuvalu/ or Vanuatu/ or American Samoa/ or Samoa/ or Romania/ or Russian Federation/ or USSR/ or Croatia/ or Albania/ or Belarus/ or "Bosnia and Herzegovina"/ or Bulgaria/ or Kosovo/ or Moldova/ or "Montenegro (republic)"/ or Republic of North Macedonia/ or Serbia/ or Ukraine/ or "Federation of Bosnia and Herzegovina"/ |
| 26 | (Afghanistan* or Albania* or Algeria* or Angola* or Argentina* or Armenia* or Azerbaijan* or Bangladesh* or Beliz* or Benin* or Bhutan* or Bolivia* or Bosnia* or Herzegovin* or Botswan* or Brazil* or Bulgaria* or Burkina* or Burundi* or Cabo Verde* or Cape Verde* or Cambodia* or Cameroon* or Chad* or China or Chinese or Colombia* or Comor* or Congo* or Costa Rica* or Cote d'Ivoir* or Ivory Coast or Cuba* or Djibouti* or Dominica* or Ecuador* or Egypt* or El Salvador* or Eritrea* or Ethiopia* or Fiji* or Gabon* or Gambia* or Georgia* or Ghana* or Grenad* or Guatemala* or Guinea* or Guyan* or Haiti* or Hondura* or Hungar* or India* or Indonesia* or Iran* or Iraq* or Jamaica* or Jordan* or Kazakhstan* or Kenya* or Kiribati* or Korea* or Kosov* or Kyrgyz* or Lao* or Leban* or Lesotho* or Liberia* or Libya* or Macedonia* or Madagascar* or Malawi* or Malaysia* or Maldiv* or Mali* or Marshall Island* or Mauritania* or Mauriti* or Mexic* or Moldova* or Mongolia* or Montenegr* or Morocc* or Mozambi* or Myanma* or Burmese or Namibia* or Nepal* or Nicaragua* or Niger* or Pakistan* or Palau* or Panama* or Papua New Guinea* or Paraguay* or Peru* or Philippines or Filipino or Romania* or Rwanda* or Samoa* or Sao Tome* or Senegal* or Serbia* or Seychell* or Sierra Leon* or Solomon Island* or Somalia* or Sudan* or Sri Lanka* or St Lucia* or Saint Lucia or St Vincent or Saint Vincent or Grenadines or Surinam* or Swazi* or Syria* or Tajikistan* or Tanzania* or Thai* or Timor* or Togo* or Tonga* or Tunisia* or Turk* or Tuvalu* or Uganda* or Ukrain* or Uzbekistan* or Vanuatu* or Venezuela* or Vietnam* or Viet-Nam* or West Bank or Gaza or Yemen* or Zambia* or Zimbabwe* or Russia* or Croatia* or Nauru* or Yugoslavia* or USSR or Soviet* or Byelarus* or Belarus* or (africa* or asia* or caribbean or central america* or latin america* or south america* or melanesia* or micronesia* or polynesia*)).mp. |
| 27 | (resource-limit* or resource-poor or low-resource* or limited-resource* or resource-constrain* or constrain*-resource* or under-resource* or poor*-resource* or resource-scarce* or scarce*-resource* or low-income or middle-income or lowincome or middleincome or (low* adj3 middle-income)).mp. |
| 28 | ((developing or underdeveloped or under-developed or emerging or less-developed or least-developed or less-economically developed or least-economically developed or less-affluent or least-affluent or least-industriali#ed or non-industriali#ed or deprived or poor) adj (country or countries or nation? or region? or economy or economies)).mp. |
| 29 | ((developing or underdeveloped or under-developed or less-developed or least-developed) adj (population* or world)).mp. |
| 30 | (third-world* or thirdworld* or 3rd-world* or lmic or lmics or lami countr* or lalmi countr* or transitional countr*).mp. |
| 31 | (low* adj (gdp or gnp or gross domestic or gross national)).mp. |
| 32 | ((underserved or under-served) adj (countr* or nation? or population*)).mp. |
| 33 | or/25-32 |
| 34 | 11 and 18 and 33 |
| 35 | 11 and 24 and 33 |
| 36 | exp nonsteroid antiinflammatory agent/ or exp prostaglandin synthase inhibitor/ or exp cyclooxygenase 2 inhibitor/ or tranexamic acid/ or exp oral contraceptive agent/ or intrauterine contraceptive device/ or levonorgestrel releasing intrauterine system/ or exp gestagen/ |
| 37 | (non-steroid* antiinflammatory or non-steroid* anti-inflammatory or nonsteroid antiinflammatory or nonsteroid* anti-inflammatory or NSAID or NSAIDs or (cyclooxygenase inhibitor* or cyclo-oxygenase inhibitor* or prostaglandin synthetase inhibitor* or cyclooxygenase-2 inhibitor* or COX-2 inhibitor* or COX-2 specific inhibitor* or COX2 inhibitor* or COX2 specific inhibitor* or coxib or coxibs)).mp. |
| 38 | (antifibrinolytic* or fibrinolysis inhibitor* or aminomethyl cyclohexane carboxylic acid* or aminomethyl cyclohexanecarboxylic acid* or aminomethylcyclohexane carbonic acid* or aminomethylcyclohexane carboxylic acid* or aminomethylcyclohexanecarbonic acid* or aminomethylcyclohexanecarboxylic acid* or aminomethylcyclohexanocarboxylic acid* or aminomethylcyclohexanoic acid* or tranexamic acid* or tranexam or tranexanic acid* or transexamic acid* or traxamic or TXA).mp. |
| 39 | (combined oral contraceptive* or combined hormonal contraceptive* or combined oral hormonal contraceptive* or combined vaginal ring* or combined hormonal vaginal ring* or contraceptive vaginal ring* or (luteal oral progestogen* or LNG-IUS or LNG-IUD or levonorgestrel IUD* or levonorgestrel releasing intrauterine or levonorgestrel releasing IUD or levonorgestrel releasing intra-uterine or levonorgestrel intrauterine system or levonorgestrel intrauterine device) or (gestogen* or progestagen* or progestin* or progestogen*)).mp. |
| 40 | or/36-39 |
| 41 | 11 and 40 |
| 42 | 34 or 35 or 41 |
| 43 | limit 42 to yr="2000 -Current" |
| 44 | (exp animal/ or nonhuman/ or exp invertebrate/ or animal.hw.) not exp human/ |
| 45 | 43 not 44 |
| 46 | limit 45 to (editorial or letter or conference abstract) |
| **47** | **45 not 46** |

**New records since June 2023**

| 48 | (202306* or 202307* or 202308* or 202309* or 202310* or 202311* or 202312* or 202401* or 202402* or 202403* or 202404* or 202405* or 202406* or 202407* or 202408* or 202409* or 202410*).dc,dp,yr. |
| --- | --- |
| 49 | 47 and 48 |
| 50 | limit 47 to yr="2024 -Current" |
| **51** | **49 or 50** |

**Database:** Global Health

| **#** | **Query** |
| --- | --- |
| 1 | (meno?rhagi* or menorr?agi* or menor*agi* or hypermeno?rhea* or hypermenorr?oea* or hypermeno?rh?ea* or meno?rhagy or menorr?agy or polymenor?hea* or polymenorr?ea or polymeno?rh?ea or menometro?rhagi* or menometrorr?agi* or menometror*agi? or Menstrua* morbidity).mp. |
| 2 | (((severe or severity or massive* or excessive* or extensive* or large scale or acute or unrelenting or overwhelming or uncontrolled or extreme or significant or heavy or heavier or debilitat* or prolonged or excess or dysfunctional or abnormal or abundant or profuse) adj3 (bleed* or blood loss* or blood flow* or h?em?or*ag*)) and (menstrua* or menses or catamenia or menstruum)).mp. |
| 3 | ((heavy or massive* or excessive* or extensive* or debilitat* or prolonged or dysfunctional or abnormal or severe) adj (menstrua* or menses or catamenia or menstruum)).mp. |
| 4 | ((heavy adj (period? or MBL)) or (HMB adj2 (AUB or MBL or DUB))).mp. |
| 5 | ((long* duration or excessive duration or prolonged duration or excessively long or prolonged period* or heavy flow or excessive flow or prolonged flow or abnormal flow or severe flow) adj5 (menstrua* or menses or catamenia or menstruum)).mp. |
| 6 | ((suffer* or disabl* or debilitat* or burden* or impact*) adj3 HMB).mp. |
| 7 | ((bleeding or blood loss) adj3 (score? or pattern? or measur* or assess* or amount? or evaluat*) adj3 (menstrua* or menses or catamenia or menstruum or MBL)).mp. |
| 8 | ((abnormal or excessive) adj (uterine bleeding or uterine blood loss)).mp. |
| 9 | ((ovulatory disorder* or adenomyosis or endometrios* or endometrial polyp? or endometrial hyperplasia or uterine fibroid? or leiomyomata) and ((severe or severity or massive* or excessive* or extensive* or large scale or acute or unrelenting or overwhelming or uncontrolled or extreme or significant or heavy or heavier or debilitat* or prolonged or excess or dysfunctional or abnormal or abundant or profuse) adj3 (bleed* or blood loss* or blood flow* or h?em?or*ag*))).mp. |
| 10 | or/1-9 |
| 11 | "quality of life"/ or mental health/ or (QOL or HRQOL or DALY or QALY or quality of life or life quality or experience* or burden*).mp. |
| 12 | consumer satisfaction/ or self perception/ or self esteem/ or attitudes/ or attitudes to health/ or consumer attitudes/ or health behaviour/ |
| 13 | ("living day to day" or "lives of" or "living with" or normal life or normal lives or ((every day or everyday or daily) adj (life or living or routine* or functioning or activit* or interest?)) or ((realities or difficulties) adj2 (life or living)) or (life quality or life satisfaction or "satisf* with life") or (school life or work life or social life or family life or future life or future lives or life activit* or psychosocial health or mental health or wellbeing or well-being or emotional function* or school functioning)).mp. |
| 14 | (self concept or self belief or self image or self advoca* or self aware* or self help or self esteem or "sense of self" or self worth or self achiev* or self confidence or self regard).mp. |
| 15 | ((impact* or affect* or difficulties or problems or dysfunction* or disrupt* or disturb* or implication* or implications or concerns or worries or worrying or adjustment* or interfer* or imping* or limited or limiting) adj3 (physical or social or emotional or psychosocial or disabilit* or disabl* or abilit* or life* or living or lives or functioning or activit*)).mp. |
| 16 | ((impact* or interfere* or affect* or difficulties or problems or dysfunction* or disordered or disrupt* or disturb* or implications or concerns or worries or worrying or adjustment or interfer* or imping*) adj3 (studying or school or training or learning or employment or work or job or jobs or performance or studies or occupation* or responsibilit* or roles or future*)).mp. |
| 17 | or/11-16 |
| 18 | (cultural* or transcultural or ethnic* or racial* or stigma*).mp. |
| 19 | (access* or need* or seek* or accept* or prefer* or trust* or encounter* or understand or satisfaction or dissatisfaction or acceptable or perceive* or perception* or expectation* or wait* time* or wait* list* or timeliness or delay* or price* or pricing or fund* or availability or available or cost* or monetary or budget* or financ* or reimburs* or payer* or payee or investment* or access or accessibility or accessible* or supply or market* or affordability or affordable* or quality or pre-qualified or off-label or inequit* or inequalit* or disbursement? or expenditure or expense? or expensive* or remittance or recompens* or repayment? or refund* or recoup* or rebate? or compensate? or compensation or prepay* or overpay* or underpay*).mp. |
| 20 | (Barrier* or facilitat* or enable* or enabling or challenge* or attitude* or belief* or views* or behavio?r* or influenc* or adopt* or uptake or eligibility or eligible or value* or economic* or discount* or payment* or provision* or choice? or "willingness to pay" or repurposed or universal health coverage or stringent regulatory authorit* or national regulatory authorit* or procurement or registration or infrastructure or remuneration or safety or efficacy).mp. |
| 21 | ((restrict* or limit*) and (distribut* or prescri* or dispens*)).mp. |
| 22 | ((counterfeit or fake or false or falsified) adj (drug* or medicine* or medication*)).mp. |
| 23 | or/18-22 |
| 24 | low income countries/ or lower-middle income countries/ or low human development index countries/ or medium human development index countries/ or least developed countries/ or Africa/ or Africa south of the Sahara/ or North Africa/ or angola/ or benin/ or Botswana/ or Burkina Faso/ or Burundi/ or Cameroon/ or Cape Verde/ or Central Africa/ or Central African Republic/ or Chad/ or Comoros/ or Congo/ or Cote d'Ivoire/ or Democratic Republic Congo/ or Djibouti/ or Equatorial Guinea/ or Eritrea/ or Eswatini/ or Ethiopia/ or Gabon/ or Gambia/ or Ghana/ or Guinea/ or Guinea-Bissau/ or Kenya/ or Lesotho/ or Liberia/ or Madagascar/ or Malawi/ or Mali/ or Mozambique/ or Namibia/ or Niger/ or Nigeria/ or Rwanda/ or Sahel/ or Senegal/ or Sierra Leone/ or Somalia/ or South Africa/ or South Sudan/ or Sudan/ or Tanzania/ or Togo/ or Uganda/ or Zambia/ or Zimbabwe/ or Algeria/ or Egypt/ or Libyan Arab Jamahiriya/ or Mauritania/ or Morocco/ or Tunisia/ or Western Sahara/ or Central Africa/ or North Africa/ or African Caribbean/ or Caribbean/ or Central America/ or "South and Central America"/ or Belize/ or Costa Rica/ or El Salvador/ or Guatemala/ or Honduras/ or Nicaragua/ or Panama/ or Antillean/ or Caribbean Islands/ or Cuba/ or Dominica/ or Dominican Republic/ or Grenada/ or Guadeloupe/ or Jamaica/ or Haiti/ or Martinique/ or Saint Lucia/ or "Saint Vincent and the Grenadines"/ or "caribbean (person)"/ or Cuban/ or "dominican (dominica)"/ or "dominican (dominican republic)"/ or Haitian/ or Jamaican/ or South America/ or Argentina/ or Bolivia/ or Brazil/ or Colombia/ or Ecuador/ or French Guiana/ or Guyana/ or Paraguay/ or Peru/ or Suriname/ or Venezuela/ or Mexico/ or Asia/ or central Asia/ or Far East/ or Middle East/ or northern Asia/ or South Asia/ or western Asia/ or Kazakhstan/ or Kyrgyzstan/ or Tajikistan/ or Turkmenistan/ or Uzbekistan/ or China/ or Korea/ or Mongolia/ or Philippines/ or Southeast Asia/ or North Korea/ or Borneo/ or Cambodia/ or Indonesia/ or Laos/ or Malaysia/ or Myanmar/ or Papua New Guinea/ or Singapore/ or Thailand/ or Timor-Leste/ or Viet Nam/ or Iran/ or Iraq/ or Jordan/ or Lebanon/ or Palestine/ or Syrian Arab Republic/ or "turkey (republic)"/ or Yemen/ or Afghanistan/ or Bangladesh/ or Bhutan/ or India/ or Nepal/ or Pakistan/ or Sri Lanka/ or Armenia/ or Azerbaijan/ or "georgia (republic)"/ or "Sao Tome and Principe"/ or Mauritius/ or Pacific Islands/ or Federated States of Micronesia/ or Fiji/ or Kiribati/ or Marshall Islands/ or Melanesia/ or Nauru/ or Palau/ or Polynesia/ or Samoan Islands/ or Solomon Islands/ or Timor-Leste/ or Tonga/ or Tuvalu/ or Vanuatu/ or American Samoa/ or Samoa/ or Romania/ or Russian Federation/ or USSR/ or Croatia/ or Albania/ or Belarus/ or "Bosnia and Herzegovina"/ or Bulgaria/ or Kosovo/ or Moldova/ or "Montenegro (republic)"/ or Republic of North Macedonia/ or Serbia/ or Ukraine/ or "Federation of Bosnia and Herzegovina"/ |
| 25 | (Afghanistan* or Albania* or Algeria* or Angola* or Argentina* or Armenia* or Azerbaijan* or Bangladesh* or Beliz* or Benin* or Bhutan* or Bolivia* or Bosnia* or Herzegovin* or Botswan* or Brazil* or Bulgaria* or Burkina* or Burundi* or Cabo Verde* or Cape Verde* or Cambodia* or Cameroon* or Chad* or China or Chinese or Colombia* or Comor* or Congo* or Costa Rica* or Cote d'Ivoir* or Ivory Coast or Cuba* or Djibouti* or Dominica* or Ecuador* or Egypt* or El Salvador* or Eritrea* or Ethiopia* or Fiji* or Gabon* or Gambia* or Georgia* or Ghana* or Grenad* or Guatemala* or Guinea* or Guyan* or Haiti* or Hondura* or Hungar* or India* or Indonesia* or Iran* or Iraq* or Jamaica* or Jordan* or Kazakhstan* or Kenya* or Kiribati* or Korea* or Kosov* or Kyrgyz* or Lao* or Leban* or Lesotho* or Liberia* or Libya* or Macedonia* or Madagascar* or Malawi* or Malaysia* or Maldiv* or Mali* or Marshall Island* or Mauritania* or Mauriti* or Mexic* or Moldova* or Mongolia* or Montenegr* or Morocc* or Mozambi* or Myanma* or Burmese or Namibia* or Nepal* or Nicaragua* or Niger* or Pakistan* or Palau* or Panama* or Papua New Guinea* or Paraguay* or Peru* or Philippines or Filipino or Romania* or Rwanda* or Samoa* or Sao Tome* or Senegal* or Serbia* or Seychell* or Sierra Leon* or Solomon Island* or Somalia* or Sudan* or Sri Lanka* or St Lucia* or Saint Lucia or St Vincent or Saint Vincent or Grenadines or Surinam* or Swazi* or Syria* or Tajikistan* or Tanzania* or Thai* or Timor* or Togo* or Tonga* or Tunisia* or Turk* or Tuvalu* or Uganda* or Ukrain* or Uzbekistan* or Vanuatu* or Venezuela* or Vietnam* or Viet-Nam* or West Bank or Gaza or Yemen* or Zambia* or Zimbabwe* or Russia* or Croatia* or Nauru* or Yugoslavia* or USSR or Soviet* or Byelarus* or Belarus* or (africa* or asia* or caribbean or central america* or latin america* or south america* or melanesia* or micronesia* or polynesia*)).mp. |
| 26 | (resource-limit* or resource-poor or low-resource* or limited-resource* or resource-constrain* or constrain*-resource* or under-resource* or poor*-resource* or resource-scarce* or scarce*-resource* or low-income or middle-income or lowincome or middleincome or (low* adj3 middle-income)).mp. |
| 27 | ((developing or underdeveloped or under-developed or emerging or less-developed or least-developed or less-economically developed or least-economically developed or less-affluent or least-affluent or least-industriali#ed or non-industriali#ed or deprived or poor) adj (country or countries or nation? or region? or economy or economies)).mp. |
| 28 | ((developing or underdeveloped or under-developed or less-developed or least-developed) adj (population* or world)).mp. |
| 29 | (third-world* or thirdworld* or 3rd-world* or lmic or lmics or lami countr* or lalmi countr* or transitional countr*).mp. |
| 30 | (low* adj (gdp or gnp or gross domestic or gross national)).mp. |
| 31 | ((underserved or under-served) adj (countr* or nation? or population*)).mp. |
| 32 | or/24-31 |
| 33 | 10 and 17 and 32 |
| 34 | 10 and 23 and 32 |
| 35 | exp non-steroidal antiinflammatory agents/ or exp oral contraceptives/ or intrauterine devices/ |
| 36 | (non-steroid* antiinflammatory or non-steroid* anti-inflammatory or nonsteroid antiinflammatory or nonsteroid* anti-inflammatory or NSAID or NSAIDs or (cyclooxygenase inhibitor* or cyclo-oxygenase inhibitor* or prostaglandin synthetase inhibitor* or cyclooxygenase-2 inhibitor* or COX-2 inhibitor* or COX-2 specific inhibitor* or COX2 inhibitor* or COX2 specific inhibitor* or coxib or coxibs)).mp. |
| 37 | (antifibrinolytic* or fibrinolysis inhibitor* or aminomethyl cyclohexane carboxylic acid* or aminomethyl cyclohexanecarboxylic acid* or aminomethylcyclohexane carbonic acid* or aminomethylcyclohexane carboxylic acid* or aminomethylcyclohexanecarbonic acid* or aminomethylcyclohexanecarboxylic acid* or aminomethylcyclohexanocarboxylic acid* or aminomethylcyclohexanoic acid* or tranexamic acid* or tranexam or tranexanic acid* or transexamic acid* or traxamic or TXA).mp. |
| 38 | (combined oral contraceptive* or combined hormonal contraceptive* or combined oral hormonal contraceptive* or combined vaginal ring* or combined hormonal vaginal ring* or contraceptive vaginal ring* or (luteal oral progestogen* or LNG-IUS or LNG-IUD or levonorgestrel IUD* or levonorgestrel releasing intrauterine or levonorgestrel releasing IUD or levonorgestrel releasing intra-uterine or levonorgestrel intrauterine system or levonorgestrel intrauterine device) or (gestogen* or progestagen* or progestin* or progestogen*)).mp. |
| 39 | or/35-38 |
| 40 | 10 and 39 |
| 41 | 33 or 34 or 40 |
| **42** | **limit 41 to yr="2000 -Current"** |

**New results for Global Health (unable to drill down by month so 2023 to 2024)**

| 43 | limit 42 to yr="2024 -Current" |
| --- | --- |
| 44 | limit 42 to yr="2023" |
| **45** | **43 or 44** |

**Database:** APA PsycInfo

| **#** | **Query** |
| --- | --- |
| 1 | (meno?rhagi* or menorr?agi* or menor*agi* or hypermeno?rhea* or hypermenorr?oea* or hypermeno?rh?ea* or meno?rhagy or menorr?agy or polymenor?hea* or polymenorr?ea or polymeno?rh?ea or menometro?rhagi* or menometrorr?agi* or menometror*agi? or Menstrua* morbidity).mp. |
| 2 | (((severe or severity or massive* or excessive* or extensive* or large scale or acute or unrelenting or overwhelming or uncontrolled or extreme or significant or heavy or heavier or debilitat* or prolonged or excess or dysfunctional or abnormal or abundant or profuse) adj3 (bleed* or blood loss* or blood flow* or h?em?or*ag*)) and (menstrua* or menses or catamenia or menstruum)).mp. |
| 3 | ((heavy or massive* or excessive* or extensive* or debilitat* or prolonged or dysfunctional or abnormal or severe) adj (menstrua* or menses or catamenia or menstruum)).mp. |
| 4 | ((heavy adj (period? or MBL)) or (HMB adj2 (AUB or MBL or DUB))).mp. |
| 5 | ((long* duration or excessive duration or prolonged duration or excessively long or prolonged period* or heavy flow or excessive flow or prolonged flow or abnormal flow or severe flow) adj5 (menstrua* or menses or catamenia or menstruum)).mp. |
| 6 | ((suffer* or disabl* or debilitat* or burden* or impact*) adj3 HMB).mp. |
| 7 | ((bleeding or blood loss) adj3 (score? or pattern? or measur* or assess* or amount? or evaluat*) adj3 (menstrua* or menses or catamenia or menstruum or MBL)).mp. |
| 8 | ((abnormal or excessive) adj (uterine bleeding or uterine blood loss)).mp. |
| 9 | ((ovulatory disorder* or adenomyosis or endometrios* or endometrial polyp? or endometrial hyperplasia or uterine fibroid? or leiomyomata) and ((severe or severity or massive* or excessive* or extensive* or large scale or acute or unrelenting or overwhelming or uncontrolled or extreme or significant or heavy or heavier or debilitat* or prolonged or excess or dysfunctional or abnormal or abundant or profuse) adj3 (bleed* or blood loss* or blood flow* or h?em?or*ag*))).mp. |
| 10 | or/1-9 |
| 11 | "quality of life"/ or mental health/ or (QOL or HRQOL or DALY or QALY or quality of life or life quality or experience* or burden*).mp. |
| 12 | consumer satisfaction/ or self perception/ or self esteem/ or attitudes/ or attitudes to health/ or consumer attitudes/ or health behaviour/ |
| 13 | ("living day to day" or "lives of" or "living with" or normal life or normal lives or ((every day or everyday or daily) adj (life or living or routine* or functioning or activit* or interest?)) or ((realities or difficulties) adj2 (life or living)) or (life quality or life satisfaction or "satisf* with life") or (school life or work life or social life or family life or future life or future lives or life activit* or psychosocial health or mental health or wellbeing or well-being or emotional function* or school functioning)).mp. |
| 14 | (self concept or self belief or self image or self advoca* or self aware* or self help or self esteem or "sense of self" or self worth or self achiev* or self confidence or self regard).mp. |
| 15 | ((impact* or affect* or difficulties or problems or dysfunction* or disrupt* or disturb* or implication* or implications or concerns or worries or worrying or adjustment* or interfer* or imping* or limited or limiting) adj3 (physical or social or emotional or psychosocial or disabilit* or disabl* or abilit* or life* or living or lives or functioning or activit*)).mp. |
| 16 | ((impact* or interfere* or affect* or difficulties or problems or dysfunction* or disordered or disrupt* or disturb* or implications or concerns or worries or worrying or adjustment or interfer* or imping*) adj3 (studying or school or training or learning or employment or work or job or jobs or performance or studies or occupation* or responsibilit* or roles or future*)).mp. |
| 17 | or/11-16 |
| 18 | (cultural* or transcultural or ethnic* or racial* or stigma*).mp. |
| 19 | (access* or need* or seek* or accept* or prefer* or trust* or encounter* or understand or satisfaction or dissatisfaction or acceptable or perceive* or perception* or expectation* or wait* time* or wait* list* or timeliness or delay* or price* or pricing or fund* or availability or available or cost* or monetary or budget* or financ* or reimburs* or payer* or payee or investment* or access or accessibility or accessible* or supply or market* or affordability or affordable* or quality or pre-qualified or off-label or inequit* or inequalit* or disbursement? or expenditure or expense? or expensive* or remittance or recompens* or repayment? or refund* or recoup* or rebate? or compensate? or compensation or prepay* or overpay* or underpay*).mp. |
| 20 | (Barrier* or facilitat* or enable* or enabling or challenge* or attitude* or belief* or views* or behavio?r* or influenc* or adopt* or uptake or eligibility or eligible or value* or economic* or discount* or payment* or provision* or choice? or "willingness to pay" or repurposed or universal health coverage or stringent regulatory authorit* or national regulatory authorit* or procurement or registration or infrastructure or remuneration or safety or efficacy).mp. |
| 21 | ((restrict* or limit*) and (distribut* or prescri* or dispens*)).mp. |
| 22 | ((counterfeit or fake or false or falsified) adj (drug* or medicine* or medication*)).mp. |
| 23 | or/18-22 |
| 24 | low income countries/ or lower-middle income countries/ or low human development index countries/ or medium human development index countries/ or least developed countries/ or Africa/ or Africa south of the Sahara/ or North Africa/ or angola/ or benin/ or Botswana/ or Burkina Faso/ or Burundi/ or Cameroon/ or Cape Verde/ or Central Africa/ or Central African Republic/ or Chad/ or Comoros/ or Congo/ or Cote d'Ivoire/ or Democratic Republic Congo/ or Djibouti/ or Equatorial Guinea/ or Eritrea/ or Eswatini/ or Ethiopia/ or Gabon/ or Gambia/ or Ghana/ or Guinea/ or Guinea-Bissau/ or Kenya/ or Lesotho/ or Liberia/ or Madagascar/ or Malawi/ or Mali/ or Mozambique/ or Namibia/ or Niger/ or Nigeria/ or Rwanda/ or Sahel/ or Senegal/ or Sierra Leone/ or Somalia/ or South Africa/ or South Sudan/ or Sudan/ or Tanzania/ or Togo/ or Uganda/ or Zambia/ or Zimbabwe/ or Algeria/ or Egypt/ or Libyan Arab Jamahiriya/ or Mauritania/ or Morocco/ or Tunisia/ or Western Sahara/ or Central Africa/ or North Africa/ or African Caribbean/ or Caribbean/ or Central America/ or "South and Central America"/ or Belize/ or Costa Rica/ or El Salvador/ or Guatemala/ or Honduras/ or Nicaragua/ or Panama/ or Antillean/ or Caribbean Islands/ or Cuba/ or Dominica/ or Dominican Republic/ or Grenada/ or Guadeloupe/ or Jamaica/ or Haiti/ or Martinique/ or Saint Lucia/ or "Saint Vincent and the Grenadines"/ or "caribbean (person)"/ or Cuban/ or "dominican (dominica)"/ or "dominican (dominican republic)"/ or Haitian/ or Jamaican/ or South America/ or Argentina/ or Bolivia/ or Brazil/ or Colombia/ or Ecuador/ or French Guiana/ or Guyana/ or Paraguay/ or Peru/ or Suriname/ or Venezuela/ or Mexico/ or Asia/ or central Asia/ or Far East/ or Middle East/ or northern Asia/ or South Asia/ or western Asia/ or Kazakhstan/ or Kyrgyzstan/ or Tajikistan/ or Turkmenistan/ or Uzbekistan/ or China/ or Korea/ or Mongolia/ or Philippines/ or Southeast Asia/ or North Korea/ or Borneo/ or Cambodia/ or Indonesia/ or Laos/ or Malaysia/ or Myanmar/ or Papua New Guinea/ or Singapore/ or Thailand/ or Timor-Leste/ or Viet Nam/ or Iran/ or Iraq/ or Jordan/ or Lebanon/ or Palestine/ or Syrian Arab Republic/ or "turkey (republic)"/ or Yemen/ or Afghanistan/ or Bangladesh/ or Bhutan/ or India/ or Nepal/ or Pakistan/ or Sri Lanka/ or Armenia/ or Azerbaijan/ or "georgia (republic)"/ or "Sao Tome and Principe"/ or Mauritius/ or Pacific Islands/ or Federated States of Micronesia/ or Fiji/ or Kiribati/ or Marshall Islands/ or Melanesia/ or Nauru/ or Palau/ or Polynesia/ or Samoan Islands/ or Solomon Islands/ or Timor-Leste/ or Tonga/ or Tuvalu/ or Vanuatu/ or American Samoa/ or Samoa/ or Romania/ or Russian Federation/ or USSR/ or Croatia/ or Albania/ or Belarus/ or "Bosnia and Herzegovina"/ or Bulgaria/ or Kosovo/ or Moldova/ or "Montenegro (republic)"/ or Republic of North Macedonia/ or Serbia/ or Ukraine/ or "Federation of Bosnia and Herzegovina"/ |
| 25 | (Afghanistan* or Albania* or Algeria* or Angola* or Argentina* or Armenia* or Azerbaijan* or Bangladesh* or Beliz* or Benin* or Bhutan* or Bolivia* or Bosnia* or Herzegovin* or Botswan* or Brazil* or Bulgaria* or Burkina* or Burundi* or Cabo Verde* or Cape Verde* or Cambodia* or Cameroon* or Chad* or China or Chinese or Colombia* or Comor* or Congo* or Costa Rica* or Cote d'Ivoir* or Ivory Coast or Cuba* or Djibouti* or Dominica* or Ecuador* or Egypt* or El Salvador* or Eritrea* or Ethiopia* or Fiji* or Gabon* or Gambia* or Georgia* or Ghana* or Grenad* or Guatemala* or Guinea* or Guyan* or Haiti* or Hondura* or Hungar* or India* or Indonesia* or Iran* or Iraq* or Jamaica* or Jordan* or Kazakhstan* or Kenya* or Kiribati* or Korea* or Kosov* or Kyrgyz* or Lao* or Leban* or Lesotho* or Liberia* or Libya* or Macedonia* or Madagascar* or Malawi* or Malaysia* or Maldiv* or Mali* or Marshall Island* or Mauritania* or Mauriti* or Mexic* or Moldova* or Mongolia* or Montenegr* or Morocc* or Mozambi* or Myanma* or Burmese or Namibia* or Nepal* or Nicaragua* or Niger* or Pakistan* or Palau* or Panama* or Papua New Guinea* or Paraguay* or Peru* or Philippines or Filipino or Romania* or Rwanda* or Samoa* or Sao Tome* or Senegal* or Serbia* or Seychell* or Sierra Leon* or Solomon Island* or Somalia* or Sudan* or Sri Lanka* or St Lucia* or Saint Lucia or St Vincent or Saint Vincent or Grenadines or Surinam* or Swazi* or Syria* or Tajikistan* or Tanzania* or Thai* or Timor* or Togo* or Tonga* or Tunisia* or Turk* or Tuvalu* or Uganda* or Ukrain* or Uzbekistan* or Vanuatu* or Venezuela* or Vietnam* or Viet-Nam* or West Bank or Gaza or Yemen* or Zambia* or Zimbabwe* or Russia* or Croatia* or Nauru* or Yugoslavia* or USSR or Soviet* or Byelarus* or Belarus* or (africa* or asia* or caribbean or central america* or latin america* or south america* or melanesia* or micronesia* or polynesia*)).mp. |
| 26 | (resource-limit* or resource-poor or low-resource* or limited-resource* or resource-constrain* or constrain*-resource* or under-resource* or poor*-resource* or resource-scarce* or scarce*-resource* or low-income or middle-income or lowincome or middleincome or (low* adj3 middle-income)).mp. |
| 27 | ((developing or underdeveloped or under-developed or emerging or less-developed or least-developed or less-economically developed or least-economically developed or less-affluent or least-affluent or least-industriali#ed or non-industriali#ed or deprived or poor) adj (country or countries or nation? or region? or economy or economies)).mp. |
| 28 | ((developing or underdeveloped or under-developed or less-developed or least-developed) adj (population* or world)).mp. |
| 29 | (third-world* or thirdworld* or 3rd-world* or lmic or lmics or lami countr* or lalmi countr* or transitional countr*).mp. |
| 30 | (low* adj (gdp or gnp or gross domestic or gross national)).mp. |
| 31 | ((underserved or under-served) adj (countr* or nation? or population*)).mp. |
| 32 | or/24-31 |
| 33 | 10 and 17 and 32 |
| 34 | 10 and 23 and 32 |
| 35 | exp non-steroidal antiinflammatory agents/ or exp oral contraceptives/ or intrauterine devices/ |
| 36 | (non-steroid* antiinflammatory or non-steroid* anti-inflammatory or nonsteroid antiinflammatory or nonsteroid* anti-inflammatory or NSAID or NSAIDs or (cyclooxygenase inhibitor* or cyclo-oxygenase inhibitor* or prostaglandin synthetase inhibitor* or cyclooxygenase-2 inhibitor* or COX-2 inhibitor* or COX-2 specific inhibitor* or COX2 inhibitor* or COX2 specific inhibitor* or coxib or coxibs)).mp. |
| 37 | (antifibrinolytic* or fibrinolysis inhibitor* or aminomethyl cyclohexane carboxylic acid* or aminomethyl cyclohexanecarboxylic acid* or aminomethylcyclohexane carbonic acid* or aminomethylcyclohexane carboxylic acid* or aminomethylcyclohexanecarbonic acid* or aminomethylcyclohexanecarboxylic acid* or aminomethylcyclohexanocarboxylic acid* or aminomethylcyclohexanoic acid* or tranexamic acid* or tranexam or tranexanic acid* or transexamic acid* or traxamic or TXA).mp. |
| 38 | (combined oral contraceptive* or combined hormonal contraceptive* or combined oral hormonal contraceptive* or combined vaginal ring* or combined hormonal vaginal ring* or contraceptive vaginal ring* or (luteal oral progestogen* or LNG-IUS or LNG-IUD or levonorgestrel IUD* or levonorgestrel releasing intrauterine or levonorgestrel releasing IUD or levonorgestrel releasing intra-uterine or levonorgestrel intrauterine system or levonorgestrel intrauterine device) or (gestogen* or progestagen* or progestin* or progestogen*)).mp. |
| 39 | or/35-38 |
| 40 | 10 and 39 |
| 41 | 33 or 34 or 40 |
| **42** | **limit 41 to yr="2000 -Current"** |

**New records since June 2023**

| 43 | (202306* or 202307* or 202308* or 202309* or 202310* or 202311* or 202312* or 202401* or 202402* or 202403* or 202404* or 202405* or 202406* or 202407* or 202408* or 202409* or 202410*).up. |
| --- | --- |
| 44 | 42 and 43 |
| 45 | limit 42 to yr="2024 -Current" |
| **46** | **44 or 45** |

**DATABASE: CINAHL**

| **#** | **Query** |
| --- | --- |
| S1 | (MH "Menorrhagia") OR ( (meno?rhagi* or menorr?agi* or menor*agi* or hypermeno?rhea* or hypermenorr?oea* or hypermeno?rh?ea* or meno?rhagy or menorr?agy or polymenor?hea* or polymenorr?ea or polymeno?rh?ea or menometro?rhagi* or menometrorr?agi* or menometror*agi? or "Menstrua* morbidity") ) |
| S2 | ( (((severe or severity or massive* or excessive* or extensive* or “large scale” or acute or unrelenting or overwhelming or uncontrolled or extreme or significant or heavy or heavier or debilitat* or prolonged or excess or dysfunctional or abnormal or abundant or profuse) N2 (bleed* or “blood loss*” or “blood flow*” or h?em?or*ag*)) and (menstrua* or menses or catamenia or menstruum)) ) OR ( ((heavy or massive* or excessive* or extensive* or debilitat* or prolonged or dysfunctional or abnormal or severe) N0 (menstrua* or menses or catamenia or menstruum)) ) |
| S3 | ( (heavy N0 (period? or MBL)) ) OR ( (HMB N2 (AUB or MBL or DUB)) ) |
| S4 | ( (("long* duration" or "excessive duration" or "prolonged duration" or "excessively long" or "prolonged period*" or "heavy flow") N4 (menstrua* or menses or catamenia or menstruum)) ) OR ( ((suffer* or disabl* or debilitat* or burden* or impact*) N2 HMB) ) |
| S5 | ( ((bleeding or “blood loss”) N2 (score? or pattern? or measur* or assess* or amount? or evaluat*) N2 (menstrua* or menses or catamenia or menstruum or MBL)) ) OR ( ((abnormal or excessive) N0 ("uterine bleeding" or "uterine blood loss")) ) |
| S6 | ((“ovulatory disorder*” or adenomyosis or endometrios* or “endometrial polyp?” or “endometrial hyperplasia” or “uterine fibroid?” or leiomyomata) and ((severe or severity or massive* or excessive* or extensive* or “large scale” or acute or unrelenting or overwhelming or uncontrolled or extreme or significant or heavy or heavier or debilitat* or prolonged or excess or dysfunctional or abnormal or abundant or profuse) N2 (bleed* or “blood loss*” or “blood flow*” or h?em?or*ag*))) |
| S7 | S1 OR S2 OR S3 OR S4 OR S5 OR S6 |
| S8 | ( ( (MH "Quality of Life") OR (MH "Psychological Well-Being") ) OR ( (MH "Quality-Adjusted Life Years") OR (MH "Disability-Adjusted Life Years") ) ) OR ( (QOL or HRQOL or DALY or QALY or "quality of life" or "life quality" or experience* or burden*) ) |
| S9 | ( (MH "Autonomy") OR (MH "Personal Satisfaction") OR (MH "Patient Satisfaction") OR (MH "Consumer Satisfaction") OR (MH "Attitude") OR (MH "Attitude to Health") OR (MH "Self Concept") OR (MH "Self-Compassion") OR (MH "Self Disclosure") OR (MH "Self-Efficacy") OR (MH "Self Assessment") OR (MH "Health Behavior") OR (MH "Health Knowledge") OR (MH "Patient Preference") OR (MH "Patient Attitudes") ) OR ( ("living day to day" or "lives of" or "living with" or “normal life” or “normal lives” or ((“every day” or everyday or daily) N0 (life or living or routine* or functioning or activit* or interest?)) or ((realities or difficulties) N1 (life or living)) or (“life quality” or “life satisfaction” or "satisf* with life") or (“school life” or “work life” or “social life” or “family life” or “future life” or “future lives” or "life activit*" or "psychosocial health" or "mental health" or wellbeing or "well-being" or "emotional function*" or "school functioning" or "self concept" or "self belief" or "self image" or "self advoca*" or "self aware*" or "self help" or "self esteem" or "sense of self" or "self worth" or "self achiev*" or "self confidence" or "self regard")) ) |
| S10 | ( ((impact* or affect* or difficulties or problems or dysfunction* or disrupt* or disturb* or implication* or implications or concerns or worries or worrying or adjustment* or interfer* or imping* or limited or limiting) N2 (physical or disabilit* or disabl* or abilit* or life or living or lives or functioning or activit*)) ) OR ( ((impact* or interfere* or affect* or difficulties or problems or dysfunction* or disordered or disrupt* or disturb* or implications or concerns or worries or worrying or adjustment or interfer* or imping*) N2 (studying or school or training or learning or employment or work or job or jobs or performance or studies or occupation* or responsibilit* or roles or future*)) ) |
| S11 | S8 OR S9 OR S10 |
| S12 | (cultural* or transcultural or ethnic* or racial* or stigma*) |
| S13 | ( (access* or need* or seek* or accept* or prefer* or trust* or encounter* or understand or satisfaction or dissatisfaction or acceptable or perceive* or perception* or expectation* or "wait* time*" or "wait* list*" or timeliness or delay*) ) OR ( (price* or pricing or fund* or availability or available or cost* or monetary or budget* or financ* or reimburs* or payer* or payee or investment* or access or accessibility or accessible* or supply or market* or affordability or affordable* or quality or "pre-qualified" or "off-label" or inequit* or inequalit*) ) OR ( (disbursement? or expenditure or expense? or expensive* or remittance or recompens* or repayment? or refund* or recoup* or rebate? or compensate? or compensation or prepay* or overpay* or underpay*) ) OR ( (Barrier* or facilitat* or enable* or enabling or challenge* or attitude* or belief* or views* or behavio?r* or influenc* or adopt* or uptake or eligibility or eligible or value* or economic* or discount* or payment* or provision* or choice? or "willingness to pay") ) OR ( (repurposed or "universal health coverage" or "stringent regulatory authorit*" or "national regulatory authorit*" or procurement or registration or infrastructure or remuneration) ) OR ( (safety or efficacy) ) |
| S14 | ( ((restrict* or limit*) and (distribut* or prescri* or dispens*)) ) OR ( ((counterfeit or fake or false or falsified) N0 (drug* or medicine* or medication*)) ) |
| S15 | S12 OR S13 OR S14 |
| S16 | ( (MH "Developing Countries") OR ( MH “Low and Middle Income Countries” OR MH “Developing countries” OR MH “Africa” OR MH “Africa South of the Sahara” OR MH “Africa, Western” OR MH “Africa, Southern” OR MH “Africa, Northern” OR MH “Africa, Eastern” OR MH “Africa, Central” OR MH “South Africa” OR MH “Namibia” OR MH “Algeria” OR MH “Egypt” OR MH “Libya” OR MH “Morocco” OR MH “Tunisia” OR MH “Cameroon” OR MH “Central African Republic” OR MH “Chad” OR MH “Congo” OR MH “Democratic Republic of the Congo” OR MH “Equatorial Guinea” OR MH “Gabon” OR MH “Burundi” OR MH “Djibouti” OR MH “Eritrea” OR MH “Ethiopia” OR MH “Kenya” OR MH “Rwanda” OR MH “Somalia” OR MH “Sudan” OR MH “Tanzania” OR MH “Uganda” OR MH “Angola” OR MH “Botswana” OR MH “Lesotho” OR MH “Malawi” OR MH “Mozambique” OR MH “Swaziland” OR MH “Zambia” OR MH “Zimbabwe” OR MH “Benin” OR MH “Burkina Faso” OR MH “Cape Verde” OR MH “Cote d’Ivoire” OR MH “Gambia” OR MH “Ghana” OR MH “Guinea” OR MH “Guinea-Bissau” OR MH “Liberia” OR MH “Mali” OR MH “Mauritania” OR MH “Niger” OR MH “Nigeria” OR MH “Senegal” OR MH “Sierra Leone” OR MH “Togo” OR MH “West Indies” OR MH “Cuba” OR MH “Dominica” OR MH “Dominican Republic” OR MH “Haiti” OR MH “Jamaica” OR MH “Martinique” OR MH “Central America” OR MH “Belize” OR MH “Costa Rica” OR MH “El Salvador” OR MH “Guatemala” OR MH “Honduras” OR MH “Nicaragua” OR MH “Panama” OR MH “Latin America” OR MH “Mexico” OR MH “South America” OR MH “Argentina” OR MH “Bolivia” OR MH “Brazil” OR MH “Colombia” OR MH “Ecuador” OR MH “French Guiana” OR MH “Guyana” OR MH “Paraguay” OR MH “Peru” OR MH “Suriname” OR MH “Venezuela” OR MH “Asia” OR MH “Asia, Western” OR MH “Asia, Southeastern” OR MH “Asia, Central” OR MH “Kazakhstan” OR MH “Kyrgyzstan” OR MH “Tajikistan” OR MH “Turkmenistan” OR MH “Uzbekistan” OR MH “Borneo” OR MH “Cambodia” OR MH “East Timor” OR MH “Indonesia” OR MH “Laos” OR MH “Malaysia” OR MH “Myanmar” OR MH “Philippines” OR MH “Thailand” OR MH “Timor” OR MH “Vietnam” OR MH “Bangladesh” OR MH “Bhutan” OR MH “India” OR MH “Middle East” OR MH “Afghanistan” OR MH “Iran” OR MH “Iraq” OR MH “Jordan” OR MH “Lebanon” OR MH “Syria” OR MH “Turkey” OR MH “Yemen” OR MH “Nepal” OR MH “Pakistan” OR MH “Sri Lanka” OR MH “Far East” OR MH “China” OR MH “Mongolia” OR MH “North Korea” OR MH “Indian Ocean Islands” OR MH “Madagascar” OR MH “Pacific Islands” OR MH “Melanesia” OR MH “Micronesia” OR MH “Polynesia” OR MH “Papua New Guinea” OR MH “Samoa” OR MH “American Samoa” OR MH “Independent State of Samoa” OR MH “Romania” OR MH “Russia” OR MH “USSR” OR MH “Croatia” or MH “Albania” or MH “Bosnia-Herzegovina” or MH “Bulgaria” or MH “Byelarus” or MH “Macedonia (Republic)” or MH “Moldova” or MH “Serbia” or MH “Ukraine” or MH “Yugoslavia” or MH “Armenia” or MH “Azerbaijan” or MH “Georgia (republic)” ) ) OR ( (Afghanistan* or Albania* or Algeria* or Angola* or Argentina* or Armenia* or Azerbaijan* or Bangladesh* or Beliz* or Benin* or Bhutan* or Bolivia* or Bosnia* or Herzegovin* or Botswan* or Brazil* or Bulgaria* or Burkina* or Burundi* or “Cabo Verde*” or “Cape Verde*” or Cambodia* or Cameroon* or Chad* or China or Chinese or Colombia* or Comor* or Congo* or “Costa Rica*” or “Cote d'Ivoir*” or “Ivory Coast” or Cuba* or Djibouti* or Dominica* or Ecuador* or Egypt* or “El Salvador*” or Eritrea* or Ethiopia* or Fiji* or Gabon* or Gambia* or Georgia* or Ghana* or Grenad* or Guatemala* or Guinea* or Guyan* or Haiti* or Hondura* or Hungar* or India* or Indonesia* or Iran* or Iraq* or Jamaica* or Jordan* or Kazakhstan* or Kenya* or Kiribati* or Korea* or Kosov* or Kyrgyz* or Lao* or Leban* or Lesotho* or Liberia* or Libya* or Macedonia* or Madagascar* or Malawi* or Malaysia* or Maldiv* or Mali* or “Marshall Island*” or Mauritania* or Mauriti* or Mexic* or Moldova* or Mongolia* or Montenegr* or Morocc* or Mozambi* or Myanma* or Burmese or Namibia* or Nepal* or Nicaragua* or Niger* or Pakistan* or Palau* or Panama* or “Papua New Guinea*” or Paraguay* or Peru* or Philippines or Filipino or Romania* or Rwanda* or Samoa* or “Sao Tome*” or Senegal* or Serbia* or Seychell* or “Sierra Leon*” or “Solomon Island*” or Somalia* or Sudan* or “Sri Lanka*” or “St Lucia*” or “Saint Lucia” or “St Vincent” or “Saint Vincent” or Grenadines or Surinam* or Swazi* or Syria* or Tajikistan* or Tanzania* or Thai* or Timor* or Togo* or Tonga* or Tunisia* or Turk* or Tuvalu* or Uganda* or Ukrain* or Uzbekistan* or Vanuatu* or Venezuela* or Vietnam* or “Viet-Nam*” or “West Bank” or Gaza or Yemen* or Zambia* or Zimbabwe* or Russia* or Croatia* or Nauru* or Yugoslavia* or USSR or Soviet* or Byelarus* or Belarus*) ) OR ( (africa* or asia* or caribbean or "central america*" or "latin america*" or "south america*" or melanesia* or micronesia* or polynesia*) ) |
| S17 | ( (“resource-limit*” or “resource-poor” or “low-resource*” or “limited-resource*” or “resource-constrain*” or “constrain*-resource*” or “under-resource*” or “poor*-resource*” or “resource-scarce*” or “scarce*-resource*” or “low-income” or “middle-income” or lowincome or middleincome or (low* N2 “middle-income”)) ) OR ( ((developing or underdeveloped or “under-developed” or emerging or “less-developed” or “least-developed” or “less-economically developed” or “least-economically developed” or “less-affluent” or “least-affluent” or “least-industriali#ed” or “non-industriali#ed” or deprived or poor) N0 (country or countries or nation? or region? or economy or economies)) ) OR ( ((developing or underdeveloped or “under-developed” or “less-developed” or “least-developed”) N0 (population* or world)) ) |
| S18 | ( (“third-world*” or thirdworld* or “3rd-world*” or lmic or lmics or “lami countr*” or “lalmi countr*” or “transitional countr*”) ) OR ( (low* N0 (gdp or gnp or "gross domestic" or "gross national")) ) OR ( ((underserved or "under-served") N0 (countr* or nation? or population*)) ) |
| S19 | S16 OR S17 OR S18 |
| S20 | S7 AND S11 AND S19 |
| S21 | S7 AND S15 AND S19 |
| S22 | ( (MH "Antiinflammatory Agents, Non-Steroidal+") OR (MH "Cox-2 Inhibitors") ) OR (MH "Tranexamic Acid") OR ( (MH "Hormonal Contraception") OR (MH "Contraceptive Agents, Hormonal+") OR (MH "Contraceptives, Oral Combined") ) OR (MH "Intrauterine Devices") OR (MH "Progestational Hormones+") |
| S23 | ( ("non-steroid* antiinflammatory" or "non-steroid* anti-inflammatory" or "nonsteroid antiinflammatory" or "nonsteroid* anti-inflammatory" or NSAID or NSAIDs) ) OR ( (“cyclooxygenase inhibitor*” or “cyclo-oxygenase inhibitor*” or “prostaglandin synthetase inhibitor*” or “cyclooxygenase-2 inhibitor*” or “COX-2 inhibitor*” or “COX-2 specific inhibitor*” or “COX2 inhibitor*” or “COX2 specific inhibitor*” or coxib or coxibs) ) OR ( (antifibrinolytic* or “fibrinolysis inhibitor*” or “aminomethyl cyclohexane carboxylic acid*” or “aminomethyl cyclohexanecarboxylic acid*” or “aminomethylcyclohexane carbonic acid*” or “aminomethylcyclohexane carboxylic acid*” or “aminomethylcyclohexanecarbonic acid*” or “aminomethylcyclohexanecarboxylic acid*” or “aminomethylcyclohexanocarboxylic acid*” or “aminomethylcyclohexanoic acid*” or “tranexamic acid*” or tranexam or “tranexanic acid*” or “transexamic acid*” or traxamic or TXA) ) OR ( ("combined oral contraceptive*" or "combined hormonal contraceptive*" or "combined oral hormonal contraceptive*" or "combined vaginal ring*" or combined hormonal vaginal ring* or contraceptive vaginal ring*) ) OR ( ( "luteal oral progestogen*" or "LNG-IUS" or "LNG-IUD" ) OR ( "levonorgestrel IUD*" or "levonorgestrel releasing intrauterine" or "levonorgestrel releasing IUD" or "levonorgestrel releasing intra-uterine" or "levonorgestrel intrauterine system" or "levonorgestrel intrauterine device" ) ) OR ( (gestogen* or progestagen* or progestin* or progestogen*) ) |
| S24 | S22 OR S23 |
| S25 | S7 AND S24 |
| S26 | S20 OR S21 OR S25 |
| S27 | S20 OR S21 OR S25  Limiters - Published Date: 20000101-20231231 |
| S28 | (((MH "Animals+") OR (MH "Animal Studies") OR (TI "animal model*")) NOT (MH "human")) |
| S29 | S27 NOT S28 |
| S30 | PT Commentary or Doctoral Dissertation or Editorial or Letter or Masters Thesis |
| **S31** | **S29 NOT S30** |

Bottom of Form

**New records since June 2023**

| **S32** | **Limiters - Publication Date: 2023-2024** |
| --- | --- |

**DATABASE: SCOPUS**

( ( ( ( TITLE-ABS-KEY ( ( meno?rhagi* OR menorr?agi* OR menor*agi* OR hypermeno?rhea* OR hypermenorr?oea* OR hypermeno?rh?ea* OR meno?rhagy OR menorr?agy OR polymenor?hea* OR polymenorr?ea OR polymeno?rh?ea OR menometro?rhagi* OR menometrorr?agi* OR menometror*agi? OR "Menstrual morbidity" ) ) OR TITLE-ABS-KEY ( ( ( ( severe OR severity OR massive* OR excessive* OR extensive* OR "large scale" OR acute OR unrelenting OR overwhelming OR uncontrolled OR extreme OR significant OR heavy OR debilitat* OR prolonged OR excess OR dysfunctional OR abnormal ) W/2 ( bleed* OR "blood loss*" OR "blood flow*" OR h?emo*rhag* ) ) AND ( menstrua* OR menses OR catamenia OR menstruum ) ) ) ) ) OR ( ( TITLE-ABS-KEY ( ( ( heavy OR massive* OR excessive* OR extensive* OR debilitat* OR prolonged OR dysfunctional OR abnormal OR severe ) W/0 ( menstrua* OR menses OR catamenia OR menstruum ) ) ) OR TITLE-ABS-KEY ( ( heavy W/0 ( period? OR mbl ) ) ) OR TITLE-ABS-KEY ( ( ( "long* duration" OR "excessive duration" OR "prolonged duration" ) W/1 ( menstrua* OR menses OR catamenia OR menstruum ) ) ) ) ) OR ( ( TITLE-ABS-KEY ( ( ( suffer* OR disabl* OR debilitat* OR burden* OR impact* ) W/2 hmb ) ) OR TITLE-ABS-KEY ( ( ( bleeding OR "blood loss" ) W/2 ( score? OR pattern? OR measur* OR assess* OR amount? OR evaluat* ) W/2 ( menstrua* OR menses OR catamenia OR menstruum OR mbl ) ) ) ) ) OR ( ( TITLE-ABS-KEY ( ( hmb W/1 ( aub OR mbl OR dub ) ) ) OR TITLE-ABS-KEY ( ( ( abnormal OR excessive ) W/0 ( "uterine bleeding" OR "uterine blood loss" ) ) ) OR TITLE-ABS-KEY ( ( ( "ovulatory disorder*" OR adenomyosis OR endometrios* OR "endometrial polyp?" OR "endometrial hyperplasia" OR "uterine fibroid?" OR leiomyomata ) AND ( ( severe OR severity OR massive* OR excessive* OR extensive* OR "large scale" OR acute OR unrelenting OR overwhelming OR uncontrolled OR extreme OR significant OR heavy OR debilitat* OR prolonged OR excess OR dysfunctional OR abnormal ) W/2 ( bleed* OR "blood loss*" OR "blood flow*" OR h?emo*rhag* ) ) ) ) ) ) ) AND ( ( ( TITLE-ABS-KEY ( ( qol OR hrqol OR daly OR qaly OR "quality of life" OR "life quality" OR experience* OR burden* ) ) OR TITLE-ABS-KEY ( ( "living day to day" OR "lives of" OR "living with" OR "normal life" OR "normal lives" OR ( ( "every day" OR everyday OR daily ) W/0 ( life OR living OR routine* OR functioning OR activit* OR interest? ) ) OR ( ( realities OR difficulties ) W/1 ( life OR living ) ) OR ( "life quality" OR "life satisfaction" OR "satisf* with life" ) OR ( "school life" OR "work life" OR "social life" OR "family life" OR "future life" OR "future lives" ) ) ) ) ) OR ( ( TITLE-ABS-KEY ( ( ( impact* OR affect* OR difficulties OR problems OR dysfunction* OR disrupt* OR disturb* OR implication* OR implications OR concerns OR worries OR worrying OR adjustment* ) W/2 ( physical OR disabilit* OR disabl* OR abilit* OR life OR living OR lives OR functioning OR activit* ) ) ) OR TITLE-ABS-KEY ( ( ( impact* OR interfere* OR affect* OR difficulties OR problems OR dysfunction* OR disordered OR disrupt* OR disturb* OR implications OR concerns OR worries OR worrying OR adjustment ) W/2 ( studying OR school OR training OR learning OR employment OR work OR job OR jobs OR performance OR studies OR occupation* OR responsibilit* OR roles OR future* ) ) ) ) ) ) AND ( ( ( TITLE-ABS-KEY ( ( ( developing OR underdeveloped OR "under-developed" OR "less-developed" OR "least-developed" ) W/0 ( population* OR world ) ) ) OR TITLE-ABS-KEY ( ( "third-world*" OR thirdworld* OR "3rd-world*" OR lmic OR lmics OR "lami countr*" OR "lalmi countr*" OR "transitional countr*" ) ) OR TITLE-ABS-KEY ( ( low* W/0 ( gdp OR gnp OR "gross domestic" OR "gross national" ) ) ) OR TITLE-ABS-KEY ( ( ( underserved OR "under-served" ) W/0 ( countr* OR nation? OR population* ) ) ) ) ) OR ( ( TITLE-ABS-KEY ( ( "resource-limit*" OR "resource-poor" OR "low-resource*" OR "limited-resource*" OR "resource-constrain*" OR "constrain*-resource*" OR "under-resource*" OR "poor*-resource*" OR "resource-scarce*" OR "scarce*-resource*" OR "low-income" OR "middle-income" OR lowincome OR middleincome OR ( low* W/2 "middle-income" ) ) ) OR TITLE-ABS-KEY ( ( ( developing OR underdeveloped OR "under-developed" OR emerging OR "less-developed" OR "least-developed" OR "less-economically developed" OR "least-economically developed" OR "less-affluent" OR "least-affluent" OR "least-industriali?ed" OR "non-industriali?ed" OR deprived OR poor ) W/0 ( country OR countries OR nation? OR region? OR economy OR economies ) ) ) ) ) OR ( TITLE-ABS-KEY ( ( afghanistan* OR albania* OR algeria* OR angola* OR argentina* OR armenia* OR azerbaijan* OR bangladesh* OR beliz* OR benin* OR bhutan* OR bolivia* OR bosnia* OR herzegovin* OR botswan* OR brazil* OR bulgaria* OR burkina* OR burundi* OR "Cabo Verde*" OR "Cape Verde*" OR cambodia* OR cameroon* OR chad* OR china OR chinese OR colombia* OR comor* OR congo* OR "Costa Rica*" OR "Cote d'Ivoir*" OR "Ivory Coast" OR cuba* OR djibouti* OR dominica* OR ecuador* OR egypt* OR "El Salvador*" OR eritrea* OR ethiopia* OR fiji* OR gabon* OR gambia* OR georgia* OR ghana* OR grenad* OR guatemala* OR guinea* OR guyan* OR haiti* OR hondura* OR hungar* OR india* OR indonesia* OR iran* OR iraq* OR jamaica* OR jordan* OR kazakhstan* OR kenya* OR kiribati* OR korea* OR kosov* OR kyrgyz* OR lao* OR leban* OR lesotho* OR liberia* OR libya* OR macedonia* OR madagascar* OR malawi* OR malaysia* OR maldiv* OR mali* OR "Marshall Island*" OR mauritania* OR mauriti* OR mexic* OR moldova* OR mongolia* OR montenegr* OR morocc* OR mozambi* OR myanma* OR burmese OR namibia* OR nepal* OR nicaragua* OR niger* OR pakistan* OR palau* OR panama* OR "Papua New Guinea*" OR paraguay* OR peru* OR philippines OR filipino OR romania* OR rwanda* OR samoa* OR "Sao Tome*" OR senegal* OR serbia* OR seychell* OR "Sierra Leon*" OR "Solomon Island*" OR somalia* OR sudan* OR "Sri Lanka*" OR "St Lucia*" OR "Saint Lucia" OR "St Vincent" OR "Saint Vincent" OR grenadines OR surinam* OR swazi* OR syria* OR tajikistan* OR tanzania* OR thai* OR timor* OR togo* OR tonga* OR tunisia* OR turk* OR tuvalu* OR uganda* OR ukrain* OR uzbekistan* OR vanuatu* OR venezuela* OR vietnam* OR "Viet-Nam*" OR "West Bank" OR gaza OR yemen* OR zambia* OR zimbabwe* OR russia* OR croatia* OR nauru* OR yugoslavia* OR ussr OR soviet* OR byelarus* OR belarus* ) ) ) ) ) OR ( ( ( ( TITLE-ABS-KEY ( ( meno?rhagi* OR menorr?agi* OR menor*agi* OR hypermeno?rhea* OR hypermenorr?oea* OR hypermeno?rh?ea* OR meno?rhagy OR menorr?agy OR polymenor?hea* OR polymenorr?ea OR polymeno?rh?ea OR menometro?rhagi* OR menometrorr?agi* OR menometror*agi? OR "Menstrual morbidity" ) ) OR TITLE-ABS-KEY ( ( ( ( severe OR severity OR massive* OR excessive* OR extensive* OR "large scale" OR acute OR unrelenting OR overwhelming OR uncontrolled OR extreme OR significant OR heavy OR debilitat* OR prolonged OR excess OR dysfunctional OR abnormal ) W/2 ( bleed* OR "blood loss*" OR "blood flow*" OR h?emo*rhag* ) ) AND ( menstrua* OR menses OR catamenia OR menstruum ) ) ) ) ) OR ( ( TITLE-ABS-KEY ( ( ( heavy OR massive* OR excessive* OR extensive* OR debilitat* OR prolonged OR dysfunctional OR abnormal OR severe ) W/0 ( menstrua* OR menses OR catamenia OR menstruum ) ) ) OR TITLE-ABS-KEY ( ( heavy W/0 ( period? OR mbl ) ) ) OR TITLE-ABS-KEY ( ( ( "long* duration" OR "excessive duration" OR "prolonged duration" ) W/1 ( menstrua* OR menses OR catamenia OR menstruum ) ) ) ) ) OR ( ( TITLE-ABS-KEY ( ( ( suffer* OR disabl* OR debilitat* OR burden* OR impact* ) W/2 hmb ) ) OR TITLE-ABS-KEY ( ( ( bleeding OR "blood loss" ) W/2 ( score? OR pattern? OR measur* OR assess* OR amount? OR evaluat* ) W/2 ( menstrua* OR menses OR catamenia OR menstruum OR mbl ) ) ) ) ) OR ( ( TITLE-ABS-KEY ( ( hmb W/1 ( aub OR mbl OR dub ) ) ) OR TITLE-ABS-KEY ( ( ( abnormal OR excessive ) W/0 ( "uterine bleeding" OR "uterine blood loss" ) ) ) OR TITLE-ABS-KEY ( ( ( "ovulatory disorder*" OR adenomyosis OR endometrios* OR "endometrial polyp?" OR "endometrial hyperplasia" OR "uterine fibroid?" OR leiomyomata ) AND ( ( severe OR severity OR massive* OR excessive* OR extensive* OR "large scale" OR acute OR unrelenting OR overwhelming OR uncontrolled OR extreme OR significant OR heavy OR debilitat* OR prolonged OR excess OR dysfunctional OR abnormal ) W/2 ( bleed* OR "blood loss*" OR "blood flow*" OR h?emo*rhag* ) ) ) ) ) ) ) AND ( ( ( TITLE-ABS-KEY ( ( access* OR need* OR seek* OR accept* OR prefer* OR trust* OR encounter* OR understand OR satisfaction OR dissatisfaction OR acceptable OR perceive* OR perception* OR expectation* OR "wait* time*" OR "wait* list*" OR timeliness OR delay* ) ) OR TITLE-ABS-KEY ( ( price* OR pricing OR fund* OR availability OR available OR cost* OR monetary OR budget* OR financ* OR reimburs* OR payer* OR payee OR investment* OR access OR accessibility OR accessible* OR supply OR market* OR affordability OR affordable* OR quality OR "pre-qualified" OR "off-label" OR inequit* OR inequalit* ) ) OR TITLE-ABS-KEY ( ( disbursement? OR expenditure OR expense? OR expensive* OR remittance OR recompens* OR repayment? OR refund* OR recoup* OR rebate? OR compensate? OR compensation OR prepay* OR overpay* OR underpay* ) ) ) ) OR ( ( TITLE-ABS-KEY ( ( barrier* OR facilitat* OR enable* OR enabling OR challenge* OR attitude* OR belief* OR views* OR behavio?r* OR influenc* OR adopt* OR uptake OR eligibility OR eligible OR value* OR economic* OR discount* OR payment* OR provision* ) ) OR TITLE-ABS-KEY ( ( repurposed OR "universal health coverage" OR "stringent regulatory authorit*" OR "national regulatory authorit*" OR procurement OR registration OR infrastructure OR remuneration ) ) OR TITLE-ABS-KEY ( ( safety OR efficacy ) ) ) ) OR ( ( TITLE-ABS-KEY ( ( ( restrict* OR limit* ) AND ( distribut* OR prescri* OR dispens* ) ) ) OR TITLE-ABS-KEY ( ( ( counterfeit OR fake OR false OR falsified ) W/0 ( drug* OR medicine* OR medication* ) ) ) ) ) ) AND ( ( ( TITLE-ABS-KEY ( ( ( developing OR underdeveloped OR "under-developed" OR "less-developed" OR "least-developed" ) W/0 ( population* OR world ) ) ) OR TITLE-ABS-KEY ( ( "third-world*" OR thirdworld* OR "3rd-world*" OR lmic OR lmics OR "lami countr*" OR "lalmi countr*" OR "transitional countr*" ) ) OR TITLE-ABS-KEY ( ( low* W/0 ( gdp OR gnp OR "gross domestic" OR "gross national" ) ) ) OR TITLE-ABS-KEY ( ( ( underserved OR "under-served" ) W/0 ( countr* OR nation? OR population* ) ) ) ) ) OR ( ( TITLE-ABS-KEY ( ( "resource-limit*" OR "resource-poor" OR "low-resource*" OR "limited-resource*" OR "resource-constrain*" OR "constrain*-resource*" OR "under-resource*" OR "poor*-resource*" OR "resource-scarce*" OR "scarce*-resource*" OR "low-income" OR "middle-income" OR lowincome OR middleincome OR ( low* W/2 "middle-income" ) ) ) OR TITLE-ABS-KEY ( ( ( developing OR underdeveloped OR "under-developed" OR emerging OR "less-developed" OR "least-developed" OR "less-economically developed" OR "least-economically developed" OR "less-affluent" OR "least-affluent" OR "least-industriali?ed" OR "non-industriali?ed" OR deprived OR poor ) W/0 ( country OR countries OR nation? OR region? OR economy OR economies ) ) ) ) ) OR ( TITLE-ABS-KEY ( ( afghanistan* OR albania* OR algeria* OR angola* OR argentina* OR armenia* OR azerbaijan* OR bangladesh* OR beliz* OR benin* OR bhutan* OR bolivia* OR bosnia* OR herzegovin* OR botswan* OR brazil* OR bulgaria* OR burkina* OR burundi* OR "Cabo Verde*" OR "Cape Verde*" OR cambodia* OR cameroon* OR chad* OR china OR chinese OR colombia* OR comor* OR congo* OR "Costa Rica*" OR "Cote d'Ivoir*" OR "Ivory Coast" OR cuba* OR djibouti* OR dominica* OR ecuador* OR egypt* OR "El Salvador*" OR eritrea* OR ethiopia* OR fiji* OR gabon* OR gambia* OR georgia* OR ghana* OR grenad* OR guatemala* OR guinea* OR guyan* OR haiti* OR hondura* OR hungar* OR india* OR indonesia* OR iran* OR iraq* OR jamaica* OR jordan* OR kazakhstan* OR kenya* OR kiribati* OR korea* OR kosov* OR kyrgyz* OR lao* OR leban* OR lesotho* OR liberia* OR libya* OR macedonia* OR madagascar* OR malawi* OR malaysia* OR maldiv* OR mali* OR "Marshall Island*" OR mauritania* OR mauriti* OR mexic* OR moldova* OR mongolia* OR montenegr* OR morocc* OR mozambi* OR myanma* OR burmese OR namibia* OR nepal* OR nicaragua* OR niger* OR pakistan* OR palau* OR panama* OR "Papua New Guinea*" OR paraguay* OR peru* OR philippines OR filipino OR romania* OR rwanda* OR samoa* OR "Sao Tome*" OR senegal* OR serbia* OR seychell* OR "Sierra Leon*" OR "Solomon Island*" OR somalia* OR sudan* OR "Sri Lanka*" OR "St Lucia*" OR "Saint Lucia" OR "St Vincent" OR "Saint Vincent" OR grenadines OR surinam* OR swazi* OR syria* OR tajikistan* OR tanzania* OR thai* OR timor* OR togo* OR tonga* OR tunisia* OR turk* OR tuvalu* OR uganda* OR ukrain* OR uzbekistan* OR vanuatu* OR venezuela* OR vietnam* OR "Viet-Nam*" OR "West Bank" OR gaza OR yemen* OR zambia* OR zimbabwe* OR russia* OR croatia* OR nauru* OR yugoslavia* OR ussr OR soviet* OR byelarus* OR belarus* ) ) ) ) ) OR ( ( ( ( TITLE-ABS-KEY ( ( meno?rhagi* OR menorr?agi* OR menor*agi* OR hypermeno?rhea* OR hypermenorr?oea* OR hypermeno?rh?ea* OR meno?rhagy OR menorr?agy OR polymenor?hea* OR polymenorr?ea OR polymeno?rh?ea OR menometro?rhagi* OR menometrorr?agi* OR menometror*agi? OR "Menstrual morbidity" ) ) OR TITLE-ABS-KEY ( ( ( ( severe OR severity OR massive* OR excessive* OR extensive* OR "large scale" OR acute OR unrelenting OR overwhelming OR uncontrolled OR extreme OR significant OR heavy OR debilitat* OR prolonged OR excess OR dysfunctional OR abnormal ) W/2 ( bleed* OR "blood loss*" OR "blood flow*" OR h?emo*rhag* ) ) AND ( menstrua* OR menses OR catamenia OR menstruum ) ) ) ) ) OR ( ( TITLE-ABS-KEY ( ( ( heavy OR massive* OR excessive* OR extensive* OR debilitat* OR prolonged OR dysfunctional OR abnormal OR severe ) W/0 ( menstrua* OR menses OR catamenia OR menstruum ) ) ) OR TITLE-ABS-KEY ( ( heavy W/0 ( period? OR mbl ) ) ) OR TITLE-ABS-KEY ( ( ( "long* duration" OR "excessive duration" OR "prolonged duration" ) W/1 ( menstrua* OR menses OR catamenia OR menstruum ) ) ) ) ) OR ( ( TITLE-ABS-KEY ( ( ( suffer* OR disabl* OR debilitat* OR burden* OR impact* ) W/2 hmb ) ) OR TITLE-ABS-KEY ( ( ( bleeding OR "blood loss" ) W/2 ( score? OR pattern? OR measur* OR assess* OR amount? OR evaluat* ) W/2 ( menstrua* OR menses OR catamenia OR menstruum OR mbl ) ) ) ) ) OR ( ( TITLE-ABS-KEY ( ( hmb W/1 ( aub OR mbl OR dub ) ) ) OR TITLE-ABS-KEY ( ( ( abnormal OR excessive ) W/0 ( "uterine bleeding" OR "uterine blood loss" ) ) ) OR TITLE-ABS-KEY ( ( ( "ovulatory disorder*" OR adenomyosis OR endometrios* OR "endometrial polyp?" OR "endometrial hyperplasia" OR "uterine fibroid?" OR leiomyomata ) AND ( ( severe OR severity OR massive* OR excessive* OR extensive* OR "large scale" OR acute OR unrelenting OR overwhelming OR uncontrolled OR extreme OR significant OR heavy OR debilitat* OR prolonged OR excess OR dysfunctional OR abnormal ) W/2 ( bleed* OR "blood loss*" OR "blood flow*" OR h?emo*rhag* ) ) ) ) ) ) ) AND ( ( ( TITLE-ABS-KEY ( ( "non-steroid* antiinflammatory" OR "non-steroid* anti-inflammatory" OR "nonsteroid antiinflammatory" OR "nonsteroid* anti-inflammatory" OR nsaid OR nsaids ) ) OR TITLE-ABS-KEY ( ( "cyclooxygenase inhibitor*" OR "cyclo-oxygenase inhibitor*" OR "prostaglandin synthetase inhibitor*" OR "cyclooxygenase-2 inhibitor*" OR "COX-2 inhibitor*" OR "COX-2 specific inhibitor*" OR "COX2 inhibitor*" OR "COX2 specific inhibitor*" OR coxib OR coxibs ) ) OR TITLE-ABS-KEY ( ( antifibrinolytic* OR "fibrinolysis inhibitor*" OR "aminomethyl cyclohexane carboxylic acid*" OR "aminomethyl cyclohexanecarboxylic acid*" OR "aminomethylcyclohexane carbonic acid*" OR "aminomethylcyclohexane carboxylic acid*" OR "aminomethylcyclohexanecarbonic acid*" OR "aminomethylcyclohexanecarboxylic acid*" OR "aminomethylcyclohexanocarboxylic acid*" OR "aminomethylcyclohexanoic acid*" OR "tranexamic acid*" OR tranexam OR "tranexanic acid*" OR "transexamic acid*" OR traxamic OR txa ) ) ) ) OR ( ( TITLE-ABS-KEY ( ( ( "combined oral contraceptive*" OR "combined hormonal contraceptive*" OR "combined oral hormonal contraceptive*" ) ) OR "combined vaginal ring*" ) OR TITLE-ABS-KEY ( ( "luteal oral progestogen*" OR "LNG-IUS" OR "LNG-IUD" ) OR ( "levonorgestrel IUD*" OR "levonorgestrel releasing intrauterine" ) ) OR TITLE-ABS-KEY ( ( gestogen* OR progestagen* OR progestin* OR progestogen* ) ) ) ) ) ) AND LIMIT TO DATE RANGE

AND ( LIMIT-TO ( PUBYEAR , 2023 ) OR LIMIT-TO ( PUBYEAR , 2022 ) OR LIMIT-TO ( PUBYEAR , 2021 ) OR LIMIT-TO ( PUBYEAR , 2020 ) OR LIMIT-TO ( PUBYEAR , 2019 ) OR LIMIT-TO ( PUBYEAR , 2018 ) OR LIMIT-TO ( PUBYEAR , 2017 ) OR LIMIT-TO ( PUBYEAR , 2016 ) OR LIMIT-TO ( PUBYEAR , 2015 ) OR LIMIT-TO ( PUBYEAR , 2014 ) OR LIMIT-TO ( PUBYEAR , 2013 ) OR LIMIT-TO ( PUBYEAR , 2012 ) OR LIMIT-TO ( PUBYEAR , 2011 ) OR LIMIT-TO ( PUBYEAR , 2010 ) OR LIMIT-TO ( PUBYEAR , 2009 ) OR LIMIT-TO ( PUBYEAR , 2008 ) OR LIMIT-TO ( PUBYEAR , 2007 ) OR LIMIT-TO ( PUBYEAR , 2006 ) OR LIMIT-TO ( PUBYEAR , 2005 ) OR LIMIT-TO ( PUBYEAR , 2004 ) OR LIMIT-TO ( PUBYEAR , 2003 ) OR LIMIT-TO ( PUBYEAR , 2002 ) OR LIMIT-TO ( PUBYEAR , 2001 ) OR LIMIT-TO ( PUBYEAR , 2000 ) )

AND ( EXCLUDE ( DOCTYPE , "le" ) OR EXCLUDE ( DOCTYPE , "ed" ) )

**= 4,249 document results**

**DATABASE: WEB OF SCIENCE**

#1

(meno?rhagi* or menorr?agi* or menor*agi* or hypermeno?rhea* or hypermenorr?oea* or hypermeno?rh?ea* or meno?rhagy or menorr?agy or polymenor?hea* or polymenorr?ea or polymeno?rh?ea or menometro?rhagi* or menometrorr?agi* or menometror*agi? or "Menstrual morbidity") (Topic) or (((severe or severity or massive* or excessive* or extensive* or “large scale” or acute or unrelenting or overwhelming or uncontrolled or extreme or significant or heavy or debilitat* or prolonged or excess or dysfunctional or abnormal) NEAR/2 (bleed* or “blood loss*” or “blood flow*” or h?emo*rhag*)) and (menstrua* or menses or catamenia or menstruum)) (Topic) or ((heavy or massive* or excessive* or extensive* or debilitat* or prolonged or dysfunctional or abnormal or severe) NEAR/0 (menstrua* or menses or catamenia or menstruum)) (Topic)

#2

(heavy NEAR/0 (period? or MBL)) (Topic) or (("long* duration" or "excessive duration" or "prolonged duration") NEAR/1 (menstrua* or menses or catamenia or menstruum)) (Topic) or ((suffer* or disabl* or debilitat* or burden* or impact*) NEAR/2 HMB) (Topic)

#3

((bleeding or “blood loss”) NEAR/2 (score? or pattern? or measur* or assess* or amount? or evaluat*) NEAR/2 (menstrua* or menses or catamenia or menstruum or MBL)) (Topic) or (HMB NEAR/1 (AUB or MBL or DUB)) (Topic)

#4

((abnormal or excessive) NEAR/0 ("uterine bleeding" or "uterine blood loss")) (Topic) or ((“ovulatory disorder*” or adenomyosis or endometrios* or “endometrial polyp?” or “endometrial hyperplasia” or “uterine fibroid?” or leiomyomata) and ((severe or severity or massive* or excessive* or extensive* or “large scale” or acute or unrelenting or overwhelming or uncontrolled or extreme or significant or heavy or debilitat* or prolonged or excess or dysfunctional or abnormal) NEAR/2 (bleed* or “blood loss*” or “blood flow*” or h?emo*rhag*))) (Topic)

#5

#1 OR #2 OR #3 OR #4

#6

(QOL or HRQOL or DALY or QALY or "quality of life" or "life quality" or experience* or burden*) (Topic) or ("living day to day" or "lives of" or "living with" or “normal life” or “normal lives” or ((“every day” or everyday or daily) NEAR/0 (life or living or routine* or functioning or activit* or interest?)) or ((realities or difficulties) NEAR/1 (life or living)) or (“life quality” or “life satisfaction” or "satisf* with life") or (“school life” or “work life” or “social life” or “family life” or “future life” or “future lives”)) (Topic)

#7

((impact* or affect* or difficulties or problems or dysfunction* or disrupt* or disturb* or implication* or implications or concerns or worries or worrying or adjustment*) NEAR/2 (physical or disabilit* or disabl* or abilit* or life or living or lives or functioning or activit*)) (Topic) or ((impact* or interfere* or affect* or difficulties or problems or dysfunction* or disordered or disrupt* or disturb* or implications or concerns or worries or worrying or adjustment) NEAR/2 (studying or school or training or learning or employment or work or job or jobs or performance or studies or occupation* or responsibilit* or roles or future*)) (Topic)

#8

#6 OR #7

#9

(cultural* or transcultural or ethnic* or racial* or stigma*) (Topic) or (access* or need* or seek* or accept* or prefer* or trust* or encounter* or understand or satisfaction or dissatisfaction or acceptable or perceive* or perception* or expectation* or "wait* time*" or "wait* list*" or timeliness or delay*) (Topic) or (price* or pricing or fund* or availability or available or cost* or monetary or budget* or financ* or reimburs* or payer* or payee or investment* or access or accessibility or accessible* or supply or market* or affordability or affordable* or quality or "pre-qualified" or "off-label" or inequit* or inequalit*) (Topic) or (disbursement? or expenditure or expense? or expensive* or remittance or recompens* or repayment? or refund* or recoup* or rebate? or compensate? or compensation or prepay* or overpay* or underpay*) (Topic)

#10

(Barrier* or facilitat* or enable* or enabling or challenge* or attitude* or belief* or views* or behavio?r* or influenc* or adopt* or uptake or eligibility or eligible or value* or economic* or discount* or payment* or provision*) (Topic) or (repurposed or "universal health coverage" or "stringent regulatory authorit*" or "national regulatory authorit*" or procurement or registration or infrastructure or remuneration) (Topic) or (safety or efficacy) (Topic) or ((restrict* or limit*) and (distribut* or prescri* or dispens*)) (Topic) or ((counterfeit or fake or false or falsified) N0 (drug* or medicine* or medication*)) (Topic)

#11

#9 OR #10

#12

(Afghanistan* or Albania* or Algeria* or Angola* or Argentina* or Armenia* or Azerbaijan* or Bangladesh* or Beliz* or Benin* or Bhutan* or Bolivia* or Bosnia* or Herzegovin* or Botswan* or Brazil* or Bulgaria* or Burkina* or Burundi* or “Cabo Verde*” or “Cape Verde*” or Cambodia* or Cameroon* or Chad* or China or Chinese or Colombia* or Comor* or Congo* or “Costa Rica*” or “Cote d'Ivoir*” or “Ivory Coast” or Cuba* or Djibouti* or Dominica* or Ecuador* or Egypt* or “El Salvador*” or Eritrea* or Ethiopia* or Fiji* or Gabon* or Gambia* or Georgia* or Ghana* or Grenad* or Guatemala* or Guinea* or Guyan* or Haiti* or Hondura* or Hungar* or India* or Indonesia* or Iran* or Iraq* or Jamaica* or Jordan* or Kazakhstan* or Kenya* or Kiribati* or Korea* or Kosov* or Kyrgyz* or Lao* or Leban* or Lesotho* or Liberia* or Libya* or Macedonia* or Madagascar* or Malawi* or Malaysia* or Maldiv* or Mali* or “Marshall Island*” or Mauritania* or Mauriti* or Mexic* or Moldova* or Mongolia* or Montenegr* or Morocc* or Mozambi* or Myanma* or Burmese or Namibia* or Nepal* or Nicaragua* or Niger* or Pakistan* or Palau* or Panama* or “Papua New Guinea*” or Paraguay* or Peru* or Philippines or Filipino or Romania* or Rwanda* or Samoa* or “Sao Tome*” or Senegal* or Serbia* or Seychell* or “Sierra Leon*” or “Solomon Island*” or Somalia* or Sudan* or “Sri Lanka*” or “St Lucia*” or “Saint Lucia” or “St Vincent” or “Saint Vincent” or Grenadines or Surinam* or Swazi* or Syria* or Tajikistan* or Tanzania* or Thai* or Timor* or Togo* or Tonga* or Tunisia* or Turk* or Tuvalu* or Uganda* or Ukrain* or Uzbekistan* or Vanuatu* or Venezuela* or Vietnam* or “Viet-Nam*” or “West Bank” or Gaza or Yemen* or Zambia* or Zimbabwe* or Russia* or Croatia* or Nauru* or Yugoslavia* or USSR or Soviet* or Byelarus* or Belarus*) (Topic) or (africa* or asia* or caribbean or "central america*" or "latin america*" or "south america*" or melanesia* or micronesia* or polynesia*) (Topic)

#13

(“resource-limit*” or “resource-poor” or “low-resource*” or “limited-resource*” or “resource-constrain*” or “constrain*-resource*” or “under-resource*” or “poor*-resource*” or “resource-scarce*” or “scarce*-resource*” or “low-income” or “middle-income” or lowincome or middleincome or (low* NEAR/2 “middle-income”)) (Topic) or ((developing or underdeveloped or “under-developed” or emerging or “less-developed” or “least-developed” or “less-economically developed” or “least-economically developed” or “less-affluent” or “least-affluent” or “least-industriali?ed” or “non-industriali?ed” or deprived or poor) NEAR/0 (country or countries or nation? or region? or economy or economies)) (Topic) or ((developing or underdeveloped or “under-developed” or “less-developed” or “least-developed”) NEAR/0 (population* or world)) (Topic)

#14

(“third-world*” or thirdworld* or “3rd-world*” or lmic or lmics or “lami countr*” or “lalmi countr*” or “transitional countr*”) (Topic) or (low* NEAR/0 (gdp or gnp or "gross domestic" or "gross national")) (Topic) or ((underserved or "under-served") NEAR/0 (countr* or nation? or population*)) (Topic)

#15

#12 OR #13 OR #14

#16

#5 AND #8 AND #15

#17

#5 AND #11 AND #15

#18

("non-steroid* antiinflammatory" or "non-steroid* anti-inflammatory" or "nonsteroid antiinflammatory" or "nonsteroid* anti-inflammatory" or NSAID or NSAIDs) (Topic) or (“cyclooxygenase inhibitor*” or “cyclo-oxygenase inhibitor*” or “prostaglandin synthetase inhibitor*” or “cyclooxygenase-2 inhibitor*” or “COX-2 inhibitor*” or “COX-2 specific inhibitor*” or “COX2 inhibitor*” or “COX2 specific inhibitor*” or coxib or coxibs) (Topic) or (antifibrinolytic* or “fibrinolysis inhibitor*” or “aminomethyl cyclohexane carboxylic acid*” or “aminomethyl cyclohexanecarboxylic acid*” or “aminomethylcyclohexane carbonic acid*” or “aminomethylcyclohexane carboxylic acid*” or “aminomethylcyclohexanecarbonic acid*” or “aminomethylcyclohexanecarboxylic acid*” or “aminomethylcyclohexanocarboxylic acid*” or “aminomethylcyclohexanoic acid*” or “tranexamic acid*” or tranexam or “tranexanic acid*” or “transexamic acid*” or traxamic or TXA) (Topic)

#19

( ("combined oral contraceptive*" or "combined hormonal contraceptive*" or "combined oral hormonal contraceptive*") ) OR "combined vaginal ring*" (Topic) or ( "luteal oral progestogen*" or "LNG-IUS" or "LNG-IUD" ) OR ( "levonorgestrel IUD*" or "levonorgestrel releasing intrauterine" ) (Topic) or (gestogen* or progestagen* or progestin* or progestogen*) (Topic)

#20

#18 OR #19

#21

#20 AND #5

#22

#16 OR #17 OR #21

= 2,445 RESULTS

Limit by years 2000 to 2023

= 2,224 results

Exclude Document Types: Meeting Abstract or Editorial Material or Letter.

**= 2,124 results**

**DATABASE: GLOBAL INDEX MEDICUS**

(menorrhagia or menorragia or hypermenorrhea or hypermenorrhoea or menorrhagy or menorragy or polymenorrhea or polymenorrea or polymenorrhoea or menometrorrhagia or menometrorragia)

("**heavy** menstrual bleeding" or “**heavy** menstrual blood loss” or "**heavy** menstruation" or "**excessive** menstruation" or "**extensive** menstruation" or "**debilitating** menstruation" or "**prolonged** menstruation" or "**abnormal** menstruation" or "**severe** menstruation" or "**excessive** menstrual bleeding" or "**extensive** menstrual bleeding" or "**debilitating** menstrual bleeding" or "**prolonged** menstrual bleeding" or "**dysfunctional** menstrual bleeding" or "**abnormal** menstrual bleeding" or "**severe** menstrual bleeding" or “**significant** menstrual bleeding” or “**abundant** menstrual bleeding” or “**profuse** menstrual bleeding” or “**excessively** long menstruation” or “**excessively** long menstrual bleeding” or “**disabling** menstrual bleeding” or "**excessive** menstrual blood loss" or "**extensive** menstrual blood loss" or “**disabling** menstrual blood loss” or "**abnormal** menstrual blood loss” or "**severe** menstrual blood loss” or “**significant** menstrual blood loss” or “**abundant** menstrual blood loss” or “**profuse** menstrual blood loss” or "**heavy** period" or "**heavy** MBL" or "**heavy** menstrual flow" or "**excessive** menstrual flow " or "**extensive** menstrual flow" or "**prolonged** menstrual flow" or “**disabling** menstrual flow” or "**abnormal** menstrual flow" or "**severe** menstrual flow" or “**significant** menstrual flow” or “**abundant** menstrual flow” or “**profuse** menstrual flow”)

("**abnormal** uterine bleeding" or "**abnormal** uterine blood loss" or "**excessive** uterine bleeding" or "**excessive** uterine blood loss")


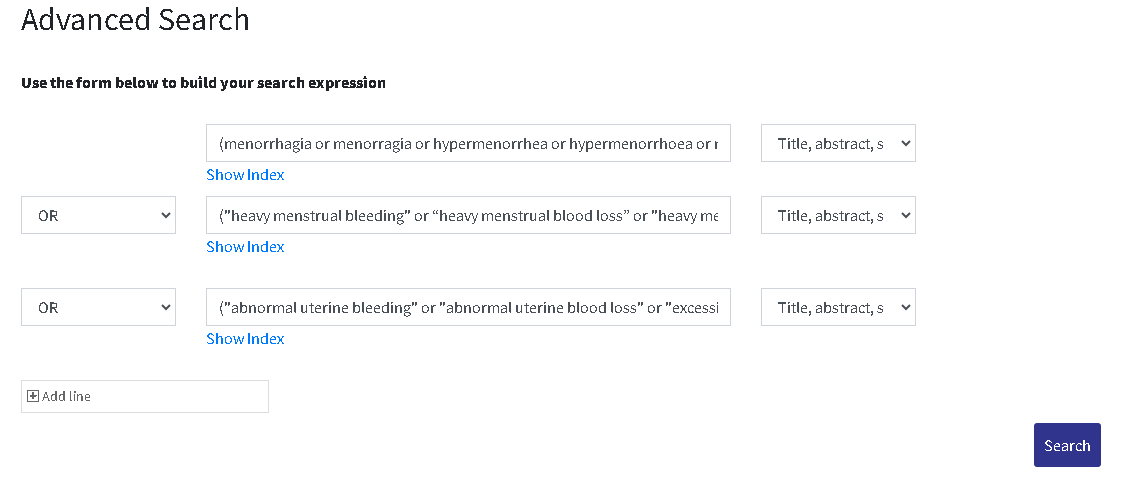


**1057 results for year range 2000 to 2023**
