# Supplementary material for: The prevalence and burden of heavy menstrual bleeding, and market access barriers of medical interventions with a focus on low- and middle-income countries: a scoping review
Source: BMC Womens Health. 2025 Nov 29;26:9. doi: 10.1186/s12905-025-04157-5 (PMC12771781; doi:10.1186/s12905-025-04157-5)
Supplement: Supplementary file 3 — Supplementary Material 3. [file 12905_2025_4157_MOESM3_ESM.docx]

**Supplement 3. References of included studies**

1. Abdali, K., M. Khajehei and H. Tabatabaee (2013). "The Effect of Mefenamic Acid and Naproxen on Heavy Menstrual Bleeding: A Placebo-Controlled Study." S. Afr. j. obstet. gynaecol 19(2): 31-34.
2. Abdel Malak, K. and O. Shawki (2006). "Management of menorrhagia with the levonorgestrel intrauterine system versus endometrial resection." Gynecological surgery 3(4): 275-280.
3. Abdollahi, N. G., M. Mirghafourvand and S. Mollazadeh (2018). "The effects of fennel on menstrual bleeding: A systematic review and meta-analysis." Journal of Complementary and Integrative Medicine 15(3): 20170154.
4. Abebe, M., G. Melaku, H. E. Hareru and T. M. Tebeje (2024). "Abnormal uterine bleeding and its associated factors among reproductive-age women who visit the gynecology ward in Dilla University General Hospital, Southern Ethiopia, 2022." BMC women's health 24(1): 281.
5. Abu Hashim, H., W. Alsherbini and M. Bazeed (2012). "Contraceptive vaginal ring treatment of heavy menstrual bleeding: a randomized controlled trial with norethisterone." Contraception 85(3): 246-252.
6. Abul, R., N. F. T. Selcuki, O. Karadeniz and P. Y. Bahat (2024). "A comparison of the effects of oral dydrogesterone and levonorgestrel-releasing intrauterine device on quality of life and sexual function in patients with abnormal uterine bleeding." Archives of gynecology and obstetrics 309(2): 715-719.
7. Adiguzel, C., S. Seyfettinoglu, D. Aka Satar, S. Arlier, E. Eskimez, F. Kaya and H. Nazik (2017). "Evaluation of quality of life and cost-effectiveness of definitive surgery and the levonorgestrel intrauterine system as treatment options for heavy menstrual bleeding." Turkish journal of medical sciences 47(3): 789-794.
8. Adwani, N. V. and R. M. Paramkush (2012). "A Study on Ayurveda Poly Herbal Compound of Yogaratnakar (17 - A.D.) w.s.r. Rakta-Pradara (Abnormal Uterine Bleeding)." INTERNATIONAL JOURNAL OF AYURVEDIC MEDICINE 3(4): 222-231.
9. Afroze, C. A., M. N. Ahmed, R. Jahan and M. Rahmatullah (2022). "Evaluation of herbal ingredients used in an ethno-polyherbal formulation for treating menorrhagia and dysmenorrhea in Bangladesh." Phytomedicine Plus 2(4).
10. Agarwal, M., S. Singh, Sr., S. Sinha and H. H. Sinha (2023). "Comparison of Bleeding Pattern and Quality of Life Before and After the Insertion of a Levonorgestrel Intrauterine System for Heavy Menstrual Bleeding: A Seven-Year Review." Cureus 15(3): e36142.
11. Agarwal, N., M. Gupta, A. Kriplani, N. Bhatla and N. Singh (2016). "Comparison of combined hormonal vaginal ring with ultralow-dose combined oral contraceptive pills in the management of heavy menstrual bleeding: A pilot study." Journal of obstetrics and gynaecology : the journal of the Institute of Obstetrics and Gynaecology 36(1): 71-75.
12. Agarwal, N. and A. Kriplani (2001). "Medical management of dysfunctional uterine bleeding." International journal of gynaecology and obstetrics: the official organ of the International Federation of Gynaecology and Obstetrics 75(2): 199-201.
13. Agarwal, P., A. Rani and S. Agrawal (2024). "Role of Centchroman (Ormeloxifene) in Dysfunctional Uterine Bleeding." International Journal of Pharmaceutical and Clinical Research 16(5): 1207-1215.
14. Ahmed, G. S. and A. M. M. Lotfy (2024). "Dietary pattern and menstrual disorders among female university students." International journal of adolescent medicine and health.
15. Akumu, L. A., S. N. Ratemo Bpharm and P. Owende (2021). "Assessment and management of women with heavy menstrual bleeding in Kenya: a best practice implementation project." JBI evidence implementation 19(2): 190-197.
16. Al-Hendy, A., L. Bradley, C. D. Owens, H. Wang, K. T. Barnhart, E. Feinberg, W. D. Schlaff, E. E. Puscheck, A. Wang, V. Gillispie, S. Hurtado, O. Muneyyirci-Delale, D. F. Archer, B. R. Carr, J. A. Simon and E. A. Stewart (2020). "Predictors of Response for Elagolix with Add-back Therapy in Women with Heavy Menstrual Bleeding Associated with Uterine Fibroids." American journal of obstetrics and gynecology.
17. Al-Hendy, A., L. Bradley, C. D. Owens, H. Wang, K. T. Barnhart, E. Feinberg, W. D. Schlaff, E. E. Puscheck, A. Wang, V. Gillispie, S. Hurtado, O. Muneyyirci-Delale, D. F. Archer, B. R. Carr, J. A. Simon and E. A. Stewart (2021). "Predictors of response for elagolix with add-back therapy in women with heavy menstrual bleeding associated with uterine fibroids." American journal of obstetrics and gynecology 224(1): 72.e71-72.e50.
18. Al-Hendy, A., R. Venturella, J. C. Arjona Ferreira, Y. Li, G. Soulban, R. B. Wagman and A. S. Lukes (2023). "LIBERTY randomized withdrawal study: relugolix combination therapy for heavy menstrual bleeding associated with uterine fibroids." American journal of obstetrics and gynecology 229(6): 662.e661-662.e625.
19. Ali, M., A. K. Hira, H. Jawaid, F. Zakaria and Z. Somjee (2021). "Safety and efficacy of elagolix (with and without add-back therapy) for the treatment of heavy menstrual bleeding associated with uterine leiomyomas: a systematic review and meta-analysis." Middle East Fertility Society Journal 26(1): 20.
20. Ali, M. K., R. S. Hussein, K. S. Abdallah and A. A. Mohamed (2024). "The use of dienogest in treatment of symptomatic adenomyosis: A systematic review and meta-analysis." Journal of gynecology obstetrics and human reproduction 53(7): 102795.
21. Altay, M. M. and A. Haberal (2008). "Abnormal uterine bleeding in adolescents: Treatment with combined oral contraceptive pills is effective even in hospitalized patients with bleeding disorders." Turkish journal of medical sciences 38(5): 431-435.
22. Ammerman, S. R. and A. L. Nelson (2013). "A new progestogen-only medical therapy for outpatient management of acute, abnormal uterine bleeding: a pilot study." American journal of obstetrics and gynecology 208(6): 499.e491-495.
23. Amu, E. O. and J. O. Bamidele (2014). "Prevalence of menstrual disorders among adolescent girls in Osogbo, South Western Nigeria." International journal of adolescent medicine and health 26(1): 101-106.
24. Anipindi, G. and I. Vani (2017). "Role of levonorgestrel releasing intrauterine device in management of heavy menstrual bleeding: A safe and effective option for all PALM COEIN variants." 4.
25. Anupam and M. Singh (2022). "A Retrospective Socio-Demographic Assessment of the Aspects of Adolescent Girls Having Menstrual Problems as Well as Type of Menstrual Problems." International Journal of Pharmaceutical and Clinical Research 14(5): 656-661.
26. Arathy, R., S. Pillai, N. S. Sreedevi, R. Aravindakshan, G. Rajmohan and S. J. Chandy (2021). "Effectiveness and safety of ormeloxifene and medroxyprogesterone acetate in dysfunctional uterine bleeding - A prospective interventional quasirandomized interval clinical study." National Journal of Physiology, Pharmacy and Pharmacology 11(3): 246-252.
27. Archer, D. F., E. A. Stewart, R. I. Jain, R. A. Feldman, A. S. Lukes, J. D. North, A. M. Soliman, J. Gao, J. W. Ng and K. Chwalisz (2017). "Elagolix for the management of heavy menstrual bleeding associated with uterine fibroids: results from a phase 2a proof-of-concept study." Fertility and sterility 108(1): 152-160.e154.
28. Ashraf, M. N., A. Habib-Ur-Rehman, Z. Shehzad, S. D. AlSharari and G. Murtaza (2017). "Clinical efficacy of levonorgestrel and norethisterone for the treatment of chronic abnormal uterine bleeding." JPMA. The Journal of the Pakistan Medical Association 67(9): 1331-1338.
29. Aslam, N., S. Blunt and P. Latthe (2010). "Effectiveness and tolerability of levonorgestrel intrauterine system in adolescents." Journal of obstetrics and gynaecology : the journal of the Institute of Obstetrics and Gynaecology 30(5): 489-491.
30. Asumah, M. N., Q. E. S. Adnani, E. K. Dzantor, M. A. Beig, G. M. Wuffele, D. R. Donkor, A. M. Abdulai, W. K. Azanu, A. D. Parsa, R. Kabir and A. Abubakari (2023). "Menstruation-Related School Absenteeism: An Urban Centre Study in the Northern Region of Ghana." WOMEN 3(4): 497-507.
31. Atak, Z., S. R. Ocakoglu and G. Ocakoglu (2023). "Levonorgestrel-releasing intrauterine device to treat abnormal uterine bleeding; not one treatment option fits all." Journal of the Turkish-German Gynecological Association 24(4): 246-251.
32. Ayik, M., M. Ingec, N. K. Ustyo and C. Gundogdud (2015). "17beta estradiol/norethisterone acetate and estradiol valerate/norgestrel therapies in patients with dysfunctional uterine bleeding: The effects on estrogen and progesterone receptor levels and clinical response." Eastern Journal of Medicine 20(4): 199-203.
33. Azizkhani, M., M. V. Dastjerdi, M. T. Arani, R. Pirjani, M. Sepidarkish, F. Ghorat and M. Karimi (2018). "Traditional dry cupping therapy versus medroxyprogesterone acetate in the treatment of idiopathic menorrhagia: a randomized controlled trial." Iranian Red Crescent medical journal 20(2): e60508.
34. Bahamondes, L., V. Marin, S. Ciarmatori, A. L. Silva, J. M. Acuna and M. Y. Makuch (2016). "Knowledge of Latin American Obstetricians and Gynecologists regarding Heavy Menstrual Bleeding." Obstetrics and gynecology international 2016.
35. Bahamondes, M. V., Y. de Lima, V. Teich, L. Bahamondes and I. Monteiro (2012). "Resources and procedures in the treatment of heavy menstrual bleeding with the levonorgestrel-releasing intrauterine system (LNG-IUS) or hysterectomy in Brazil." Contraception 86(3): 244-250.
36. Bahman, M., A. Mirahi, H. Hajimehdipoor and M. Tansaz (2018). "The effect of quince paste on menorrhagia: A clinical study." International Journal of Pharmaceutical Sciences and Research 9(4): 1654-1659.
37. Baron, Y. M., J. Craus, R. C. Agius and M. Brincat (2012). "Synergistic effect on the treatment of menorrhagia by endometrial biopsy followed by contemporaneous insertion of the levonorgestrel intrauterine system." Gynecological endocrinology : the official journal of the International Society of Gynecological Endocrinology 28(9): 694-698.
38. Barr, F., L. Brabin, S. Agbaje, F. Buseri, J. Ikimalo and N. Briggs (2005). "Reducing iron deficiency anaemia due to heavy menstrual blood loss in Nigerian rural adolescents (Reprinted from Public Health Nutrition, vol 1, pg 249-257, 1998)." PUBLIC HEALTH NUTRITION 8(5): 451-460.
39. Barrington, J. W., A. S. Arunkalaivanan and M. Abdel-Fattah (2003). "Comparison between the levonorgestrel intrauterine system (LNG-IUS) and thermal balloon ablation in the treatment of menorrhagia." European journal of obstetrics, gynecology, and reproductive biology 108(1): 72-74.
40. Basaran, H. O., S. Akgul, N. Oksuz-Kanbur, F. Gumruk, M. Cetin and O. Derman (2013). "Dysfunctional uterine bleeding in adolescent girls and evaluation of their response to treatment." Turkish Journal of Pediatrics 55(2): 186-189.
41. Baxi, A., M. Kaushal and A. Ghosh (2008). "The levonorgestrel intrauterine device: An effective and acceptable alternative for the management of menorrhagia." Journal of the Turkish German Gynecology Association 9(4): 202-205.
42. Beaumont, H., C. Augood, K. Duckitt and A. Lethaby (2007). "Danazol for heavy menstrual bleeding." The Cochrane database of systematic reviews(3): CD001017.
43. Beelen, P., M. J. van den Brink, M. C. Herman, P. Geomini, J. H. Dekker, R. G. Duijnhoven, N. Mak, H. S. van Meurs, S. F. Coppus, J. W. van der Steeg, H. P. Eising, D. S. Massop-Helmink, E. R. Klinkert, T. E. Nieboer, A. Timmermans, L. F. van der Voet, S. Veersema, N. A. C. Smeets, J. M. Schutte, M. van Baal, P. M. Bossuyt, B. J. Mol, M. Y. Berger and M. Y. Bongers (2021). "Levonorgestrel-releasing intrauterine system versus endometrial ablation for heavy menstrual bleeding." American journal of obstetrics and gynecology 224(2).
44. Behera, S., B. Gomathi and K. Behera (2023). "Sleep Quality and Depression among women with Abnormal Uterine Bleeding (AUB)." International Journal of Nursing Education 15(2): 60-64.
45. Bender, R. A. (2022). "Medroxyprogesterone Acetate for Abnormal Uterine Bleeding Due to Ovulatory Dysfunction: The Effect of 2 Different-Duration Regimens." Medical Science Monitor 28: e936727.
46. Benetti-Pinto, C. L., T. A. A. de Mira, D. A. Yela, C. R. Teatin-Juliato and L. G. O. Brito (2019). "Pharmacological Treatment for Symptomatic Adenomyosis: A Systematic Review." Revista Brasileira de Ginecologia e Obstetricia 41(9): 564-574.
47. Bergeron, C., P. Y. Laberge, A. Boutin, M. A. Thériault, F. Valcourt, M. Lemyre and S. Maheux-Lacroix (2020). "Endometrial Ablation or Resection Versus Levonorgestrel Intra-uterine System for the Treatment of Women with Heavy Menstrual Bleeding and a Normal Uterine Cavity: A Systematic Review with Meta-analysis." Obstetrical and Gynecological Survey 75(8): 473-474.
48. Bhabani, P., B. P. S. Gaur, Y. Anita and Y. Vikas (2020). "Gynecological problems among adolescent girls in a tertiary care centre of south Andaman district." International journal of adolescent medicine and health 32(4).
49. Bhagyashree, T., M. M. Keerthana, B. H. Raksha, C. Indrani, B. S. Kailashnath and R. Babu (2024). "A Study On Control Of Abnormal Uterine Bleeding By The Use Of Medroxy Progesterone Acetate (MPA) In Reproductive Women At A Tertiary Care Hospital." Research Journal of Pharmaceutical, Biological and Chemical Sciences 15(1): 120-137.
50. Bhattacharya, S., L. J. Middleton, A. Tsourapas, A. J. Lee, R. Champaneria, J. P. Daniels, T. Roberts, N. H. Hilken, P. Barton, R. Gray, K. S. Khan, P. Chien, P. O'Donovan, K. G. Cooper, J. Abbott, J. Barrington, M. Y. Bongers, J. L. Brun, R. Busfield and T. J. Clark (2011). "Hysterectomy, endometrial ablation and Mirena(R) for heavy menstrual bleeding: a systematic review of clinical effectiveness and cost-effectiveness analysis." Health Technology Assessment 15(24): iii-252.
51. Bitzer, J., O. Heikinheimo, A. L. Nelson, J. Calaf-Alsina and I. S. Fraser (2015). "Medical management of heavy menstrual bleeding: a comprehensive review of the literature." Obstetrical & gynecological survey 70(2): 115-130.
52. Bofill Rodriguez, M., S. Dias, V. Jordan, A. Lethaby, S. F. Lensen, M. R. Wise, J. Wilkinson, J. Brown and C. Farquhar (2022). "Interventions for heavy menstrual bleeding; overview of Cochrane reviews and network meta-analysis." The Cochrane database of systematic reviews 5: CD013180.
53. Bofill Rodriguez, M., A. Lethaby and C. Farquhar (2019). "Non-steroidal anti-inflammatory drugs for heavy menstrual bleeding." The Cochrane database of systematic reviews 9: CD000400.
54. Bofill Rodriguez, M., A. Lethaby, C. Farquhar and J. M. Duffy (2020). "Interventions commonly available during pandemics for heavy menstrual bleeding: an overview of Cochrane Reviews." The Cochrane database of systematic reviews 7: CD013651.
55. Bofill Rodriguez, M., A. Lethaby and V. Jordan (2020). "Progestogen-releasing intrauterine systems for heavy menstrual bleeding." The Cochrane database of systematic reviews 6: CD002126.
56. Bofill Rodriguez, M., A. Lethaby, C. Low and I. T. Cameron (2019). "Cyclical progestogens for heavy menstrual bleeding." The Cochrane database of systematic reviews 8: CD001016.
57. Boruah, A. M., P. Jaiswal, M. Chinda and A. Jaiswal (2022). "Real-world Safety and Effectiveness Analysis of Norethisterone in the Management of Abnormal Uterine Bleeding." Journal of SAFOG 14(3): 313-316.
58. Bryant-Smith, A. C., A. Lethaby, C. Farquhar and M. Hickey (2018). "Antifibrinolytics for heavy menstrual bleeding." The Cochrane database of systematic reviews 4: CD000249.
59. Bs, D. and S. K. Nanda (2013). "The role of sevista in the management of dysfunctional uterine bleeding." Journal of clinical and diagnostic research : JCDR 7(1): 132-134.
60. Buhur, A. and O. Unal (2023). "Treatment of abnormal uterine bleeding using levonorgestrel-releasing intrauterine devices: experience from a Turkish tertiary hospital." European review for medical and pharmacological sciences 27(3): 1045-1050.
61. Busfield, R. A., C. M. Farquhar, M. C. Sowter, A. Lethaby, M. Sprecher, Y. Yu, L. C. Sadler, P. Brown and N. Johnson (2006). "A randomised trial comparing the levonorgestrel intrauterine system and thermal balloon ablation for heavy menstrual bleeding." BJOG : an international journal of obstetrics and gynaecology 113(3): 257-263.
62. Buttini, M. J., S. J. Jordan and P. M. Webb (2009). "The effect of the levonorgestrel releasing intrauterine system on endometrial hyperplasia: an Australian study and systematic review." The Australian & New Zealand journal of obstetrics & gynaecology 49(3): 316-322.
63. Cakir, M., I. Mungan, T. Karakas, I. Giriisken and A. Okten (2007). "Menstrual pattern and common menstrual disorders among university students in Turkey." Pediatrics International 49(6): 938-942.
64. Carr, B. R., E. A. Stewart, D. F. Archer, A. Al-Hendy, L. Bradley, N. B. Watts, M. P. Diamond, J. Gao, C. D. Owens, K. Chwalisz, W. R. Duan, A. M. Soliman, M. B. Dufek and J. A. Simon (2018). "Elagolix alone or with add-back therapy in women with heavy menstrual bleeding and uterine leiomyomas: A randomized controlled trial." Obstetrics and gynecology 132(5): 1252-1264.
65. Cetin, N. N., O. Karabacak, U. Korucuoglu and N. Karabacak (2009). "Gonadotropin-releasing hormone analog combined with a low-dose oral contraceptive to treat heavy menstrual bleeding." International Journal of Gynecology and Obstetrics 104(3): 236-239.
66. Chan, S. S. C., K. W. Yiu, P. M. Yuen, D. S. Sahota and T. K. H. Chung (2009). "Menstrual problems and health-seeking behaviour in Hong Kong Chinese girls." Hong Kong medical journal = Xianggang yi xue za zhi 15(1): 18-23.
67. Chandla, A., N. Singh, C. Antony, D. Sharma, A. Tripathi, S. Ota, R. Rana, A. Kumar and N. Srikanth (2023). "Effect of ayurvedic formulations on abnormal uterine bleeding (Asrigdara): a prospective uncontrolled multicenter clinical study." Journal of Herbal Medicine 42.
68. Chattopdhyay, B., A. Nigam, S. Goswami and P. S. Chakravarty (2011). "Clinical outcome of levonorgestrel intra-uterine system in idiopathic menorrhagia." European review for medical and pharmacological sciences 15(7): 764-768.
69. Che, X., J. Wang, W. Sun, J. He, Q. Wang, D. Zhu, W. Zhu, J. Zhang, J. Dong, J. Xu, F. Zheng, J. Zhou, W. Zhao, Q. Lin, L. Ye, X. Zhao, Z. Xu, Y. Chen, J. Wang and W. Wu (2023). "Effect of Mifepristone vs Placebo for Treatment of Adenomyosis With Pain Symptoms: A Randomized Clinical Trial." JAMA Network Open 6(6): e2317860-e2317860.
70. Chegini, M. G., K. Hajizadeh, A. Farshbaf-Khalili, F. Lalooha and M. Shahnazi (2023). "Comparative efficacy of combined oral contraceptive capsules and vitamin D-combined oral contraceptive capsules on ovulatory dysfunction: a randomized clinical trial." Journal of Research Development in Nursing and Midwifery 20(1): 66-72.
71. Chen, C.-M., Y.-N. Tsai, C.-H. Chen, Y.-H. Tseng, S. Jui-Shan Lin and Y.-C. Su (2022). "Levonorgestrel intrauterine devices improve body constitution deviations in the perspective of traditional Chinese medicine and quality of life in patients with chronic pelvic pain and heavy menstrual bleeding." Taiwanese journal of obstetrics & gynecology 61(6): 989-994.
72. Chen, S., J. Liu, S. Peng and Y. Zheng (2022). "LNG-IUS vs. medical treatments for women with heavy menstrual bleeding: A systematic review and meta-analysis." Frontiers in medicine 9: 948709.
73. Chhetri, D. D. and M. S. Singh (2020). "Menstrual characteristics among the nepali adolescent girls." Indian Journal of Public Health Research and Development 11(7): 252-258.
74. Cho, S., A. Nam, H. Kim, D. Chay, K. Park, D. J. Cho, Y. Park and B. Lee (2008). "Clinical effects of the levonorgestrel-releasing intrauterine device in patients with adenomyosis." American journal of obstetrics and gynecology 198(4).
75. Chris-Andrew, W. J., C. J. C. J. Richard, R. Leedah, ntilde and N. ola (2024). "The use of Levonorgestrel-releasing Intrauterine System (LNG-IUS) in the treatment of symptomatic leiomyoma uteri: A systematic review." Philippine Journal of Reproductive Endocrinology and Infertility: 20-27.
76. Cihangir, U., A. Ebru, E. Murat and Y. Levent (2013). "Mechanism of action of the levonorgestrel-releasing intrauterine system in the treatment of heavy menstrual bleeding." International journal of gynaecology and obstetrics: the official organ of the International Federation of Gynaecology and Obstetrics 123(2): 146-149.
77. Cim, N., S. Soysal, S. Sayan, B. Yildizhan, E. Karaman, O. Cetin, H. E. Tolunay and R. Yildizhan (2018). "Two Years Follow-Up of Patients with Abnormal Uterine Bleeding after Insertion of the Levonorgestrel-Releasing Intrauterine System." Gynecologic and obstetric investigation 83(6): 569-575.
78. Cooper, N. A. M., R. Papadantonaki, S. Yorke and K. S. Khan (2022). "Variation of outcome reporting in studies of interventions for heavy menstrual bleeding: a systematic review." FACTS VIEWS AND VISION IN OBGYN 14(3): 205-218.
79. Costanzi, F., M. P. De Marco, C. Colombrino, M. Ciancia, F. Torcia, I. Ruscito, F. Bellati, A. Frega, G. Cozza and D. Caserta (2021). "The treatment with Levonorgestrel Releasing Intrauterine System (LNG-IUS) in patients affected by menometrorrhagia, dysmenorrhea and adenomimyois: clinical and ultrasonographic reports." European review for medical and pharmacological sciences 25(9): 3432-3439.
80. Cozza, G., A. Pinto, V. Giovanale, P. Bianchi, A. Guarino, R. Marziani, A. Frega and D. Caserta (2017). "Comparative effectiveness and impact on health-related quality of life of hysterectomy vs. levonorgestrel intra-uterine system for abnormal uterine bleeding." European review for medical and pharmacological sciences 21(9): 2255-2260.
81. Creinin, M. D., K. T. Barnhart, L. M. Gawron, D. Eisenberg, R. G. Mabey, Jr. and J. T. Jensen (2023). "Heavy Menstrual Bleeding Treatment With a Levonorgestrel 52-mg Intrauterine Device." Obstetrics and gynecology 141(5): 971-978.
82. Dahiya, K., S. Mahipal, A. Dahiya, I. Nandal and P. Narang (2019). "Comparative study on levonorgestrel intrauterine system and oral progestogen in women with heavy menstrual bleeding in terms of efficacy, user satisfaction and quality of life using MMAS score." 8.
83. Dahiya, P., M. Dalal, A. Yadav, K. Dahiya, S. Jain and V. Silan (2016). "Efficacy of combined hormonal vaginal ring in comparison to combined hormonal pills in heavy menstrual bleeding." European journal of obstetrics, gynecology, and reproductive biology 203: 147-151.
84. Das, P., E. Nafees, S. Roy and P. Mahaseth (2023). "TO COMPARE THE SUCCESS IN TREATMENT OF DUB WITH ORMELOXIFENE VS NORETHISTERONE." International Journal of Academic Medicine and Pharmacy 5(3): 1955-1960.
85. Dash, S., J. Mishra, S. S. Behera and S. Rout (2018). "Therapeutic efficacy of levonorgestrel intrauterine system as an alternative to hysterectomy for management of heavy menstrual bleeding in perimenopausal women." Asian Journal of Pharmaceutical and Clinical Research 11(3): 289-292.
86. Davis, A., A. Godwin, J. Lippman, W. Olson and M. Kafrissen (2000). "Triphasic norgestimate-ethinyl estradiol for treating dysfunctional uterine bleeding." Obstetrics and gynecology 96(6): 913-920.
87. de Souza, S. S., A. F. Camargos, C. P. de Rezende, F. A. Pereira, C. A. Araújo and A. L. Silva Filho (2010). "A randomized prospective trial comparing the levonorgestrel-releasing intrauterine system with thermal balloon ablation for the treatment of heavy menstrual bleeding." Contraception 81(3): 226-231.
88. Depes, D. D. B., M. V. M. d. Mata, A. M. G. Pereira, J. A. Martins, M. P. d. Araujo, R. G. C. Lopes and Z. I. K. d. J.-D. Bella (2023). "Comparative study of the levonorgestrel intrauterine system and laparoscopic hysterectomy for the treatment of heavy menstrual bleeding in enlarged uteri." Einstein (Sao Paulo, Brazil) 21: eAO0033.
89. Desai, R. M. (2012). "Efficacy of levonorgestrel releasing intrauterine system for the treatment of menorrhagia due to benign uterine lesions in perimenopausal women." Journal of mid-life health 3(1): 20-23.
90. Devi, K. P. (2007). "Clinical evaluation of Pushyanuga choorna and Lodhrasava in Rakta Pradara (DUB)." Indian Journal of Traditional Knowledge 6(3): 429-431.
91. Dhamangaonkar, P. C., K. Anuradha and A. Saxena (2015). "Levonorgestrel intrauterine system (Mirena): An emerging tool for conservative treatment of abnormal uterine bleeding." Journal of mid-life health 6(1): 26-30.
92. Dhar, S., K. K. Mondal and P. Bhattacharjee (2023). "Influence of lifestyle factors with the outcome of menstrual disorders among adolescents and young women in West Bengal, India." Scientific reports 13(1): 12476.
93. Ding, C., J. Wang, Y. Cao, Y. Pan, X. Lu, W. Wang, L. Zhuo, Q. Tian and S. Zhan (2019). "Heavy menstrual bleeding among women aged 18-50 years living in Beijing, China: prevalence, risk factors, and impact on daily life." BMC women's health 19(1): 27.
94. Divya, S., T. Thomas, R. Ajmeera, A. Hegde, T. Parikh and S. Shivakumar (2023). "Assessment of the menstrual problems among teenage girls: A tertiary care center study." Journal of Pharmacy and Bioallied Sciences 15(5): S281-S284
95. Donnez, J., H. S. Taylor, E. A. Stewart, L. Bradley, E. Marsh, D. Archer, A. Al-Hendy, F. Petraglia, N. Watts, J. P. Gotteland, E. Bestel, P. Terrill, E. Loumaye, A. Humberstone and E. Garner (2022). "Linzagolix with and without hormonal add-back therapy for the treatment of symptomatic uterine fibroids: two randomised, placebo-controlled, phase 3 trials." The Lancet 400(10356): 896-907.
96. Dwivedi, D., N. Singh and U. Gupta (2024). "Prevalence of Menstrual Disorder in Women and Its Correlation to Body Mass Index and Physical Activity." Journal of Obstetrics and Gynecology of India 74(1): 80-87.
97. Eder, S., J. Baker, J. Gersten, R. G. Mabey and T. L. Adomako (2013). "Efficacy and safety of oral tranexamic acid in women with heavy menstrual bleeding and fibroids." Women's health (London, England) 9(4): 397-403.
98. Edlund, M., M. Blomback and G. Fried (2002). "Desmopressin in the treatment of menorrhagia in women with no common coagulation factor deficiency but with prolonged bleeding time." Blood coagulation & fibrinolysis : an international journal in haemostasis and thrombosis 13(3): 225-231.
99. Eftekhar, T., M. Ghaemi, A. Abedi and M. Shirazi (2019). "Comparison of misoprostol and mefenamic acid on reducing menstrual bleeding in patients suffering from heavy menstrual bleeding." Journal of Family and Reproductive Health 13(3): 141-145.
100. Ekpenyong, C. E., K. J. Davis, U. P. Akpan and N. E. Daniel (2011). "Academic stress and menstrual disorders among female undergraduates in Uyo, South Eastern Nigeria - the need for health education." Nigerian journal of physiological sciences : official publication of the Physiological Society of Nigeria 26(2): 193-198.
101. Elah Sadiqi, M. A. and A. A. Salih (2024). "The effect of psychological stress on the menstrual cycle among medical students." JPMA. The Journal of the Pakistan Medical Association 74(10 (Supple-8)): S164-S167.
102. Endrikat, J., H. Shapiro, E. Lukkari-Lax, M. Kunz, W. Schmidt and M. Fortier (2009). "A Canadian, multicentre study comparing the efficacy of a levonorgestrel-releasing intrauterine system to an oral contraceptive in women with idiopathic menorrhagia." Journal of obstetrics and gynaecology Canada : JOGC = Journal d'obstetrique et gynecologie du Canada : JOGC 31(4): 340-347.
103. Eralil, G. J. (2016). "The Effectiveness of Levonorgestrel-Releasing Intrauterine System in the Treatment of Heavy Menstrual Bleeding." Journal of obstetrics and gynaecology of India 66(Suppl 1): 505-512.
104. Ergun, B., O. Kuru, S. Sen and Y. Kilic (2011). "Comparison between roller-ball endometrial ablation and levonorgestrel intrauterine system (LNG-IUS) in the treatment of abnormal uterine bleeding." Turk Jinekoloji ve Obstetrik Dernegi Dergisi 8(4): 259-263.
105. Ergun, B., O. Kuru, S. Sen, Y. Kilic and E. Bastu (2012). "Roller-ball endometrial ablation versus levonorgestrel releasing intrauterine system in the management of abnormal uterine bleeding." Gineco.ro 8(4): 199-201.
106. Es-Haghee Ashteany, S., M. Vahid Dastjerdi, M. Tabarrai, F. Nejatbakhsh, S. N. Sadati Lamardi, A. Rahmani, M. Azizkhani and Z. Tavoli (2023). "Effectiveness of Persian Golnar on Excessive Menstrual Bleeding in Women with Abnormal Uterine Bleeding, Compared to Tranexamic Acid: A Triple-Blind, Randomized Equivalence Trial." Evidence-based complementary and alternative medicine : eCAM 2023: 5355993.
107. Eshaghian, R., M. Mazaheri, M. Ghanadian, S. Rouholamin, A. Feizi and M. Babaeian (2019). "The effect of frankincense (Boswellia serrata, oleoresin) and ginger (Zingiber officinale, rhizoma) on heavy menstrual bleeding: A randomized, placebo-controlled, clinical trial." Complementary therapies in medicine 42: 42-47.
108. Fakhri, M., Z. H. Gardeshi and S. S. Yousefi (2023). "Effects of herbal products on menorrhagia: A systematic review and meta-analysis." Journal of Nursing and Midwifery Sciences 10(3): e138875.
109. Famuyide, A. O., S. K. Laughlin-Tommaso, S. A. Shazly, K. H. Long, D. M. Breitkopf, A. L. Weaver, M. E. McGree, S. A. El-Nashar, M. A. Lemens and M. R. Hopkins (2017). "Medical therapy versus radiofrequency endometrial ablation in the initial treatment of heavy menstrual bleeding (iTOM Trial): A clinical and economic analysis." PloS one 12(11): e0188176.
110. Farhan, I., M. Akhtar and R. Abid (2020). "Prevalence of menstrual disorders in woman of reproductive age group." Pakistan Journal of Medical and Health Sciences 14(1): 194-196.
111. Fathima, A. and A. Sultana (2012). "Clinical efficacy of a Unani formulation 'Safoof Habis' in menorrhagia: A randomized controlled trial." European Journal of Integrative Medicine 4(3): e315-e322.
112. Fawzy, M. and Y. Mesbah (2015). "Comparison of dienogest versus triptorelin acetate in premenopausal women with adenomyosis: a prospective clinical trial." Archives of gynecology and obstetrics 292(6): 1267-1271.
113. Fayaz, F., N. Khurshid and H. Ismail (2023). "Menstrual Abnormalities Among Adolescent Females Attending a Tertiary Care Hospital in North India." 9.
114. Fazmiya, M. J. A., A. Sultana, M. B. B. Heyat, S. Parveen, K. Rahman, F. Akhtar, A. A. Khan, A. M. Alanazi, Z. Ahmed, I. d. l. T. Diez, J. B. Ballester and T. S. K. Saripalli (2024). "Efficacy of a vaginal suppository formulation prepared with Acacia arabica (Lam.) Willd. gum and Cinnamomum camphora (L.) J. Presl. in heavy menstrual bleeding analyzed using a machine learning technique." Frontiers in pharmacology 15: 1331622.
115. Fraser, I. S., J. Jensen, M. Schaefers, U. Mellinger, S. Parke and M. Serrani (2012). "Normalization of blood loss in women with heavy menstrual bleeding treated with an oral contraceptive containing estradiol valerate/dienogest." Contraception 86(2): 96-101.
116. Fraser, I. S., S. Parke, U. Mellinger, A. MacHlitt, M. Serrani and J. Jensen (2011). "Effective treatment of heavy and/or prolonged menstrual bleeding without organic cause: Pooled analysis of two multinational, randomised, double-blind, placebo-controlled trials of oestradiol valerate and dienogest." European Journal of Contraception and Reproductive Health Care 16(4): 258-269.
117. Fraser, I. S., T. Romer, S. Parke, S. Zeun, U. Mellinger, A. MacHlitt and J. T. Jensen (2011). "Effective treatment of heavy and/or prolonged menstrual bleeding with an oral contraceptive containing estradiol valerate and dienogest: A randomized, double-blind Phase III trial." Human Reproduction 26(10): 2698-2708.
118. Freeman, E. W., A. Lukes, D. Van Drie, R. G. Mabey, J. Gersten and T. L. Adomako (2011). "A dose-response study of a novel, oral tranexamic formulation for heavy menstrual bleeding." American journal of obstetrics and gynecology 205(4): 319.
119. G, P. P., A. Balaji, P. D. A and S. Jeyaraman (2022). "Gynaecological Disorders Among Adolescent Girls Attending a Tertiary Care Hospital in South India: A Cross-Sectional Study." Online Journal of Health & Allied Sciences 21(3): 1-5.
120. Galati, G., G. Ruggiero, A. Grobberio, O. Capri, D. Pietrangeli, N. Recine, M. Vignali and L. Muzii (2024). "The Role of Different Medical Therapies in the Management of Adenomyosis: A Systematic Review and Meta-Analysis." Journal of clinical medicine 13(11): 3302.
121. Ganatra, A., F. Shah and K. Ganatra (2024). "Efficacy of Levonorgestrel Intrauterine System in the Management of Abnormal Uterine Bleeding: A Retrospective Analysis of a 100 Women." Journal of obstetrics and gynaecology of India 74(1): 67-70.
122. Gerema, U., K. Kene, D. Abera, T. Adugna, M. Nigussie, D. Dereje and T. Mulugeta (2022). "Abnormal uterine bleeding and associated factors among reproductive age women in Jimma town, Oromia Region, Southwest Ethiopia." Women's health (London, England) 18: 17455057221077577.
123. Gezer, S., E. Kole and L. Aksoy (2023). "Vaginal micronized progesterone versus the levonorgestrel-releasing intrauterine system for treatment of non-atypical endometrial hyperplasia: A randomized controlled trial." International journal of gynaecology and obstetrics: the official organ of the International Federation of Gynaecology and Obstetrics 161(2): 661-666.
124. Ghazizadeh, S., F. Bakhtiari, H. Rahmanpour, F. Davari-Tanha and F. Ramezanzadeh (2011). "A randomized clinical trial to compare levonorgestrel-releasing intrauterine system (Mirena) vs trans-cervical endometrial resection for treatment of menorrhagia." International journal of women's health 3: 207-211.
125. Ghazizadeh, S., Z. Panahi, Z. Ghanbari, A. T. Menshadi, T. Farahmandian and P. Javadian (2014). "Comparative efficacy of novasure, the levonorgestrel-releasing intrauterine system, and hysteroscopic endometrial resection in the treatment of menorrhagia: A randomized clinical trial." Journal of gynecologic surgery 30(4): 215-218.
126. Godha, Z., Z. Mohsin, S. Hakim and S. Wasim (2016). "Comparative Study of Ormeloxifene and Medroxyprogesterone Acetate in Abnormal Uterine Bleeding." Journal of Obstetrics and Gynecology of India 66(Supplement 1): 395-399.
127. Goni, A. Z., R. L. Lacruz, J. J. P. Paricio and F. J. Hernandez Rivas (2009). "The levonorgestrel intrauterine system as an alternative to hysterectomy for the treatment of idiopathic menorrhagia." Gynecological endocrinology : the official journal of the International Society of Gynecological Endocrinology 25(9): 581-586.
128. Gopimohan, R., A. Chandran, J. Jacob, S. Bhaskar, R. Aravindhakshan and A. S. Aprem (2015). "A clinical study assessing the efficacy of a new variant of the levonorgestrel intrauterine system for abnormal uterine bleeding." International journal of gynaecology and obstetrics: the official organ of the International Federation of Gynaecology and Obstetrics 129(2): 114-117.
129. Gorgen, H., M. Api, A. Akca and A. Cetin (2009). "Use of the Levonorgestrel-IUS in the treatment of menorrhagia: assessment of quality of life in Turkish users." Archives of gynecology and obstetrics 279(6): 835-840.
130. Goshtasebi, A., Z. Mazari, S. Behboudi Gandevani and M. Naseri (2015). "Anti-hemorrhagic activity of Punica granatum L. flower (Persian Golnar) against heavy menstrual bleeding of endometrial origin: a double-blind, randomized controlled trial." Medical journal of the Islamic Republic of Iran 29: 199.
131. Goshtasebi, A., S. Moukhah and S. B. Gandevani (2013). "Treatment of heavy menstrual bleeding of endometrial origin: randomized controlled trial of medroxyprogesterone acetate and tranexamic acid." Archives of gynecology and obstetrics 288(5): 1055-1060.
132. Grover, S., A. Chhabra and S. Bindu (2013). "A Study of Ormeloxifene in Case of Dysfunctional Uterine Bleeding."
133. Guan, L., L. Xue, J. Chu, J. Xue, S. Zhang and L. Zhu (2021). "Effect of Tiaojingzhixue Fang on the expression of sex hormone and endometrial tissue mRNA in perimenopausal patients with abnormal uterine bleeding." Cellular and Molecular Biology 67(5): 317-323.
134. Gultekin, M., K. Diribas, E. Buru and M. A. Gokceoglu (2009). "Role of a non-hormonal oral anti-fibrinolytic hemostatic agent (tranexamic acid) for management of patients with dysfunctional uterine bleeding." Clinical and experimental obstetrics & gynecology 36(3): 163-165.
135. Gupta, A., N. Gangane, M. Dhanvij, B. Moharana, M. Davile and S. Mundle (2024). "The Effect of the Levonorgestrel-Releasing Intrauterine System on Myometrial Blood Flow in Patients With Heavy Menstrual Bleeding." Cureus 16(8): e66712.
136. Gupta, B., S. Mittal, R. Misra, D. Deka and V. Dadhwal (2006). "Levonorgestrel-releasing intrauterine system vs. transcervical endometrial resection for dysfunctional uterine bleeding." International journal of gynaecology and obstetrics: the official organ of the International Federation of Gynaecology and Obstetrics 95(3): 261-266.
137. Gupta, J., J. Kai, L. Middleton, H. Pattison, R. Gray, J. Daniels, G. J. D. J. M. L. G. R. P. H. G. J. K. J. D. J. M. L. G. R. P. H. G. L. Kai J and G. Eclipse Trial Collaborative (2013). "Levonorgestrel intrauterine system versus medical therapy for menorrhagia." The New England journal of medicine 368(2): 128-137.
138. Haberal, A., F. Kayikcioglu, M. Gunes, O. Ozdegirmanci and M. Kaplan (2005). "Efficacy of Levonorgestrel Releasing Intrauterine System in Menorrhagia: A One-Year Follow-up Study." Journal of the Turkish-German Gynecological Association 6(2): 130-133.
139. Haimovich, S., M. A. Checa, G. Mancebo, P. Fuste and R. Carreras (2008). "Treatment of endometrial hyperplasia without atypia in peri- and postmenopausal women with a levonorgestrel intrauterine device." Menopause 15(5): 1002-1004.
140. Han, J., X. Wang, W. Lv, R. Tian and L. Guan (2021). "Comparison between direct use and PLGA nanocapsules containing drug of traditional Chinese medicine, Tiaojing Zhixue, in treatment of dysfunctional uterine bleeding." Cellular and Molecular Biology 67(3): 138-142.
141. Hangekar, P. M., G. Mhaske and G. Shekhawat (2019). "Mirena: a novel alternative to hysterectomy." 8.
142. Harada, T., I. Ota, J. Kitawaki, M. Momoeda, N. Maeda, S. Akira, M. Umeyama, T. Sunaya and K. Hirano (2022). "Real-world outcomes of the levonorgestrel-releasing intrauterine system for heavy menstrual bleeding or dysmenorrhea in Japanese patients: A prospective observational study (J-MIRAI)." Contraception 116: 22-28.
143. Harlow, S. D. and O. M. R. Campbell (2004). "Epidemiology of menstrual disorders in developing countries: a systematic review." BJOG: An International Journal of Obstetrics and Gynaecology 111(1): 6-16.
144. Health Quality Ontario (2016). "Levonorgestrel-Releasing Intrauterine System (52 mg) for Idiopathic Heavy Menstrual Bleeding: A Health Technology Assessment." Ontario health technology assessment series 16(18): 1-119.
145. Henshaw, R., C. Coyle, S. Low and C. Barry (2002). "A retrospective cohort study comparing microwave endometrial ablation with levonorgestrel-releasing intrauterine device in the management of heavy menstrual bleeding." The Australian & New Zealand journal of obstetrics & gynaecology 42(2): 205-209.
146. Hickey, M., J. Higham and I. S. Fraser (2000). "Progestogens versus oestrogens and progestogens for irregular uterine bleeding associated with anovulation." The Cochrane database of systematic reviews(2): CD001895.
147. Hoaglin, D. C., A. Filonenko, M. E. Glickman, R. Wasiak and R. Gidwani (2013). "Use of mixed-treatment-comparison methods in estimating efficacy of treatments for heavy menstrual bleeding." European journal of medical research 18: 17.
148. Holler, M., H. Steindl, D. Abramov-Sommariva, J. Kleemann, A. Loleit, C. Abels and P. Stute (2024). "Use of Vitex agnus-castus in patients with menstrual cycle disorders: a single-center retrospective longitudinal cohort study." Archives of gynecology and obstetrics 309(5): 2089-2098.
149. Huijs, D. P. C., A. J. M. Derickx, P. Beelen, J. C. Leemans, S. M. J. van Kuijk, M. Y. Bongers and P. M. A. J. Geomini (2024). "A 52-mg levonorgestrel-releasing intrauterine system vs bipolar radiofrequency nonresectoscopic endometrial ablation in women with heavy menstrual bleeding: long-term follow-up of a multicenter randomized controlled trial." American journal of obstetrics and gynecology 230(5): 542.e541-542.e510.
150. Ibraheim, M. and A. Ikomi (2005). "An evaluation of troublesome inter-menstrual bleeding in menorrhagic users of the LNG-IUS." Journal of obstetrics and gynaecology : the journal of the Institute of Obstetrics and Gynaecology 25(4): 384-385.
151. Ibrahim Mohamed, E. M. and H. Hassan Mansour (2009). Oral versus rectal misoprostol in the treatment of menorrhagia. 45: 721-725.
152. Ibrahim, P. M. and E. L. Samwel (2023). "Prevalence of Heavy Menstrual Bleeding and Its Associated Factors Among Women Attending Kilimanjaro Christian Medical Centre In Northern Eastern, Tanzania: A Cross-Sectional Study." The East African health research journal 7(1): 1-6.
153. Igarashi, M., Y. Abe, M. Fukuda, A. Ando, M. Miyasaka and M. Yoshida (2000). "Novel conservative medical therapy for uterine adenomyosis with a danazol-loaded intrauterine device." Fertility and sterility 74(2): 412-413.
154. Ikomi, A., E. Mansell, C. Spence-Jones and A. Singer (2000). "Treatment of menorrhagia with the levonorgestrel intrauterine system: can we learn from our failures?" Journal of obstetrics and gynaecology : the journal of the Institute of Obstetrics and Gynaecology 20(6): 630-631.
155. Ilyin, A. B., A. A. Khasanov, L. V. Suturina, N. I. Borisova, Z. S. Reshetov, J.-M. Foidart, J. A. Deri and V. Toth (2021). "Comparison of two levonorgestrel-releasing intrauterine systems for the treatment of heavy menstrual bleeding: a randomised, controlled, phase 3 trial." The European journal of contraception & reproductive health care : the official journal of the European Society of Contraception 26(6): 491-498.
156. Indu, V., J. Gaurika, S. Dinesh and R. K. Soni (2020). "Menstrual problems in undergraduate medical students: A cross-sectional study in a medical college of north india." Journal of SAFOG 12(2): 85-90
157. Irahara, M., Y. Maejima, N. Shinbo, Y. Yamauchi and H. Mizunuma (2020). "Ulipristal acetate for Japanese women with symptomatic uterine fibroids: A double-blind, randomized, phase II dose-finding study." Reproductive medicine and biology 19(1): 65-74.
158. Istre, O. and B. Trolle (2001). "Treatment of menorrhagia with the levonorgestrel intrauterine system versus endometrial resection." Fertility and sterility 76(2): 304-309.
159. Iyer, V., C. Farquhar and R. Jepson (2000). "Oral contraceptive pills for heavy menstrual bleeding." The Cochrane database of systematic reviews(2): CD000154.
160. Jafari, A., Z. Latifiyan, R. Torkashvand and M. Saatchi (2019). "The effect of vitamin B1 on heavy menstrual bleeding." Progress in Nutrition 21(4): 843-848.
161. Jahanfar, S., J. Mortazavi, A. Lapidow, C. Cu, J. Al Abosy, H. Ciana, K. Morris, M. Steinfeldt, O. Maurer, J. Bohang, R. Anjali Oberoi and M. Ali (2024). "Assessing the impact of hormonal contraceptive use on menstrual health among women of reproductive age - a systematic review." The European journal of contraception & reproductive health care : the official journal of the European Society of Contraception 29(5): 193-223.
162. Jain, S., N. B. Vaid, Y. Narang, A. Suneja and K. Guleria (2016). "A Randomised Controlled Trial Comparing the Efficacy and Side-Effects of Intravaginal Ring (Nuvaring( R)) With Combined Oral Hormonal Preparation in Dysfunctional Uterine Bleeding." Journal of clinical and diagnostic research : JCDR 10(3): QC21-24.
163. Jamila Mehnaz, N., S. Muhammad Ilyas and A. Wajiha (2003). <The> role of dydrogesterone in the medical management of 100 cases of dysfunctional uterine bleeding [DUB] above 35 years of age. 17: 189-193.
164. Janssen, C. A., P. C. Scholten and A. P. Heintz (2000). "The effect of low-dose 3-keto-desogestrel added to a copper-releasing intrauterine contraceptive device on menstrual blood loss: a double-blind, dose-finding, placebo-controlled study." American journal of obstetrics and gynecology 182(3): 575-581.
165. Javan, R., M. Yousefi, S.-M. Nazari, P. Amiri, A. Mosavi-Jarrahi, P. Modiramani and H. Naghedi-Baghdar (2016). "Herbal Medicines in Idiopathic Heavy Menstrual Bleeding: A Systematic Review." Phytotherapy research : PTR 30(10): 1584-1591.
166. Jayanthi, S., N. Kumari and R. Pushpa (2019). "A cross-sectional study on obesity and menstrual abnormalities among women of reproductive age in urban field practice area of Kempegowda Institute of Medical Sciences, Bangalore." 6.
167. Jensen, J. T., S. Parke, U. Mellinger, A. Machlitt and I. S. Fraser (2011). "Effective treatment of heavy menstrual bleeding with estradiol valerate and dienogest: a randomized controlled trial." Obstetrics and gynecology 117(4): 777-787.
168. Jha, P. and Seema (2023). "Study of Menstrual Problems of Adolescent Girls Attending a Tertiary Care Hospital." International Journal of Pharmaceutical and Clinical Research 15(9): 1189-1192.
169. Johar, M. J., N. Akhtar, S. Akram, F. Sabir, F. Burki and K. Rehman (2023). "Evaluation of Applications of Intrauterine Progesterone Versus Oral Progesterone in the Treatment of Dysfunctional Uterine Bleeding." Pakistan Journal of Medical and Health Sciences 17(3): 328-330.
170. Juneja, S. K., P. Tandon and G. Kaur (2020). "Comparison of safety and efficacy of Ormeloxifene and Norethisterone acetate in the treatment of heavy menstrual bleeding." 9
171. Ju-Qi, M., G. Chun-Fen and A. Hasim (2018). "Clinical efficacy of levonorgestrel releasing intrauterine system for the treatment of adenomyosis in perimenopausal women." Clinical and Experimental Obstetrics and Gynecology 45(3): 387-390.
172. Jyotsna, S., D. Mekhala, M. B. Bellad, M. S. Ganachari and H. A. Dhumale (2011). "Ormeloxifene versus medroxyprogesterone acetate (MPA) in the treatment of dysfunctional uterine bleeding: a double-blind randomized controlled trial." Journal of SAFOG (South Asian Federation of Obstetrics and Gynaecology) 3(1): 21-24.
173. Kacholi, D. S., O. J. Kalokora, H. M. Amir and N. G. Mogha (2023). "Ethnogynaecological medicinal plants used by Tanzanian communities against female infertility and menstrual disorders - a comprehensive review." Ethnobotany Research and Applications 26.
174. Kahal, F., S. Alshayeb, A. Torbey, O. AlHelwani, S. Kadri, A. Helwani, S. Al-Habal, M. Moufti, M. Johari, A. Aldarra, G. Alswaedan, S. Albaghajati, H. Sarraj, S. Ataya, M. Mansour and K. Sakka (2024). "The prevalence of menstrual disorders and their association with psychological stress in Syrian students enrolled at health-related schools: A cross-sectional study." International Journal of Gynecology and Obstetrics 164(3): 1086-1093.
175. Kai, J., B. Dutton, Y. Vinogradova, N. Hilken, J. Gupta and J. Daniels (2022). "Medical treatment for heavy menstrual bleeding in primary care: 10-year data from the ECLIPSE trial." The British journal of general practice : the journal of the Royal College of General Practitioners 72(725): e857-e864.
176. Kai, J., L. Middleton, J. Daniels, H. Pattison, K. Tryposkiadis, J. Gupta and g. Eclipse trial collaborative (2016). "Usual medical treatments or levonorgestrel-IUS for women with heavy menstrual bleeding: long-term randomised pragmatic trial in primary care." The British journal of general practice : the journal of the Royal College of General Practitioners 66(653): e861-e870.
177. Karamustafaoğlu Balcı, B., M. Hocaoğlu, G. Göynümer and A. Göçmen (2016). "Levonorgestrel containing intrauterine device (Mirena®) in the treatment of dyfunctional uterine bleeding; patients’ view and our experience." Medeniyet Medical Journal 31(4): 278-281.
178. Karimi-Zarchi, M., M. Z. Abadinezhad, B. Bonyadpour, S. Kabyrpour-Ashkezar and A. M. Abhaji (2016). "Evaluate the Effectiveness of Conservative Treatment for Menorrhagia in Women who were Admitted to the Shahid Sadoughi Hospital of Yazd-Iran in 2014-2015." International journal of biomedical science : IJBS 12(3): 105-109.
179. Karimi-Zarchi, M., R. Dehghani-Firoozabadi, A. Tabatabaie, Z. Dehghani-Firoozabadi, S. Teimoori, Z. Chiti, A. Miratashi-Yazdi and A. Dehghani (2013). "A comparison of the effect of levonorgestrel IUD with oral medroxyprogesterone acetate on abnormal uterine bleeding with simple endometrial hyperplasia and fertility preservation." Clinical and experimental obstetrics & gynecology 40(3): 421-424.
180. Karout, N., S. M. Hawai and S. Altuwaijri (2012). "Prevalence and pattern of menstrual disorders among Lebanese nursing students." Eastern Mediterranean health journal = La revue de sante de la Mediterranee orientale = al-Majallah al-sihhiyah li-sharq al-mutawassit 18(4): 346-352.
181. Karthikkeyan, K., N. N, T. K. S. P, C. V and D. B (2020). "Prevalence of menstrual morbidity in adolescents girls: a cross sectional study." 8.
182. Katiyar, K., H. Chopra, S. K. Garg, S. K. Bajpai, T. Bano, S. Jain and A. Kumar (2013). "KAP study of menstrual problems in adolescent females in an urban area of Meerut." Indian Journal of Community Health 25(3): 217-220.
183. Kaunitz, A. M., F. Bissonnette, I. Monteiro, E. Lukkari-Lax, C. Muysers and J. T. Jensen (2010). "Levonorgestrel-releasing intrauterine system or medroxyprogesterone for heavy menstrual bleeding: A randomized controlled trial (Obstetrics and Gynecology (2010) 116 (625-632))." Obstetrics and gynecology 116(4): 999.
184. Kaunitz, A. M., S. Meredith, P. Inki, A. Kubba and L. Sanchez-Ramos (2009). "Levonorgestrel-releasing intrauterine system and endometrial ablation in heavy menstrual bleeding: a systematic review and meta-analysis." Obstetrics and gynecology 113(5): 1104-1116.
185. Khalighi, M., A. P. Wheeler, O. A. Adeyemi-Fowode, P. A. Kouides, R. A. Durazo-Arvizu, K. Haley, C. M. Dersch, A. C. Weyand, M. K. Baldwin and C. Borzutzky (2022). "Does a Bleeding Disorder Lessen the Efficacy of the 52-mg Levonorgestrel-Releasing Intrauterine System for Heavy Menstrual Bleeding in Adolescents? A Retrospective Multicenter Study." The Journal of adolescent health : official publication of the Society for Adolescent Medicine 71(2): 204-209.
186. Khan, E., W. A. Qazi, S. Jahan, A. Naeem and N. A. Bokhari (2019). "Association of obesity and anemia with menstrual irregularities in young girls between the ages of 16 to 25 years." Rawal Medical Journal 44(2): 237-239.
187. Khodabakhsh, M., M. Mahmoudinia, M. M. Bazaz, S. S. Hamedi, S. S. Hoseini, Z. Feyzabadi, S. Shokri and S. Ayati (2020). "The effect of plantain syrup on heavy menstrual bleeding: A randomized triple blind clinical trial." Phytotherapy Research 34(1): 118-125.
188. Kiseli, M., F. Kayikcioglu, O. Evliyaoglu and A. Haberal (2016). "Comparison of Therapeutic Efficacies of Norethisterone, Tranexamic Acid and Levonorgestrel-Releasing Intrauterine System for the Treatment of Heavy Menstrual Bleeding: A Randomized Controlled Study." Gynecologic and obstetric investigation 81(5): 447-453.
189. Kitawaki, J., S. Akira, T. Harada, N. Maeda, M. Momoeda, I. Ota, T. Murakami, T. Sunaya and K. Hirano (2022). "Bleeding patterns of women with heavy menstrual bleeding or dysmenorrhoea using the levonorgestrel-releasing intrauterine system: results from a real-world observational study in Japan (J-MIRAI)." The European journal of contraception & reproductive health care : the official journal of the European Society of Contraception 27(4): 300-307.
190. Koh, S. C. L. and K. Singh (2007). "The effect of levonorgestrel-releasing intrauterine system use on menstrual blood loss and the hemostatic, fibrinolytic/inhibitor systems in women with menorrhagia." Journal of thrombosis and haemostasis : JTH 5(1): 133-138.
191. Kouides, P. A., V. R. Byams, C. S. Philipp, S. F. Stein, J. A. Heit, A. S. Lukes, N. I. Skerrette, N. F. Dowling, B. L. Evatt, C. H. Miller, S. Owens and R. Kulkarni (2009). "Multisite management study of menorrhagia with abnormal laboratory haemostasis: a prospective crossover study of intranasal desmopressin and oral tranexamic acid." British journal of haematology 145(2): 212-220.
192. Kriplani, A., V. Kulshrestha and N. Agarwal (2009). "Efficacy and safety of ormeloxifene in management of menorrhagia: A pilot study." Journal of Obstetrics and Gynaecology Research 35(4): 746-752.
193. Kriplani, A., V. Kulshrestha, N. Agarwal and S. Diwakar (2006). "Role of tranexamic acid in management of dysfunctional uterine bleeding in comparison with medroxyprogesterone acetate." Journal of obstetrics and gynaecology : the journal of the Institute of Obstetrics and Gynaecology 26(7): 673-678.
194. Kriplani, A., B. M. Singh, S. Lal and N. Agarwal (2007). "Efficacy, acceptability and side effects of the levonorgestrel intrauterine system for menorrhagia." International journal of gynaecology and obstetrics: the official organ of the International Federation of Gynaecology and Obstetrics 97(3): 190-194.
195. Kriplani, A., A. Srivastava, V. Kulshrestha, G. Kachhawa, N. Agarwal, N. Bhatla and S. Hari (2016). "Efficacy of ormeloxifene versus oral contraceptive in the management of abnormal uterine bleeding due to uterine leiomyoma." The journal of obstetrics and gynaecology research 42(12): 1744-1752.
196. Kucuk, T. and K. Ertan (2008). "Continuous oral or intramuscular medroxyprogesterone acetate versus the levonorgestrel releasing intrauterine system in the treatment of perimenopausal menorrhagia: a randomized, prospective, controlled clinical trial in female smokers." Clinical and experimental obstetrics & gynecology 35(1): 57-60.
197. Kulshrestha, V., A. Kriplani, N. Agarwal, N. Sareen, P. Garg, S. Hari and J. Thulkar (2013). "Low dose mifepristone in medical management of uterine leiomyoma - an experience from a tertiary care hospital from north India." The Indian journal of medical research 137(6): 1154-1162.
198. Kumar, A. and J. Prakash (2023). "A Descriptive Cross-Sectional Analysis of the Menstrual Cycle and Associated Issues in Adolescent Girls." International Journal of Current Pharmaceutical Review and Research 15(2): 870-874.
199. Kumar, S., U. Tekur, B. Singh and D. Kumar (2018). "Mefenamic acid and diclofenac in the treatment of menorrhagia and dysmenorrhea in dysfunctional uterine bleeding: a randomized comparative study." 7.
200. Kumari, T., S. Bhushan and A. Jha (2023). "A Clinic-Demographic Profile and Assessment of Menstrual Problems of Adolescent Girls Attending Tertiary Care Facility." International Journal of Current Pharmaceutical Review and Research 15(9): 61-65.
201. Kyeong, H. K., J. Choi, Y. J. Na and H. G. Kim (2023). "Effects of long-term intermittent pharmacological therapy with ulipristal acetate on reducing the volume of uterine fibroids and relieving symptoms." Minerva obstetrics and gynecology 75(3): 236-242.
202. Laksham, K. B., S. Ramya and S. S. Kar (2019). "Menstrual disorders and quality of life of women in an urban area of Puducherry: a community-based cross-sectional study." Journal of family medicine and primary care 8(1): 137-140.
203. Learman, L. A., R. L. Summitt Jr, R. E. Varner, H. E. Richter, F. Lin, C. C. Ireland, M. Kuppermann, E. Vittinghoff, J. Showstack, A. E. Washington and S. B. Hulley (2004). "Hysterectomy versus expanded medical treatment for abnormal uterine bleeding: Clinical outcomes in the medicine or surgery trial." Obstetrics and gynecology 103(5 I): 824-833.
204. Lee, B. S., X. Ling, S. Asif, P. Kraemer, J. U. Hanisch and P. Inki (2013). "Levonorgestrel-releasing intrauterine system versus conventional medical therapy for heavy menstrual bleeding in the Asia-Pacific region." International journal of gynaecology and obstetrics: the official organ of the International Federation of Gynaecology and Obstetrics 121(1): 24-30.
205. Lee, B. S., X. Ling, S. Asif, P. Kraemer, J. U. Hanisch, P. Inki and J. E. Lee (2015). "Therapy of heavy menstrual bleeding in Korea: Subanalysis and results from a multinational clinical trial in the Asian region investigating the levonorgestrel-releasing intrauterine system versus conventional therapy." Obstetrics & gynecology science 58(2): 162-170.
206. Lee, L. K., P. C. Y. Chen, K. K. Lee and J. Kaur (2006). "Menstruation among adolescent girls in Malaysia: a cros ssectional school survey." Singapore medical journal 47(10): 869-874.
207. Lele, M., X. Sisi, B. Wenpei, Y. Chen, Z. Yingfang, C. Xing, S. Yu, L. Mao, S. Xi, W. Bai, C. Yao, Y. Zhou, X. Chen and Y. Sun (2021). "Menstrual patterns and disorders among Chinese women of reproductive age: A cross-sectional study based on mobile application data." Medicine 100(16): 1-6.
208. Lete, I., C. Obispo, F. Izaguirre, T. Orte, B. Rivero, M. J. Cornellana, I. Bermejo and O. Behalf Spanish Soc Gynaecology (2008). "The levonorgestrel intrauterine system (Mirena (R)) for treatment of idiopathic menorrhagia. Assessment of quality of life and satisfaction." European Journal of Contraception and Reproductive Health Care 13(3): 231-237.
209. Lethaby, A., C. Augood and K. Duckitt (2000). "Nonsteroidal anti-inflammatory drugs for heavy menstrual bleeding." The Cochrane database of systematic reviews(2): CD000400.
210. Lethaby, A., I. Cooke, M. C. Rees, A. Lethaby, M. Hussain, J. R. Rishworth and M. C. Rees (2015). "Progesterone or progestogen-releasing intrauterine systems for heavy menstrual bleeding." Cochrane Database of Systematic Reviews(4): N.PAG-N.PAG.
211. Lethaby, A., K. Duckitt and C. Farquhar (2013). "Non-steroidal anti-inflammatory drugs for heavy menstrual bleeding." The Cochrane database of systematic reviews(1): CD000400.
212. Lethaby, A., G. Irvine and I. Cameron (2008). "Cyclical progestogens for heavy menstrual bleeding." The Cochrane database of systematic reviews(1): CD001016.
213. Lethaby, A., M. R. Wise, M. A. Weterings, M. Bofill Rodriguez and J. Brown (2019). "Combined hormonal contraceptives for heavy menstrual bleeding." The Cochrane database of systematic reviews 2: CD000154.
214. Li, L., J. Leng, S. Jia and J. Lang (2019). "Treatment of symptomatic adenomyosis with the levonorgestrel-releasing intrauterine system." International journal of gynaecology and obstetrics: the official organ of the International Federation of Gynaecology and Obstetrics 146(3): 357-363.
215. Liu, Z., Q. V. Doan, P. Blumenthal and R. W. Dubois (2007). "A systematic review evaluating health-related quality of life, work impairment, and health-care costs and utilization in abnormal uterine bleeding." Value in Health 10(3): 183-194.
216. Lukes, A. S., K. A. Moore, K. N. Muse, J. K. Gersten, B. R. Hecht, M. Edlund, H. E. Richter, S. E. Eder, G. R. Attia, D. L. Patrick, A. Rubin and G. A. Shangold (2010). "Tranexamic acid treatment for heavy menstrual bleeding: a randomized controlled trial." Obstetrics and gynecology 116(4): 865-875.
217. Lunardi Rocha, A. L., M. Cristina Franca Ferreira, R. Mara Lamaita, E. Batista Candido, M. Mendonca Carneiro and A. Lopes da Silva-Filho (2018). "Heavy menstrual bleeding: a global survey of health care practitioners' perceptions." The European journal of contraception & reproductive health care : the official journal of the European Society of Contraception 23(4): 288-294.
218. Magalhaes, J., J. M. Aldrighi and G. R. de Lima (2007). "Uterine volume and menstrual patterns in users of the levonorgestrel-releasing intrauterine system with idiopathic menorrhagia or menorrhagia due to leiomyomas." Contraception 75(3): 193-198.
219. Majeed, J., P. Sharma, P. Ajmera and K. Dalal (2022). "Menstrual hygiene practices and associated factors among Indian adolescent girls: a meta-analysis." Reproductive health 19(1): 148.
220. Malik, F., S. Sara and R. Kasi (2020). "Comparative trial of levonorgestrel intrauterine system and norethisterone for treatment of idiopathic menorrhagia." Pakistan Journal of Medical and Health Sciences 14(4): 1184-1186.
221. Malik, R., K. Kumari, H. Goel and A. Hada (2024). "Effectiveness of Low Dose Mifepristone in Medical Management of Fibroids." Journal of South Asian Federation of Obstetrics and Gynaecology 16(2): 98-102.
222. Malik, S., S. Musharaf, F. Malik and M. Abass (2019). "Efficacy and safety of ormeloxifene in the management of dysfunctional uterine bleeding." 8.
223. Manasi, P. S., B. K. Kavitha and M. Parvathy (2021). "A CLINICAL STUDY FOR THE EVALUATION OF THE EFFECT OF BALA MOOLA CHURNA IN ASRIGDARA." International Journal of Research in Ayurveda and Pharmacy 13(1): 17-20.
224. Mani, A., K. Sharma, A. Kumar and R. K. Talukdar (2019). "Selective estrogen receptor modulator: efficacy in abnormal uterine bleeding in perimenopausal women." 8.
225. Mansukhani, N., J. Unni, M. Dua, R. Darbari, S. Malik, S. Verma and S. Bathla (2013). "Are women satisfied when using levonorgestrel-releasing intrauterine system for treatment of abnormal uterine bleeding?" Journal of mid-life health 4(1): 31-35.
226. Mariappen U, Chew KT, Zainuddin AA, Mahdy ZA, Abdul Ghani NA, Grover S. Quality of life of adolescents with menstrual problems in Klang Valley, Malaysia: a school population-based cross-sectional study. BMJ Open. 2022 Jan 4;12(1):e051896. doi: 10.1136/bmjopen-2021-051896. PMID: 34983763; PMCID: PMC8728463.
227. Marisiddaiah, M. (2023). "Comparative study between ormeloxifene and norethisterone for management of dysfunctional uterine bleeding (HMB)." Journal of Cardiovascular Disease Research 14(8): 1907-1910.
228. Marjoribanks, J., A. Lethaby and C. Farquhar (2016). "Surgery versus medical therapy for heavy menstrual bleeding." The Cochrane database of systematic reviews(1): CD003855.
229. Matteson, K. A., H. Abed, T. L. Wheeler, 2nd, V. W. Sung, D. D. Rahn, J. I. Schaffer, E. M. Balk, U. K. L. J. C. M. M. Y. M. M. L. L. M. M. M. I. D. H. O. W. B. B. M. Rogers Rg and G. Society of Gynecologic Surgeons Systematic Review (2012). "A systematic review comparing hysterectomy with less-invasive treatments for abnormal uterine bleeding." Journal of minimally invasive gynecology 19(1): 13-28.
230. Matteson, K. A., D. D. Rahn, T. L. Wheeler 2nd, E. Casiano, N. Y. Siddiqui, H. S. Harvie, M. M. Mamik, E. M. Balk, V. W. Sung and T. L. Wheeler, 2nd (2013). "Nonsurgical management of heavy menstrual bleeding: a systematic review." Obstetrics & Gynecology 121(3): 632-643.
231. Matteson, K. A., J. Valcin, C. A. Raker and M. A. Clark (2023). "A randomized trial comparing the 52-mg levonorgestrel system with combination oral contraceptives for treatment of heavy menstrual bleeding." American journal of obstetrics and gynecology 229(5): 532.e531-532.e51
232. Mawet, M., F. Nollevaux, D. Nizet, F. Wijzen, V. Gordenne, N. Tasev, D. Segedi, B. Marinescu, A. Enache, V. Parhomenko, F. Frankenne and J.-M. Foidart (2014). "Impact of a new levonorgestrel intrauterine system, Levosert( R), on heavy menstrual bleeding: results of a one-year randomised controlled trial." The European journal of contraception & reproductive health care : the official journal of the European Society of Contraception 19(3): 169-179.
233. Meng, W., W. Lin, W. Yeung, Y. Zhang, H. Ng, Y. Lee, Z. Zhang, J. Rong and L. Lao (2021). "Randomized double-blind trial comparing low dose and conventional dose of a modified traditional herbal formula Guizhi Fuling Wan in women with symptomatic uterine fibroids." Journal of ethnopharmacology 283.
234. Mercorio, F., R. De Simone, A. Di Spiezio Sardo, G. Cerrota, G. Bifulco, F. Vanacore and C. Nappi (2003). "The effect of a levonorgestrel-releasing intrauterine device in the treatment of myoma-related menorrhagia." Contraception 67(4): 277-280.
235. Middleton, L. J., R. Champaneria, J. P. Daniels, S. Bhattacharya, K. G. Cooper, N. H. Hilken, P. O'Donovan, M. Gannon, R. Gray and K. S. Khan (2010). "Hysterectomy, endometrial destruction, and levonorgestrel releasing intrauterine system (Mirena) for heavy menstrual bleeding: Systematic review and meta-analysis of data from individual patients." BMJ (Online) 341(7769): 379.
236. Mir, S. A., R. Ara, F. Amin, A. Malik, L. Hamid, T. Ali, G. N. Bader, S. U. D. Wani, M. Almuqbil, S. Alshehri, A. M. Alshehri and F. Shakeel (2022). "Evaluation of the Safety and Efficacy of Ormeloxifene, a Selective Estrogen Receptor Modulator and Medroxyprogesterone Acetate in Women with Non-Structural Abnormal Uterine Bleeding: A Randomized Clinical Trial." Medicina (Kaunas, Lithuania) 58(11).
237. Mirzaei, N., F. S. Moghaddam, G. Ozgoli, S. Sahranavard and E. Ghasemi (2018). "Purslane (Portulaca oleracea) effect on heavy menstrual bleeding (menorrhagia) in Iranian women." Advances in Integrative Medicine 5(2): 56-62.
238. Mohamed, A., M. Magd El Din, F. Laila, M. Al Shaimaa, H. Reem and R. Alaa (2018). <The> use of levonorgestrel-releasing system [metraplant-E] in the treatment of abnormal uterine bleeding. 72: 3979-3985.
239. Mohite, R. V. and V. R. Mohite (2013). "Correlates of the menstrual problems among rural college students of Satara district." Al Ameen Journal of Medical Sciences 6(3): 213-218.
240. Mohite, R. V., V. R. Mohite, S. M. Kumbhar and P. Ganganahalli (2013). "Common menstrual problems among slum adolescent girls of western Maharashtra, India." Journal of Krishna Institute of Medical Sciences University 2(1): 89-97.
241. Mollazadeh, S., M. Mirghafourvand and N. G. Abdollahi (2020). "The effects of Vitex agnus-castus on menstrual bleeding: A systematic review and meta-analysis." Journal of Complementary and Integrative Medicine 17(1): 20180053.
242. Momoeda, M., S. Akira, T. Harada, J. Kitawaki, N. Maeda, I. Ota, K. Yoshihara and N. Takahashi (2022). "Quality of Life of Japanese Dysmenorrhea/Heavy Menstrual Bleeding Patients Treated with Levonorgestrel Intrauterine Delivery System in a Real-World Setting." Advances in therapy 39(8): 3616-3634.
243. Monteiro, I., L. Bahamondes, J. Diaz, M. Perrotti and C. Petta (2002). "Therapeutic use of levonorgestrel-releasing intrauterine system in women with menorrhagia: A pilot study." Contraception 65(5): 325-328.
244. Morabad, P. P., D. S. Natekar, S. H. Shanta, S. M. Pooja, H. B. Puneet, K. K. Muttu, M. P. Renuka and V. G. Jayshree (2024). "Prevalence of Menorrhagia and its Association Factors Quality of Life of Adolescents: A Cross-sectional Study." SSR Institute of International Journal of Life Sciences 10(4): 5974-5978.
245. Muakkeaw, T., B. Sumdaengrit and P. Pokpalagon (2024). "Sexual Health among Thai Women with Gynecological Problems at a University Hospital." Clinical and Experimental Obstetrics and Gynecology 51(5): 124.
246. Mukherjee, S., P. Mishra, Y. Anand, A. Garg and R. Agarwal (2024). "ACCEPTANCE OF MIRENA IN HEAVY MENSTRUAL BLEEDING: A PROSPECTIVE STUDY." Journal of Cardiovascular Disease Research 15(9): 121-129.
247. Munir, W., I. Qureshi and S. Javed (2019). "USE OF MIX-TREATMENT COMPARISON APPROACHES IN ASSESSING EFFICACY OF TREATMENT FOR HEAVY MENSTURAL BLEEDING." INDO AMERICAN JOURNAL OF PHARMACEUTICAL SCIENCES 6(3): 5512-5518.
248. Murat Naki, M., C. Tekcan, N. Ozcan and M. Cebi (2010). "Levonorgestrel-releasing intrauterine device insertion ameliorates leiomyoma-dependent menorrhagia among women of reproductive age without a significant regression in the uterine and leiomyoma volumes." Fertility and sterility 94(1): 371-374.
249. Muse, K., R. G. Mabey, A. Waldbaum, J. K. Gersten and T. L. Adomako (2012). "Tranexamic acid increases hemoglobin and ferritin levels in women with heavy menstrual bleeding." Journal of women's health (2002) 21(7): 756-761
250. Naafe, M., N. Kariman, Z. Keshavarz, N. Khademi, F. Mojab and A. Mohammadbeigi (2018). "Effect of Hydroalcoholic Extracts of Capsella Bursa-Pastoris on Heavy Menstrual Bleeding: A Randomized Clinical Trial." Journal of alternative and complementary medicine (New York, N.Y.) 24(7): 694-700.
251. Nabia, T., A. Rukhsana, J. Tara, R. Faisal, N. Fauzia and K. Maryam (2011). Efficacy of levonorgestrel intrauterine system [LNG-IUS] for abnormal uterine bleeding and contraception. 21: 210-213.
252. Nabwera HM, Shah V, Neville R, Sosseh F, Saidykhan M, Faal F, Sonko B, Keita O, Schmidt WP, Torondel B. Menstrual hygiene management practices and associated health outcomes among school-going adolescents in rural Gambia. PLoS One. 2021 Feb 25;16(2):e0247554. doi: 10.1371/journal.pone.0247554. PMID: 33630924; PMCID: PMC7906402.
253. Naeema, U. and F. Fouzia (2012). Levenorgestrel Intra utrine system [LNG IUS] in menorrhagia: a three years followup study. 26: 79-83.
254. Nagrani, R., P. Bowen-Simpkins and J. W. Barrington (2002). "Can the levonorgestrel intrauterine system replace surgical treatment for the management of menorrhagia?" BJOG : an international journal of obstetrics and gynaecology 109(3): 345-347.
255. Naib, J. M., F. Afridi and M. Qadir (2023). "LEVONORGESTREL RELEASING INTRAUTERINE SYSTEM (MIRENA) FOR ABNORMAL UTERINE BLEEDING-A USEFUL TOOL IN THE COVID TIMES." Journal of Medical Sciences (Peshawar) 31(3): 199-202.
256. Najam, R., D. Agarwal, R. Tyagi and S. Singh (2010). "Comparison of traneximic acid with a combination of traneximic acid and mefenamic acid in reducing menstrual blood loss in ovulatory dysfunctional uterine bleeding (DUB)." Journal of Clinical and Diagnostic Research 4(5): 3020-3025.
257. Nampoothiri, S. K., A. Sreedhar and A. S. Hameed (2021). "EFFECT OF COMBINATION OF FRUIT OF BADARA [Ziziphus jujuba (L). Lam] AND PALM JAGGERY IN DYSFUNCTIONAL UTERINE BLEEDING." International Journal of Research in Ayurveda and Pharmacy 13(1): 32-36.
258. Naoulou, B. and M. C. Tsai (2012). "Efficacy of tranexamic acid in the treatment of idiopathic and non-functional heavy menstrual bleeding: a systematic review." Acta obstetricia et gynecologica Scandinavica 91(5): 529-537.
259. Nathany, S., S. Das, K. K. Patra and K. P. Madhwani (2023). "A study to compare the efficacy of Ormaloxifene vs mifepristone in the management of uterine leiomyoma." European Journal of Cardiovascular Medicine 13(3): 1681-1690.
260. Nidhi, A. Kumari, S. Tirkey and J. Prakash (2022). "Effectiveness of Levonorgestrel Releasing Intrauterine System in Perimenopausal Women with Heavy Menstrual Bleeding: A Prospective Study at a Teaching Hospital in India." Journal of menopausal medicine 28(3): 128-135.
261. Nie, L., H. Zou, X. Ma, L. Cheng, J. Jiao, F. Wang, W. Liang and P. Zhang (2021). "A clinical observational study on the efficacy of subcutaneous etonogestrel implants for adenomyosis in 20 patients." Gynecological endocrinology : the official journal of the International Society of Gynecological Endocrinology 37(8): 735-739.
262. Nooh, A. M. (2015). "Menstrual disorders among Zagazig University Students, Zagazig, Egypt." Middle East Fertility Society Journal 20(3): 198-203.
263. Nwagha, T. U., H. C. Okoye, A. O. Ugwu and E. O. Ugwu (2021). "Clinical screening for menorrhagia and other bleeding symptoms in Nigerian women." Annals of African medicine 20(2): 111-115.
264. O'Brien, S. H., S. Saini, H. Ziegler, M. Christian-Rancy, S. Ahuja, K. Hege, S. L. Savelli and S. K. Vesely (2019). "An Open-Label, Single-Arm, Efficacy Study of Tranexamic Acid in Adolescents with Heavy Menstrual Bleeding." Journal of pediatric and adolescent gynecology 32(3): 305-311.
265. Omani Samani, R., A. Almasi Hashiani, M. Razavi, S. Vesali, M. Rezaeinejad, S. Maroufizadeh and M. Sepidarkish (2018). "The prevalence of menstrual disorders in Iran: A systematic review and meta-analysis." International journal of reproductive biomedicine 16(11): 665-678.
266. Omidvar, S., F. N. Amiri, A. Bakhtiari and K. Begum (2018). "A study on menstruation of Indian adolescent girls in an urban area of South India." Journal of family medicine and primary care 7(4): 698-702.
267. Osama, S., E. S. Waleed, A. Ahmad N, S. Waleed and E. D. Ashraf (2009). Treatment of menorrhagia associated with uterine leiomyoma with the levonorgestrel-releasing intrauterine system. 77: 89-95.
268. Osuga, Y., K. Enya, K. Kudou, M. Tanimoto and H. Hoshiai (2019). "Oral gonadotropin-releasing hormone antagonist relugolix compared with leuprorelin injections for uterine leiomyomas: A randomized controlled trial." Obstetrics and gynecology 133(3): 423-433.
269. Osuga, Y., Y. Nakano, Y. Yamauchi and M. Takanashi (2021). "Ulipristal acetate compared with leuprorelin acetate for Japanese women with symptomatic uterine fibroids: a phase III randomized controlled trial." Fertility and sterility 116(1): 189-197.
270. Ozberk, H., D. Bilgic and A. Badem (2023). "Menstrual cycle abnormalities in women: characteristics, perceptions, and health-seeking behaviours." European Journal of Contraception and Reproductive Health Care 28(6): 301-307.
271. Patel, H. B., S. T. Soni, A. Bhagyalaxmi and N. M. Patel (2019). "Menstrual disorders and quality of life of women in an urban area of Puducherry: A community-based cross-sectional study." Journal of family medicine and primary care 8(1): 154-159.
272. Patel, N. K. and M. R. Pandya (2012). "A comparative study of tranexamic acid and ethamsylate in menorrhagia."
273. Patel, S. D., H. K. Jadeja and B. B. Airao (2023). "Study to Evaluate the Efficiency of Tranexamic Acid & Mefenamic Acid in DUB." International Journal of Toxicological and Pharmacological Research 13(8): 37-41.
274. Patela, N. K., S. Patelb, R. Damorc and M. R. Pandyad (2012). "Comparison of the efficacy and safety of norethisterone vs. combined oral contraceptive pills for the management of puberty menorrhagia."
275. Peng, F.-S., M.-Y. Wu, J.-H. Yang, S.-U. Chen, H.-N. Ho and Y.-S. Yang (2010). "Insertion of the Mirena intrauterine system for treatment of adenomyosis-associated menorrhagia: a novel method." Taiwanese journal of obstetrics & gynecology 49(2): 160-164.
276. Pham, K. H., T. H. Nguyen, T. Vi, H. H. V. Ly, T. M. Phung, D. N. P. Pham and S. J. Cooper (2024). "Menstrual Cycle Characteristics and Relative Factors Among Vietnamese Female Medical Students: A Cross-Sectional Study During the COVID-19 Pandemic." CURRENT WOMENS HEALTH REVIEWS
277. Pouraliroudbaneh, S., J. Marino, E. Riggs, A. Saber, Y. Jayasinghe and M. Peate (2024). "Heavy menstrual bleeding and dysmenorrhea in adolescents: A systematic review of self-management strategies, quality of life, and unmet needs." International Journal of Gynecology and Obstetrics 167(1): 16-41.
278. Qaraaty, M., S. H. Kamali, F. H. Dabaghian, N. Zafarghandi, R. Mokaberinejad, M. Mobli, G. Amin, M. Naseri, M. Kamalinejad, M. Amin, A. Ghaseminejad, S. J. HosseiniKhabiri and D. Talei (2014). "Effect of myrtle fruit syrup on abnormal uterine bleeding: a randomized double-blind, placebo-controlled pilot study." Daru : journal of Faculty of Pharmacy, Tehran University of Medical Sciences 22: 45.
279. Qiu, J., J. Cheng, Q. Wang and J. Hua (2014). "Levonorgestrel-releasing intrauterine system versus medical therapy for menorrhagia: a systematic review and meta-analysis." Medical science monitor : international medical journal of experimental and clinical research 20: 1700-1713.
280. Quraishi, S. R., V. B. Waghachavare, A. D. Gore and G. B. Dhumale (2015). "Are menstrual problems associated with the mental health? A cross sectional study among the graduation college girls." International Medical Journal Malaysia 14(2): 53-60.
281. Rahi, P., M. Mirghafourvand, S. Mohammad-Alizadeh-Charandabi and Y. Javadzadeh (2017). "Effects of mefenamic acid versus quince on menorrhagia and quality of life: A randomized controlled trial." Iranian Red Crescent medical journal 19(10): e16769.
282. Rahi, P., M. Mirghafourvand, S. Mohammad-Alizadeh-Charandabi, Y. Javadzadeh and S. Seidi (2016). "Comparison of the effect of mefenamic acid and quince on the level of menstrual bleeding and hemoglobin: A randomized controlled clinical trial." European Journal of Integrative Medicine 8(1): 67-72.
283. Rahman, S., F. S. Khan, K. A. Samin, N. Afridi and M. Ahmed (2021). "Efficacy of Oral Tranexamic Acid Versus Combined Oral Contraceptives for Heavy Menstrual Bleeding." Cureus 13(10): e19122.
284. Rahmani, E., S. Ahmadi, N. Motamed and F. Safinejad (2017). "Comparison the effect of letrozole versus medroxy progesterone acetate on premenopausal patients with endometrial hyperplasia: An randomized clinical trial." Crescent Journal of Medical and Biological Sciences 4(3): 99-103.
285. Rama, R., P. B. Shah, E. Shanthi, G. Palani and B. W. C. Sathiyasekaran (2018). "Social impact of menstrual problems among adolescent school girls in rural Tamil Nadu." International journal of adolescent medicine and health 30(5): 20160088.
286. Rani, M. and K. Jha (2024). "Role of Ulipristal Acetate in the Conservative Management of Uterine Fibroid." International Journal of Pharmaceutical and Clinical Research 16(5): 1943-1947.
287. Rani, M., U. Singh, G. G. Agrawal, S. M. Natu, S. Kala, A. Ghildiyal and N. Srivastava (2013). "Impact of Yoga Nidra on menstrual abnormalities in females of reproductive age." Journal of alternative and complementary medicine (New York, N.Y.) 19(12): 925-929.
288. Rathod, A. D., R. P. Chavan, S. P. Pajai, V. Bhagat and P. Thool (2016). "Gynecological Problems of Adolescent Girls Attending Outpatient Department at Tertiary Care Center with Evaluation of Cases of Puberty Menorrhagia Requiring Hospitalization." Journal of Obstetrics and Gynecology of India 66(Supplement 1): 400-406.
289. Rauramo, I., I. Elo and O. Istre (2004). "Long-term treatment of menorrhagia with levonorgestrel intrauterine system versus endometrial resection." Obstetrics and gynecology 104(6): 1314-1321.
290. Ravi, R., P. Shah, G. Palani, S. Edward and B. W. C. Sathiyasekaran (2016). "Prevalence of Menstrual Problems among Adolescent School Girls in Rural Tamil Nadu." Journal of pediatric and adolescent gynecology 29(6): 571-576.
291. Ray, S. and A. Ray (2014). "Non-surgical interventions for treating heavy menstrual bleeding (menorrhagia) in women with bleeding disorders." The Cochrane database of systematic reviews(11): CD010338.
292. Reddy, P. P., M. Chukka, K. Geetha and Sindhuja (2023). "COMPARISON OF EFFECTIVENESS OF ORMELOXIFENE WITH NORETHISTERONE IN TREATMENT OF AUB." European Journal of Molecular and Clinical Medicine 10(1): 561-569.
293. Reid, P. C. and S. Virtanen-Kari (2005). "Randomised comparative trial of the levonorgestrel intrauterine system and mefenamic acid for the treatment of idiopathic menorrhagia: a multiple analysis using total menstrual fluid loss, menstrual blood loss and pictorial blood loss assessment charts." BJOG : an international journal of obstetrics and gynaecology 112(8): 1121-1125.
294. Rezende, G. P., D. A. Y. Gomes and C. L. Benetti-Pinto (2023). "Abnormal uterine bleeding in reproductive age: a comparative analysis between the five Brazilian geographic regions." Revista da Associacao Medica Brasileira (1992) 69(suppl 1): e2023S2111.
295. Rezende, G. P., D. A. Y. Gomes and C. L. Benetti-Pinto (2023). "Prevalence of abnormal uterine bleeding in Brazilian women: Association between self-perception and objective parameters." PloS one 18(3 March): e0282605.
296. Romer, T. (2000). "Prospective comparison study of levonorgestrel IUD versus Roller-Ball endometrial ablation in the management of refractory recurrent hypermenorrhea." European journal of obstetrics, gynecology, and reproductive biology 90(1): 27-29.
297. Roth, L. P., K. M. Haley and M. K. Baldwin (2022). "A Retrospective Comparison of Time to Cessation of Acute Heavy Menstrual Bleeding in Adolescents Following Two Dose Regimens of Combined Oral Hormonal Therapy." Journal of pediatric and adolescent gynecology 35(3): 294-298.
298. Rovelli, R. J., N. E. Cieri-Hutcherson and T. C. Hutcherson (2022). "Systematic review of oral pharmacotherapeutic options for the management of uterine fibroids." Journal of the American Pharmacists Association 62(3): 674-682.e675.
299. Saffarieh, E., S. Nassiri, E. Jahan, B. Tarahom and H. Firouzi (2020). "What are the affecting factors on the quality of life and level of satisfaction and comfort in patients suffering from abnormal uterine bleeding?" Crescent Journal of Medical and Biological Sciences 7(2): 207-211.
300. Sahasikdar, S., S. Roy, K. R. Rahaman and B. Ghosh (2023). "Comparing Combined Pills to Progesterone-Only Pills for Abnormal Uterine Bleeding in Perimenopausal Women." International Journal of Pharmaceutical and Clinical Research 15(9): 1332-1345.
301. Sahin, H., A. Gungoren, B. Sezgin, B. Un, E. A. Sahin, K. Dolapcioglu and R. N. Bayik (2021). "Vascular effect of levonorgestrel intrauterine system on heavy menstrual bleeding: is it associated with hemodynamic changes in uterine, radial, and spiral arteries?" Journal of obstetrics and gynaecology : the journal of the Institute of Obstetrics and Gynaecology 41(1): 89-93.
302. Santos, I. S., G. C. Minten, N. C. Valle, G. C. Tuerlinckx, A. B. Silva, G. A. Pereira and J. F. Carriconde (2011). "Menstrual bleeding patterns: A community-based cross-sectional study among women aged 18-45 years in Southern Brazil." BMC women's health 11(1): 26.
303. Sarbhai, V. and S. Kumari (2023). "Ormeloxifene: The Perfect Alternative for the First-line Management of Abnormal Uterine Bleeding." Journal of SAFOG 15(1): 85-87.
304. Sarwar, U. and U. Rauf (2021). "SOCIAL SUPPORT, QUALITY OF LIFE AND MENTAL HEALTH PROBLEMS AMONG FEMALES WITH AND WITHOUT MENSTRUATION PROBLEMS: A COMPARATIVE STUDY." Khyber Medical University Journal 13(4): 206-210.
305. Sayed, G. H., M. S. Zakherah, S. A. El-Nashar and M. M. Shaaban (2011). "A randomized clinical trial of a levonorgestrel-releasing intrauterine system and a low-dose combined oral contraceptive for fibroid-related menorrhagia." International journal of gynaecology and obstetrics: the official organ of the International Federation of Gynaecology and Obstetrics 112(2): 126-130.
306. Schlaff, W. D., R. T. Ackerman, A. Al-Hendy, D. F. Archer, K. T. Barnhart, L. D. Bradley, B. R. Carr, E. C. Feinberg, S. M. Hurtado, K. JinHee, L. Ran, R. G. Mabey Jr, C. D. Owens, A. Poindexter, E. E. Puscheck, H. Rodriguez-Ginorio, J. A. Simon, A. M. Soliman, E. A. Stewart and N. B. Watts (2020). "Elagolix for Heavy Menstrual Bleeding in Women with Uterine Fibroids." New England Journal of Medicine 382(4): 328-340.
307. Selmi, M., L. Lassoued, B. Bannour, C. Chbili, M. Ben Fredj, R. Charfeddin and H. Khairi (2022). "Therapeutic effect of herbal infusion on abnormal uterine bleeding: interventional non-randomized pilot study." F1000Research 11: 1116.
308. Sesti, F., R. Piancatelli, A. Pietropolli, V. Ruggeri and E. Piccione (2012). "Levonorgestrel-releasing intrauterine system versus laparoscopic supracervical hysterectomy for the treatment of heavy menstrual bleeding: a randomized study." Journal of women's health (2002) 21(8): 851-857.
309. Shaaban, M. M., M. S. Zakherah, S. A. El-Nashar and G. H. Sayed (2011). "Levonorgestrel-releasing intrauterine system compared to low dose combined oral contraceptive pills for idiopathic menorrhagia: a randomized clinical trial." Contraception 83(1): 48-54.
310. Shafiee, M., A. Heidari, H. Amouzegar, S. Khani and F. Nojavan (2019). ""Evaluation of the effect of roasted lentil flour (lentil savigh) as a functional food in menstrual bleeding reduction"." Complementary therapies in medicine 44: 27-31.
311. Shah, H. N., P. M. Joshi, J. Shukla and V. Patel (2024). "Use of Norethisterone in Management of Dysfunctional Uterine Bleeding." International Journal of Pharmaceutical and Clinical Research 16(6): 682-687.
312. Shan, J., H. Tian, C. Zhou, H. Wang, X. Ma, R. Li, H. Yu, G. Chen, J. Zhu, Z. Cai, C. Lin, L. Cheng, Y. Xu, S. Liu, C. Zhang, Q. Luo, Y. Zhang, S. Jin, C. Liu, Q. Zhang, L. Lv, L. Yang, J. Chen, Q. Li, W. Liu, W. Yue, X. Song and C. Zhuo (2022). "Prevalence of Heavy Menstrual Bleeding and Its Associated Cognitive Risks and Predictive Factors in Women With Severe Mental Disorders." Frontiers in pharmacology 13: 904908.
313. Shang, Y., S. Lu, Y. Chen and X. Sun (2018). "Chinese herbal medicines for the treatment of non-structural abnormal uterine bleeding in perimenopause: A systematic review and a meta-analysis." Complementary therapies in medicine 41: 252-260.
314. Sharma, J. B., R. Kumari, S. Kumari, S. Jain and S. Dharmendra (2023). "To Study the Efficacy and Safety of Diosmin with Tranexamic Acid and Mefenamic Acid Versus only Tranexamic Acid and Mefenamic Acid in Medical Management of Abnormal Uterine Bleeding: A Randomized Controlled Trial." Journal of mid-life health 14(2): 87-93.
315. Shaw, R. W., I. M. Symonds, O. Tamizian, J. Chaplain and S. Mukhopadhyay (2007). "Randomised comparative trial of thermal balloon ablation and levonorgestrel intrauterine system in patients with idiopathic menorrhagia." The Australian & New Zealand journal of obstetrics & gynaecology 47(4): 335-340.
316. Shaw, V., A. C. Vandal, C. Coomarasamy and A. J. Ekeroma (2016). "The effectiveness of the levonorgestrel intrauterine system in obese women with heavy menstrual bleeding." The Australian & New Zealand journal of obstetrics & gynaecology 56(6): 619-623.
317. Shawki, O., W. El-Sherbiny, W. Saber, A. Askalany and A. El-Daly (2009). "Treatment of heavy menstrual bleeding associated with uterine leiomyoma with the levonorgestrel-releasing intrauterine system." Gynecological surgery 6(4): 331-337.
318. Shipika (2023). "Ulipristal Acetate versus Placebo for Fibroid Treatment." International Journal of Toxicological and Pharmacological Research 13(9): 206-211.
319. Shobeiri, S. F., S. Sharei, A. Heidari and S. Kianbakht (2009). "Portulaca oleracea L. in the treatment of patients with abnormal uterine bleeding: a pilot clinical trial." Phytotherapy research : PTR 23(10): 1411-1414.
320. Shrestha, D., S. Aryal, A. Tiwari and R. Sharma (2022). "Abnormal Uterine Bleeding among Women Visiting Gynecology Out- patient Department of a Tertiary Care Hospital: A Descriptive Cross- sectional Study." JNMA; journal of the Nepal Medical Association 60(246): 121-125.
321. Silva, A. L., F. D. N. Pereira, S. S. de Souza, L. F. Loures, A. P. C. Rocha, C. N. Valadares, M. M. Carneiro, R. L. C. Tavares and A. F. Camargos (2013). "Five-year follow-up of levonorgestrel-releasing intrauterine system versus thermal balloon ablation for the treatment of heavy menstrual bleeding: a randomized controlled trial." Contraception 87(4): 409-415.
322. Simon, J. A., A. Al-Hendy, D. F. Archer, K. T. Barnhart, L. D. Bradley, B. R. Carr, T. Dayspring, E. C. Feinberg, V. Gillispie, S. Hurtado, J. Kim, R. Liu, C. D. Owens, O. Muneyyirci-Delale, A. Wang, N. B. Watts and W. D. Schlaff (2020). "Elagolix Treatment for Up to 12 Months in Women With Heavy Menstrual Bleeding and Uterine Leiomyomas." Obstetrics and gynecology 135(6): 1313-1326.
323. Singh, N., M. Faruqi and Y. Pradeep (2019). "Clinico epidemiological profile of abnormal uterine bleeding in reproductive womens: a cross sectional study." 8.
324. Sinharoy, S. S., L. Chery, M. Patrick, A. Conrad, A. Ramaswamy, A. Stephen, J. Chipungu, Y. M. Reddy, R. Doma, S.-R. Pasricha, T. Ahmed, C. B. Chiwala, N. Chakraborti and B. A. Caruso (2023). "Prevalence of heavy menstrual bleeding and associations with physical health and wellbeing in low-income and middle-income countries: a multinational cross-sectional study." The Lancet. Global health 11(11): e1775-e1784.
325. Socolov, D., I. Blidaru, B. Tamba, N. Miron, L. Boiculese and R. Socolov (2011). "Levonorgestrel releasing-intrauterine system for the treatment of menorrhagia and/or frequent irregular uterine bleeding associated with uterine leiomyoma." The European journal of contraception & reproductive health care : the official journal of the European Society of Contraception 16(6): 480-487.
326. Sohail Mahmood, C. and L. Bushra (2017). Use of calcium dobesilate and norethisterone in treatment of menorrhagia of dysfunctional uterine bleeding origin. 22: 65-67.
327. Sonali, S. Kumari and G. Sinha (2020). "To determine the menstrual pattern and prevalence of various menstrualproblems: Cross-sectional study." European Journal of Molecular and Clinical Medicine 7(10): 3663-3667.
328. Sonam, S. Maheswari, R. Sharma and G. Gill (2023). "A PROSPECTIVE STUDY OF COMPARISION OF HORMONAL V/S NON-HORMONAL TREATMENT IN ABNORMAL UTERINE BLEEDING." International Journal of Current Pharmaceutical Research 15(4): 33-35.
329. Soontrapa, N., M. Rattanachaiyanont, M. Warnnissorn, T. Wongwananuruk, S. Indhavivadhana, P. Tanmahasamut, K. Techatraisak and S. Angsuwathana (2022). "The effectiveness of desogestrel for endometrial protection in women with abnormal uterine bleeding-ovulatory dysfunction: a non-inferiority randomized controlled trial." Scientific reports 12(1): 1662.
330. Srinil, S. and U. Jaisamrarn (2005). "Treatment of idiopathic menorrhagia with tranexamic acid." Journal of the Medical Association of Thailand = Chotmaihet thangphaet 88 Suppl 2: S1-6.
331. Srivaths, L. V., J. E. Dietrich, D. L. Yee, H. Sangi-Haghpeykar and D. Mahoney, Jr. (2015). "Oral Tranexamic Acid versus Combined Oral Contraceptives for Adolescent Heavy Menstrual Bleeding: A Pilot Study." Journal of pediatric and adolescent gynecology 28(4): 254-257.
332. Stewart, A., C. Cummins, L. Gold, R. Jordan and W. Phillips (2001). "The effectiveness of the levonorgestrel-releasing intrauterine system in menorrhagia: a systematic review." BJOG : an international journal of obstetrics and gynaecology 108(1): 74-86.
333. Stewart, E. A., A. Al-Hendy, A. S. Lukes, O. S. Madueke-Laveaux, E. Zhu, S. Proehl, T. Schulmann and E. E. Marsh (2024). "Relugolix combination therapy in Black/African American women with symptomatic uterine fibroids: LIBERTY Long-Term Extension study." American journal of obstetrics and gynecology 230(2): 237.e231-237.e211.
334. Stewart, E. A., D. F. Archer, C. D. Owens, K. T. Barnhart, L. D. Bradley, E. C. Feinberg, V. Gillispie-Bell, A. N. Imudia, R. Liu, J. H. Kim and A. Al-Hendy (2022). "Reduction of Heavy Menstrual Bleeding in Women Not Designated as Responders to Elagolix Plus Add Back Therapy for Uterine Fibroids." Journal of Women's Health 31(5): 698-705.
335. Stewart, E. A., M. P. Diamond, A. R. W. Williams, B. R. Carr, E. R. Myers, R. A. Feldman, W. Elger, C. Mattia-Goldberg, B. M. Schwefel and K. Chwalisz (2019). "Safety and efficacy of the selective progesterone receptor modulator asoprisnil for heavy menstrual bleeding with uterine fibroids: pooled analysis of two 12-month, placebo-controlled, randomized trials." Human reproduction (Oxford, England) 34(4): 623-634.
336. Su, S., X. Yang, Q. Su and Y. Zhao (2020). "Prevalence and knowledge of heavy menstrual bleeding among gynecology outpatients by scanning a WeChat QR Code." PloS one 15(4): e0229123.
337. Subrata, B., D. Manjulika and D. Rituparna (2014). "A study of reproductive health problems among rural adolescent girls of Mohanpur block of West Tripura district." Al Ameen Journal of Medical Sciences 7(1): 78-82.
338. Suji, U. and L. Perriera (2014). "Hormonal Contraception as Treatment for Heavy Menstrual Bleeding: A Systematic Review." Clinical Obstetrics & Gynecology 57(4): 694-717.
339. Sun-Ying, W. and Z. Hong-Yun (2015). "GnRHa therapy combined with Mirena for treatment of 70 cases of adenomyosis." Shanghai Journal of Preventive Medicine(12): 105-107.
340. Taim, B. C., C. Ó Catháin, M. Renard, K. J. Elliott-Sale, S. Madigan and N. Ní Chéilleachair (2023). "The Prevalence of Menstrual Cycle Disorders and Menstrual Cycle-Related Symptoms in Female Athletes: A Systematic Literature Review." Sports Medicine 53(10): 1963-1984.
341. Tam, W. H., P. M. Yuen, D. P. S. Ng, P. L. Leung, I. H. Lok and M. S. Rogers (2006). "Health status function after treatment with thermal balloon endometrial ablation and levonorgestrel intrauterine system for idiopathic menorrhagia: A randomized study." Gynecologic and obstetric investigation 62(2): 84-88.
342. Tellum, T., M. Omtvedt, J. Naftalin, M. Hirsch and D. Jurkovic (2021). "A systematic review of outcome reporting and outcome measures in studies investigating uterine-sparing treatment for adenomyosis." Human Reproduction Open 2021(3).
343. Theodoridis, T. D., L. Zepiridis, M. Zafrakas, G. Grimbizis, A. Tantsis, D. Kyrou and J. N. Bontis (2009). "Levonorgestrel-releasing intrauterine system vs. endometrial thermal ablation for menorrhagia." Hormones (Athens, Greece) 8(1): 60-64.
344. Titilayo, A., O. M. Agunbiade, O. Banjo and A. Lawani (2009). "Menstrual discomfort and its influence on daily academic activities and psychosocial relationship among undergraduate female students in Nigeria." Tanzania journal of health research 11(4): 181-188.
345. Tu, X., G. Huang and S. Tan (2009). "Chinese herbal medicine for dysfunctional uterine bleeding: A meta-analysis." Evidence-based Complementary and Alternative Medicine 6(1): 99-105.
346. Umarji, M. and P. S. Patki (2012). "Evaluation of Efficacy and Safety of Evecare Syrup in Menstrual Irregularities: A Multicentric, Post Marketing Surveillance Study."
347. Vaidya, R., S. Vinayachandran, S. Devi, B. Prejisha, G. Lekshminath, S. Sreedharan and P. K. Jahrin (2022). "Prevalence of Abnormal Uterine Bleeding and its Associated Risk Factors in Women of Perimenopausal Age Group-A Retrospective Study." Journal of Clinical and Diagnostic Research 16(12): QC09-QC13.
348. Vardaini, D., R. Mishra, R. Ranjan and S. R. Borade (2020). "Comparison of efficacy and safety of ormeloxifene and cyclical progesterone (Norethisterone) in ovulatory abnormal uterine bleeding." Asian Journal of Pharmaceutical and Clinical Research 13(11): 178-180.
349. Varghese, L., P. J. Prakash and V. Lekha (2019). "A study to identify the menstrual problems and related practices among adolescent girls in selected higher secondary school in Thiruvananthapuram, Kerala, India." Journal of SAFOG (South Asian Federation of Obstetrics and Gynaecology) 11(1): 13-16.
350. Varner, R. E., C. C. Ireland, R. L. Summitt Jr, H. E. Richter, L. A. Learman, E. Vittinghoff, M. Kuppermann, E. Washington and S. B. Hulley (2004). "Medicine or Surgery (Ms): A randomized clinical trial comparing hysterectomy and medical treatment in premenopausal women with abnormal uterine bleeding." Controlled clinical trials 25(1): 104-118.
351. Venkatachalam, S., J. S. Bagratee and J. Moodley (2004). "Medical management of uterine fibroids with medroxyprogesterone acetate (Depo Provera): A pilot study." Journal of Obstetrics and Gynaecology 24(7): 798-800.
352. Venturella, R., T. Rechberger, J. Zatik, R. B. Wagman, E. Zhu, V. G. Rakov and F. Petraglia (2023). "Relugolix combination therapy in European women with symptomatic uterine fibroids: a subgroup analysis from the randomized phase 3 LIBERTY pivotal trials." Gynecological Endocrinology 39(1): 2249107.
353. Vilos, G. A., J. Marks, V. Tureanu, B. Abu-Rafea and A. G. Vilos (2011). "The levonorgestrel intrauterine system is an effective treatment in selected obese women with abnormal uterine bleeding." Journal of minimally invasive gynecology 18(1): 75-80.
354. Wadgave, H. V., G. M. Jatti and S. Ahankari (2014). "Menstrual problems in adolescent girls of slum areas." Indian Journal of Public Health Research and Development 5(1): 69-71.
355. Waghachavare, V. B., V. M. Chavan and G. B. Dhumale (2013). "A study of menstrual problems among the female junior college students from rural area of Sangli District." National Journal of Community Medicine 4(2): 236-240.
356. Waheed, S. and A. Malik (2013). "Mirena as an Alternative to Hysterectomy in Cases of Dub." ANNALS OF KING EDWARD MEDICAL UNIVERSITY LAHORE PAKISTAN 19(2): 174-180.
357. Walraven, G., G. Ekpo, R. Coleman, C. Scherf, L. Morison and S. D. Harlow (2002). "Menstrual disorders in rural Gambia." Studies in family planning 33(3): 261-268.
358. Wasiak, R., A. Filonenko, D. J. Vanness, A. Law, M. Jeddi, K. U. Wittrup-Jensen, D. E. Stull, S. Siak and J. T. Jensen (2013). "Impact of estradiol valerate/dienogest on work productivity and activities of daily living in women with heavy menstrual bleeding." Journal of women's health (2002) 22(4): 378-384.
359. Wei, A., X. Tang, W. Yang, J. Zhou, W. Zhu and S. Pan (2024). "Efficacy of etonogestrel subcutaneous implants versus the levonorgestrel-releasing intrauterine system in the conservative treatment of adenomyosis." Open medicine (Warsaw, Poland) 19(1): 20240914.
360. Weisberg, E., K. McGeehan, J. Hangan and I. S. Fraser (2017). "Potentially effective therapy of heavy menstrual bleeding with an oestradiol-nomegestrol acetate oral contraceptive: a pilot study." Pilot and feasibility studies 3: 18.
361. Wheeler, T. L., 2nd, M. Murphy, R. G. Rogers, R. Gala, B. Washington, L. Bradley, K. Uhlig, B. E. M. A. H. H. O. B. M. T. K. S. S. S. J. S. V. W. Matteson Ka and G. Society of Gynecologic Surgeons Systematic Review (2012). "Clinical practice guideline for abnormal uterine bleeding: hysterectomy versus alternative therapy." Journal of minimally invasive gynecology 19(1): 81-88
362. Whitaker, L. H. R., L. J. Middleton, J. P. Daniels, A. R. W. Williams, L. Priest, S. Odedra, V. Cheed, C. E. Stubbs, T. J. Clark, M. A. Lumsden, D. K. Hapangama, S. Bhattacharya, P. P. Smith, E. P. Nicholls, N. Roberts, S. I. Semple, L. Saraswat, J. Walker, R. R. Chodankar and H. O. D. Critchley (2023). "UCON RCT: Ulipristal acetate versus levonorgestrel-releasing intrauterine system for heavy menstrual bleeding." BJOG-AN INTERNATIONAL JOURNAL OF OBSTETRICS AND GYNAECOLOGY 130: 27-28.
363. Whitaker, L. H. R., L. J. Middleton, J. P. Daniels, A. R. W. Williams, L. Priest, S. Odedra, V. Cheed, C. E. Stubbs, T. J. Clark, M.-A. Lumsden, D. K. Hapangama, S. Bhattacharya, P. P. Smith, E. P. Nicholls, N. Roberts, S. I. Semple, L. Saraswat, J. Walker, R. R. Chodankar, H. O. D. Critchley and C. Ucon (2023). "Ulipristal acetate versus levonorgestrel-releasing intrauterine system for heavy menstrual bleeding (UCON): a randomised controlled phase III trial." EClinicalMedicine 60: 101995.
364. Wildemeersch, D. and A. Andrade (2010). "Review of clinical experience with the frameless LNG-IUS for contraception and treatment of heavy menstrual bleeding." Gynecological endocrinology : the official journal of the International Society of Gynecological Endocrinology 26(5): 383-389.
365. Wildemeersch, D. and P. J. Rowe (2004). "Assessment of menstrual blood loss in women with ideopathic menorrhagia using the frameless levonorgestrel-releasing intrauterine system." Contraception 70(2): 165-168.
366. Wildemeersch, D. and P. J. Rowe (2005). "Assessment of menstrual blood loss in Belgian users of a new T-shaped levonorgestrel-releasing intrauterine system." Contraception 71(6): 470-473.
367. Wildemeersch, D., E. Schacht and P. Wildemeersch (2003). "Performance and acceptability of intrauterine release of levonorgestrel with a miniature delivery system for hormonal substitution therapy, contraception and treatment in peri and postmenopausal women." Maturitas 44(3): 237-245.
368. Xiao, B., S.-C. Wu, J. Chong, T. Zeng, L.-H. Han and T. Luukkainen (2003). "Therapeutic effects of the levonorgestrel-releasing intrauterine system in the treatment of idiopathic menorrhagia." Fertility and sterility 79(4): 963-969.
369. Xu, P., S. Ling, E. Hu, L. Ma, J. Liu and B. Yi (2024). "Efficacy of hysteroscopic levonorgestrel-releasing intrauterine device fixation in the treatment of adenomyosis: A cohort study." Biomedical reports 21(1): 109.
370. Yamini Shoba Vani, P., K. Nirmala and P. S. Jyothsna (2020). "Evaluation of the efficacy of tranexamic acid in the management of Menorrhagia." International Journal of Current Research and Review 12(24): 64-69.
371. Yang, B.-Q., J.-H. Xu and Y.-C. Teng (2015). "Levonorgestrel intrauterine system versus thermal balloon ablation for the treatment of heavy menstrual bleeding: A meta-analysis of randomized controlled trials." Experimental and therapeutic medicine 10(5): 1665-1674.
372. Yazbeck, C., S. Omnes, M. C. Vacher-Lavenu and P. Madelenat (2006). "[Levonorgestrel-releasing intrauterine system in the treatment of dysfunctional uterine bleeding: A French multicenter study]." Efficacite et tolerance du systeme intra-uterin au levonorgestrel dans la prise en charge des menorragies fonctionnelles: etude francaise multicentrique. 34(10): 906-913.
373. Yildizhan, R., B. P. Yildizhan, E. Adali and N. Suer (2004). "A levonorgestrel-releasing intrauterine system for the treatment of abnormal uterine bleeding." Marmara Medical Journal 17(2): 53-57.
374. Yousefi, F., M. Kashanian, I. Nazem, S. Bioos, O. Sadeghpour, J. Alias and F. Hashem-Dabaghian (2020). "Comparison between Golnar product and placebo in heavy menstrual bleeding: A double-blind randomized clinical trial." Avicenna journal of phytomedicine 10(5): 523-532.
375. Yu, Q., Y. Zhou, L. Suturina, U. Jaisamrarn, D. Lu and S. Parke (2018). "Efficacy and Safety of Estradiol Valerate/Dienogest for the Management of Heavy Menstrual Bleeding: A Multicenter, Double-Blind, Randomized, Placebo-Controlled, Phase III Clinical Trial." Journal of women's health (2002) 27(10): 1225-1232.
376. Yu, Y., Z. Zhou, L. Wang and J. Liu (2022). "Effect of Mirena Intrauterine Device on Endometrial Thickness, Quality of Life Score, and Curative Effect in Patients with Perimenopausal Abnormal Uterine Bleeding." Computational and mathematical methods in medicine 2022: 5648918.
